# Noninvasive detection and monitoring of glioblastoma subtypes via dual-marker plasma proteomics

**DOI:** 10.1093/noajnl/vdag015

**Published:** 2026-02-05

**Authors:** Patricia Rojas-Sanchez, Kirstine Juul-Elbaek, Henriette Pedersen, Dorte Schou Nørøxe, Aleena Azam, Hui Guo, Cong Zhou, Jiri Bartek, Jane Skjøth-Rasmussen, Ulrik Lassen, Erwin Schoff, Petra Hamerlik

**Affiliations:** Division of Cancer Sciences & Geoffrey Jefferson Brain Research Centre, University of Manchester, Manchester, United Kingdom; Brain Tumor Biology, Danish Cancer Institute, Copenhagen, Denmark; Brain Tumor Biology, Danish Cancer Institute, Copenhagen, Denmark; Department of Neurosurgery, Copenhagen University Hospital, Copenhagen, Denmark; Danish Comprehensive Cancer Center—Brain Tumor Center, Copenhagen, Denmark; Department of Neurosurgery, Copenhagen University Hospital, Copenhagen, Denmark; Danish Comprehensive Cancer Center—Brain Tumor Center, Copenhagen, Denmark; Division of Cancer Sciences & Geoffrey Jefferson Brain Research Centre, University of Manchester, Manchester, United Kingdom; CRUK National Biomarker Centre, Manchester, United Kingdom; Department of Neurosurgery, Copenhagen University Hospital, Copenhagen, Denmark; Department of Neurosurgery, Karolinska University Hospital and Karolinska Institutet, Stockholm, Sweden; Department of Clinical Neuroscience, Karolinska University Hospital and Karolinska Institutet, Stockholm, Sweden; Department of Neurosurgery, Copenhagen University Hospital, Copenhagen, Denmark; Department of Neurosurgery, Copenhagen University Hospital, Copenhagen, Denmark; Technical University of Denmark, Lyngby, Denmark; Division of Cancer Sciences & Geoffrey Jefferson Brain Research Centre, University of Manchester, Manchester, United Kingdom; Brain Tumor Biology, Danish Cancer Institute, Copenhagen, Denmark

**Keywords:** cartilage oligomeric matrix protein, coagulation factor IX, dual-marker assay, glioblastoma, plasma proteomics

## Abstract

**Background:**

Glioblastoma (GBM) is the most common and lethal primary brain tumor in adults, characterized by rapid progression and profound molecular heterogeneity. Current diagnostic and monitoring strategies rely on neuroimaging and invasive tissue sampling, which are limited in their ability to capture dynamic disease states and subtype-specific biology. There is an unmet need for minimally invasive biomarkers that can enable reliable detection and longitudinal surveillance. In this study, we investigated whether plasma proteomic profiling could reflect tumor-intrinsic features and systemic responses, thereby supporting noninvasive classification and monitoring of GBM subtypes.

**Methods:**

We performed integrative proteomic profiling of matched GBM tumor and plasma samples using tandem mass tag-labeled mass spectrometry and machine learning. Pathway and weighted gene co-expression network analyses were applied to identify systemic alterations. A dual-marker classifier was developed and validated using longitudinal aptamer-based profiling.

**Results:**

Tumor proteomes exhibited extensive heterogeneity, while plasma profiles showed marked interpatient homogeneity at both diagnosis and recurrence. Systemic changes were observed in inflammation, coagulation, and complement signaling pathways. A dual-marker plasma classifier comprising coagulation factor IX (F9) and cartilage oligomeric matrix protein (COMP) distinguished GBM from healthy controls with high accuracy (area under the curve AUC = 0.96), maintaining performance in recurrent disease (AUC = 0.97). Longitudinal analysis revealed divergent trajectories: F9 levels declined post-treatment, while COMP increased, consistent with therapeutic response and disease progression.

**Conclusions:**

Our findings support the development of a dual-marker, proteomics-based plasma assay for noninvasive GBM detection and real-time monitoring. This approach has the potential to complement imaging and inform therapeutic decision-making.

Key Points

- Dual-marker plasma assay detects GBM with high accuracy.
- F9 and COMP show opposing trends during treatment.
- Plasma proteome remains stable across GBM recurrence.

Importance of the Study

Glioblastoma (GBM) is marked by profound interpatient and intrapatient heterogeneity, complicating efforts to develop reliable biomarkers. This study demonstrates that a dual-marker plasma proteomics assay—based on coagulation factor IX (F9) and cartilage oligomeric matrix protein (COMP)—can robustly detect GBM across disease stages with high accuracy. Remarkably, the plasma proteome remains stable and consistent even as tumor tissue undergoes dynamic molecular changes, enabling detection and monitoring despite the tumor’s complexity. These findings highlight the translational potential of systemic proteomic signatures for noninvasive GBM surveillance and suggest a clinically accessible tool to complement imaging and guide therapeutic decisions.

Glioblastoma (GBM) remains one of the most aggressive and lethal brain malignancies, with limited therapeutic options and poor prognosis.^1^ Timely and accurate diagnosis, along with longitudinal monitoring of disease progression and recurrence, is critical for improving patient outcomes. However, current diagnostic modalities primarily reliant on imaging and invasive tissue biopsies, are constrained by limited sensitivity, sampling bias, and the inability to capture tumor heterogeneity or dynamic changes over time.^2^^,^^3^

Liquid biopsy has emerged as a promising, minimally invasive alternative for real-time tumor profiling and disease surveillance. By analyzing tumor-derived analytes in biofluids such as blood, plasma, or cerebrospinal fluid (CSF), liquid biopsies offer the potential to detect and monitor disease progression, recurrence, and treatment response. In GBM, circulating tumor DNA (ctDNA), cell-free RNA, extracellular vesicles (EVs), and metabolites have all been explored as candidate biomarkers.^4-13^ However, the clinical utility of nucleic acid-based biomarkers remains limited by their low abundance in circulation, particularly due to the restrictive nature of the blood-brain barrier and the often necrotic, hypovascular nature of GBM.^14^

Proteomics is increasingly recognized as a powerful frontier in precision oncology, offering a dynamic, systems-level view of tumor biology and host response.^15-17^ Unlike genomic and epigenomic profiling, which capture static or regulatory aspects of disease, proteomics reflects the functional state of cells and tissues in real-time, capturing the influence of the tumor microenvironment or systemic immune responses—factors that are increasingly recognized as critical in GBM progression and recurrence.^18-20^ Over the past decade, several studies have applied proteomic technologies to identify protein biomarkers for GBM underscoring the potential of proteomics to uncover clinically relevant biomarkers that are otherwise undetectable through genomic or transcriptomic methods.^21-37^ Recently, studies by Liu et al.^29^ and Clavreul et al.^37^ provided important insights into circulating protein signatures but were limited by small cohorts and the lack of longitudinal validation. Furthermore, these studies rely on conventional statistical thresholds (eg, fold-change or *P*-value cut-offs) that may obscure biologically meaningful patterns, particularly in high-dimensional datasets. Our study addresses these gaps by (1) leveraging a substantially larger, clinically annotated cohort with matched plasma and tumor samples, (2) applying network-based approaches to contrast systemic and local proteomic architecture, (3) using machine learning for biomarker selection, and (4) validating findings in an independent longitudinal cohort. We introduce a dual-marker plasma classifier (F9 and COMP) that demonstrates robust performance across primary and recurrent disease, offering a translationally relevant framework for noninvasive GBM detection and monitoring.

## Methods

### Study Cohort and Mass Spectrometry Profiling

Snap frozen tissue and EDTA plasma samples from adult GBM patients (for clinical characteristics see Supplementary Tables S1 and S2) and EDTA plasma samples from age and gender matched healthy volunteers with no prior history of cancer were collected at the Copenhagen University Hospital, Department of Neurosurgery, between 2010 and 2020. Whole blood was processed (within 1 h of collection) for plasma by centrifuging samples at 1900 × g for 10 min at 4°C and stored at −80 °C. Sample preparation and digestion were performed as previously described by Bonde et al.^38^ The mass spectrometry (MS) data acquisition was performed as previously described by Bonde et al. with minor changes.^38^ Each fraction was analyzed using the pre-set “30 samples per day” method on the Evosep One platform. For details, see Supplementary Methods.

### ML for Biomarker Discovery

ML analyses were performed on proteomic data following filtering and quality control. Highly correlated features were first identified using stats::cor() with the “pairwise.complete.obs” option on nonimputed data. Features with pairwise correlations above 0.9 were removed using caret::findCorrelation() (version 7.0.1) to reduce redundancy. Missing values in the remaining dataset were imputed as previously described. The dataset was split into training (85%) and validation (15%) sets using caret::createDataPartition(). A nested cross-validation (CV) framework was implemented using the nestedcv package (version 0.8.0) via glmnet (version 4.1.8) with option alpha = 1 for LASSO regression. The outer loop used leave-one-out cross-validation (LOOCV) with 10 folds (n_outer_folds = 10, outer_method = “LOOCV”), and feature selection was performed using filterFun = “ranger_filter” with class balancing via random sampling. Nineteen nested CV runs were conducted, testing models with 2 to 20 top-ranked features. Model performance was evaluated using accuracy, balanced accuracy, sensitivity, specificity, and area AUC, calculated with caret, stats and pROC (version 1.18.5) packages. The final model was selected based on the highest AUC and accuracy and validated on the held-out 15% of samples. Model robustness and feature stability were further assessed using nestedcv::repeatcv() with 100 repetitions. A new model with F9 and COMP, was built to evaluate their predictive power in distinguishing primary GBM (*n* = 57) from a randomly selected subset of healthy controls. This model was validated on recurrent GBM samples (*n* = 47) and the remaining healthy controls.

### Cell Type Deconvolution Analysis

Microenvironment Cell Populations-counter method was applied through the function MCPcounter.estimate() (version 1.2.0) to quantify the abundance of immune cell populations in GBM and healthy individuals’ proteome. This function produces a matrix with abundance estimates per sample per immune cell population. These abundances are specifically designed for comparative analysis within a dataset rather than for direct comparisons between different cell types. To calculate the immune abundance, estimate in the proteomic data, the immune cell type markers from 2 different curated datasets were used. Plots were generated using the log2 values of the abundance estimates produced by MCPcounter.estimate().

### Network-based Module Discovery via WGCNA

WGCNA (version 1.73)^39^ was performed independently on proteomic abundance matrices from tissue and plasma samples to identify modules of co-expressed proteins. Networks were constructed using the networkType = “signed” option to capture directional correlations relevant to GBM biology. The scale-free topology index across a range of soft-thresholding powers was evaluated, and the power parameter was selected as the lowest value for which the scale-free topology fit (*R*^2^) exceeded 0.7 or 0.8 (RsquaredCut = 0.7; RsquaredCut = 0.8). The co-expression modules were calculated using the function WGCNA::blockwiseModules() with mergeCutHeight = 0.25. From them, the module eigengenes (MEs) were identified. The correlation between modules and GBM status was estimated using the function WGCNA::corPvalueStudent().

### Longitudinal Biomarker Validation

To validate F9 and COMP as biomarkers, plasma samples from an independent longitudinal GBM cohort were analysed using SomaLogic’s SOMAscan platform (v5.0, 11K). Data were processed in R using SomaDataIO and SomaScan.db. Temporal dynamics were assessed via mixed-effects linear modeling (lmerTest), with time and treatment as fixed effects and patient ID as a random intercept. Model significance was evaluated using standard statistical metrics and ANOVA. Predicted biomarker trajectories were visualized using the predict() function.

## Results

### Patient Characteristics

This retrospective study (Figure 1A) included 215 subjects with 201 tissue samples (125 primary and 76 recurrent GBMs), and 132 plasma samples (57 primary GBMs/pGBM, 45 recurrent GBMs/reGBM, and 30 healthy controls). All GBM patients were World Health Organisation (WHO) grade 4 IDH1/2 wild-type gliomas. 55 patients had matched plasma and tissue samples, with 31 matched primary-recurrent pairs in the tissue cohort, and 29 in the plasma cohort. Among tissue samples, 59% of primary and 54% of recurrent cases were male. In the plasma cohort, males accounted for 56% of primary and 53% of recurrent cases. The majority of patients were aged between 40 and 79 years, with the 60-79 age group being the most represented (52% of primary and 43% of recurrent tissue cases; 49% in both primary and recurrent plasma cases). Patients under 40 or over 80 years were rare. Tumor location was evenly distributed between the left and right hemispheres, predominantly in the temporal and frontal lobes, followed by parietal and occipital regions. Complete surgical resection was more frequently achieved in primary tumors (∼80%) compared to recurrent cases (∼60%). Most patients had an overall survival of 6-36 months (∼ 70%), while a smaller proportion survived beyond 3 years.

**Figure 1.** Clinical and comparative proteomic profiling of glioblastoma plasma and tissue samples. (A) OncoPrint-style summary of clinical characteristics, including sex, age group (<40, 40-69, ≥70 years), diagnosis, recurrence, survival, resection extent, tumour location (hemisphere and lobe), and molecular markers (IDH1, MGMT, ATRX, TP53). (B) Principal component analysis (PCA) of plasma proteomes from healthy controls (*n* = 30), primary GBM (pGBM, *n* = 57), and recurrent GBM (reGBM, *n* = 47). PC1 and PC2 explain 11.3% and 9.39% of variance, respectively. (C) Principal component analysis (PCA) of tissue proteomes from primary GBM (pGBM, *n* = 125) and recurrent GBM (reGBM, *n* = 76). PC1 and PC2 explain 33.39% and 13.88% of variance, respectively. (D) Venn diagram showing overlap of proteins detected in tissue and plasma: 4,806 unique to tissue, 124 to plasma, and 205 shared. (E) Reactome pathway enrichment of proteins unique to tissue (blue, *n* = 97; see Methods), plasma (yellow), or shared (green). Bubble size reflects gene count; enrichment score from STRING.

#### Mapping the interface between glioblastoma tissue and circulating plasma proteome

.—Unlike tissues and cells, blood plasma lacks intrinsic protein synthesis. Instead, its proteomic composition is shaped by the dynamic exchange of proteins secreted, shed, or leaked from surrounding tissues and organs.^17^^ ,^^40^ In the context of GBM, the BBB poses a significant challenge by restricting the release of tumor-derived proteins into the circulation. To address this, we leveraged a cohort of matched tumor tissue and plasma samples from 55 GBM patients. This enabled us to construct a comprehensive proteomic landscape that captures both local (tumor-specific) and systemic (circulating) protein signatures. Our goal was to determine whether candidate biomarkers detected in plasma are actively secreted by tumor cells, or instead reflect passive release due to necrosis, apoptosis, or physiological turnover.

A total of 617 proteins were identified across all plasma samples, with 329 proteins (Figure 1A) passing quality control and retained for downstream analysis. Principal Component Analysis (PCA) demonstrated considerable consistency in the proteome from the healthy control, pGBM, and reGBM: the first 2 PCs (PC1 and PC2), which explained 11.3% and 9.39% of variance, did not differentiate the GBM and control samples (Figure 1B). In contrast, proteomic profiling of GBM tissue samples identified approximately 5,000 proteins, with PCA revealing distinct molecular signatures between pGBM and reGBM tumors (Figure 1C).

To dissect the biological relevance of these proteomic signatures, we performed pathway enrichment and protein-protein interaction (PPI) analyses on 3 groups (Figure 1D): proteins unique to plasma (*n* = 124), unique to tissue (*n* = 97 with variance > 2, see Methods), and those shared between compartments (*n* = 205). Although only ∼15% of tissue proteins overlapped with those detected in plasma, these shared proteins formed a complex network of highly interconnected proteins (198 nodes, 3,256 edges) with enrichment in coagulation, complement signaling, protein metabolism, Toll-like receptor cascade, RHO GTPase effector, RAS/RAF/MAPK cascade, immunity pathways and interleukin/angiotensin signaling (Figure 1E and Supplementary Figure S1A and B). Tissue-exclusive proteins formed a moderately connected network (91 nodes, 377 edges), enriched in pathways related to extracellular matrix remodeling, neurotransmitter release (serotonin, dopamine, glutamate), nervous system development, and receptor tyrosine kinase signaling (PDGF, MET, NCAM1; Figure 1E and Supplementary Figure S1C). These pathways underscore the tumor’s invasive and neurodevelopmental features. Plasma-exclusive proteins (72 nodes, 222 edges) were enriched in processes such as O-linked glycosylation—implicated in immune evasion and tumor-stroma interactions—and activation of complement components C4 and C2, central to classical and lectin pathway initiation (Figure 1E and Supplementary Figure S1D). Collectively, these data define a compartment-specific proteomic landscape of GBM, delineating distinct molecular programs in tissue and plasma while uncovering convergent pathways, such as coagulation, complement activation, and immune signaling.

#### Proteomic divergence between tumor tissue and plasma in primary and recurrent glioblastoma

.—To investigate the molecular evolution of GBM proteome, we analyzed matched primary and recurrent tumor samples from 27 patients, including both tissue and plasma specimens. This paired design enabled a direct comparison of proteomic dynamics across disease progression and biological compartments. Differential abundance analysis (DAA) revealed extensive proteomic remodeling in tumor tissue, with 299 proteins downregulated and 483 upregulated when comparing matched primary and recurrent tumors (Figure 2A and Supplementary Table S3A). Pathway enrichment analysis revealed significant alterations at recurrence (Figure 2B). These changes included marked suppression of cell cycle-related processes (eg, DNA strand elongation, RNA metabolism, G1/S transition) alongside a pronounced upregulation of neuronal signaling pathways (eg, neurotransmitter release, GPCR signaling) and mito­chondrial metabolic programs. Together, these findings underscore the emergence of distinct biological states in recurrent GBM, consistent with prior observations. In stark contrast to the dynamic alterations observed in the tissue proteome, the plasma proteome remained largely unchanged, showing high similarity between primary and recurrent GBM. Only 2 proteins (PI16 and HBG1) were significantly altered between primary and recurrent samples (Supplementary Table S3B), and no pathways were enriched. Next, we performed cellular deconvolution using the Microenvironment Cell Populations-counter (MCP-counter) ­algorithm.^41^ This approach, originally developed for bulk RNA-sequencing data, was adapted to estimate the abundance scores of immune and stromal cell types based on protein expression profiles. Cell type deconvolution analysis suggested a decrease in the frequency of B cells, dendritic cells (DC), monocytes and endothelial cells and an increase in neurons, astrocytes and OPCs in tissue at recurrence. In recurrent plasma samples, the analysis indicated modest changes in B cells, astrocytes and T cells (Figure 2C).

**Figure 2.** Matched plasma and tissue proteomic analysis in primary and recurrent glioblastoma. (A) Overlapping volcano plot of differentially abundant proteins in 27 matched primary GBM (pGBM)-recurrent GBM (reGBM) pairs in plasma (yellow) and tissue (blue). Thresholds: log_2_ fold change ≥ 0.5 and adjusted *P* < .05. (B) Dot plot of enriched reactome pathways from differentially abundant proteins in matched tissue samples. Bubble size reflects gene count; colour indicates adjusted p value; enrichment score derived from ReactomePA:gsePathway. (C) Dot plots of MCP-counter–inferred immune cell abundances from tissue and plasma proteomes. (D-E) Co-expression module dendrograms in pGBM and reGBM plasma (D) and tissue (E). The top panels show unmerged modules; the bottom panels show merged modules by eigengene similarity. (F) Correlation between module eigengenes and clinical variables: condition (recurrence/primary); sex (male/female); age; p53 status (wild type/mutant); MGMT status (unmethylated/methylated). (G) A TOM plot demonstration module independence.

Weighted Gene Co-expression Network Analysis (WGCNA)^39^ identified a single co-expression module in plasma, which remained stable during the transition to recurrence (Figure 2D). Consistent with previous results, coagulation and deregulation of hemostasis were among the top pathways enriched in this cluster (Supplementary Figure S2A and B and Supplementary Table S4A and B). In contrast, WGCNA on pGBM and reGBM tissue independently revealed 6 and 3 clusters, respectively (Figure 2E). To assess the dynamic changes occurring during disease progression, we applied the WGCNA on the matched pGBM/reGBM tissue cohort, detecting eight distinct modules of proteins interconnected by their co-expression patterns (Supplementary Figure S2C). Pathway enrichment analysis (Supplementary Figure S3) revealed discrete biological programs across these modules. The “pink module” was enriched for platelet degranulation, hemostasis, and erythrocyte gas exchange, suggesting a role in vascular interface and thrombo-inflammatory signaling. The “yellow module” featured pathways related to coagulation, immune response, and ­complement cascade regulation, while the “red module” showed enrichment for extracellular matrix remodeling, glycosaminoglycan metabolism and PDGF signaling. The “green module” was enriched for immuno-inflammatory signaling, including cytokine-mediated pathways and VEGF-VEGFR2, and positively correlated with male sex (*r* = 0.31; *P* = .02; Figure 2F). The “blue module” showed strong enrichment for SUMOylation, RNA metabolism and splicing, DNA repair, and cell cycle regulatory pathways. The “black module” was dominated by neutrophil degranulation, TLR4, and interleukin signaling. The “brown module” was enriched for protein biosynthesis, unfolded protein response, and selenocysteine synthesis. The “turquoise module” was associated with neurotransmitter/synaptic signaling, membrane trafficking and respiratory metabolism, and was positively correlated with recurrence (*r* = 0.32; *P* = .02; Figure 2F). In contrast, the blue (*r* = 0.35; *P* = .008), brown (*r* = 0.49; *P* = 1e-04), and black (*r* = 0.30; *P* = .03) modules were positively correlated with primary GBM, indicating their relevance to the initial tumor state (Figure 2F). A topological overlap matrix (TOM) plot showed that each module of this complex network was independent (Figure 2G). These results suggest that the systemic proteomic landscape of plasma is buffered against the extensive heterogeneity observed within the tumor microenvironment. Therefore, plasma proteome-based biomarkers might facilitate the detection of biologically distinct GBM subtypes.

### Systemic Pathway Alterations in the Plasma Proteome of Glioblastoma Patients

To identify biomarker candidates in the neat plasma of GBM patients, the plasma proteome of primary (pGBM) and recurrent (reGBM) GBM patients, as well as healthy individuals, was interrogated revealed a total of 150 differentially abundant (DA) proteins (*P* < 0.05; adjusted for age and gender) were identified between GBM (pGBM and reGBM; ALL_GBM) and healthy control groups; of these, 61 were increased and 89 decreased in abundance in ALL_GBM comparison of GBM versus healthy controls (*P* < .05; Supplementary Table S5A). After Benjamini-Hochberg (absolute log2 fold change (FC) of 0.5; adjusted *P* < .05), 2 up-regulated (HP; SAA1) and 22 down-regulated proteins remained significantly associated with GBM (Supplementary Table S5B). To elucidate systemic biological processes associated with GBM, we performed gene ontology (GO) enrichment analysis on plasma proteins differentially expressed in GBM patients relative to healthy controls (Figure 3A and B). This analysis revealed distinct enrichment patterns in upregulated and downregulated protein sets, reflecting coordinated alterations in immune, vascular, and structural pathways. Upregulated pathways in GBM plasma were predominantly associated with innate immunity, inflammation, and coagulation. The most significantly enriched processes included the acute inflammatory response (GO:0002526; false discovery rate (FDR) = 3.83 × 10^−12^), blood coagulation (GO:0007596; FDR = 4.06 × 10^−14^), and response to wounding (GO:0009611; FDR = 2.44 × 10^−11^). Complement activation (GO:0006958), zymogen activation (GO:0031638), and humoral immune responses (GO:0006959) were also significantly enriched, indicating a robust activation of both classical and alternative immune pathways. Additionally, processes related to regulation of proteolysis, response to stress, and positive regulation of signaling were overrepresented, suggesting systemic proteostatic and signaling adaptations reflected in the GBM plasma proteome. In contrast, down-regulated pathways were enriched for processes involved in cell adhesion (GO:0007155; FDR = 8.57 × 10^−8^), actin cytoskeleton organization (GO:0030036; FDR = 1.22 × 10^−5^), and wound healing (GO:0042060; FDR = 4.30 × 10^−9^). Notably, pathways critical for blood-brain barrier (BBB) maintenance (GO:0035633) and vascular development (GO:0001568, GO:0048514) were significantly suppressed, suggesting compromised vascular integrity and endothelial function. Additional down-regulated processes included negative regulation of cell death and muscle cell development, indicating broader disruptions in tissue homeostasis and structural remodeling. Together, these findings delineate a dual systemic signature in GBM plasma: a heightened inflammatory and pro-thrombotic state, coupled with suppression of vascular and structural maintenance pathways. This proteomic profile may reflect both tumor-driven systemic effects and potential biomarkers of disease progression. To further investigate potential factors which can aid the differentiation of GBM from the control group, we analyzed the PPI which revealed key molecules underpinning key deregulated pathways organized in a global network with a total of 49 edges between the 23 nodes (enrichment *P* < 1.0e-16; Figure 3C).

**Figure 3.** Glioblastoma-associated plasma proteome. (A and B) Dot plots of enriched Gene Ontology Biological Process terms for down-regulated (A) and up-regulated (B) proteins (*P* < .05). Bubble size reflects gene count; color indicates adjusted *P*-value; enrichment score derived from STRING. (C) Protein-protein interaction (PPI) network of 23 differentially abundant plasma proteins (STRING score ≥ 0.4). (D) Dot plots of inferred immune cell abundance from plasma proteomic profiles. (E) Chord diagram linking altered proteins, core pathway proteins to immune cell types. Up-regulated and down-regulated proteins (adjusted *P* < .05) are shown in pink and blue, respectively.

### Cellular Deconvolution Reveals Tumor-associated Shifts in Plasma-derived Cell Signatures

To identify the cellular origins of the altered plasma proteome in GBM, we employed the MCP-counter algorithm. Enrichment scores for 15 distinct populations revealed tumor-associated shifts in plasma-derived signatures, with ­astrocyte and oligodendrocyte progenitor cell (OPC)-associated proteins enriched in GBM plasma, consistent with central ­nervous system origin and glial protein release into ­circulation. In contrast, immune cell types such as B cells, T cells, mast cells, and radial glia appeared more abundant in control plasma, suggesting a relative depletion or suppression of these populations in the GBM-associated systemic environment (Figure 3D). Moreover, several of the cell-type-specific proteins were also identified as DA proteins and were enriched in GBM-associated pathways. Notably, SERPINA3, AGT, CLU, CST3, and SPARCL1, all associated with astrocytes, were upregulated in GBM plasma. Conversely, proteins marking immune and progenitor cell types: PDLIM1 (B cells), TUBA4A (T cells), and TUBA1B (radial glia) were downregulated in GBM plasma. These observations align with our pathway enrichment analysis, which suggests immune-inflammatory alterations, disruptions in coagulation and complement systems, and abnormalities in wound healing and blood vessel development pathways associated with GBM. Together, these findings highlight a tumor-associated reprogramming of the circulating proteome that reflects both local (astrocytic/OPC) and systemic (immune and vascular) cellular changes. This integrated proteomic pathway and deconvolution approach (Figure 3E) provides additional insight into the cellular contributors to the GBM plasma signature.

#### Selection of biomarkers for glioblastoma detection and monitoring

.—Finding the most informative feature(s) to detect GBM using plasma-based cell-free proteome was performed using the nested cross-validation (CV) with embedded feature selection (random forest).^42^ Our approach included an inner 10-fold CV for hyperparameter optimization. The classifier was then fitted on the outer training fold and tested on the left-out data. The 19 resulting classifiers were assessed using standard metrics such as area under the curve (AUC) and accuracy (Figure 4A-C). A 2-feature (F9&COMP) classifier showed the highest performance with AUC of 0.959 (95% CI: 0.925-0.993), accuracy of 90% (95% CI: 0.833, 0.95), specificity of 88% and sensitivity of 91%. Interestingly, the coagulation factor IX (F9) was among the up-regulated proteins shared between tissue and plasma, while cartilage oligomeric matrix protein (COMP) was down-regulated and uniquely detected in plasma, suggesting a compartment-specific biomarker combination. To test the robustness of this classifier fitted with F9 and COMP, a repeated cross-validation (*n* = 100) was performed and reported a mean AUC of 0.958 (95% CI: 0.957-0.958) and mean accuracy of 88.2% (95% CI: 0.935-0.808) (Figure 4D). The classifier performance on an unseen hold-out validation set showed AUC of 0.983 (CI: 0.9371-1), accuracy of 89% (CI: 0.669, 0.987), specificity of 75% and sensitivity of 93%. Intriguingly, increasing the number of features did not further improve the performance (Figure 4E-G) of classification, where F9 and COMP remained as top contributors. Of note, F9 and COMP had zero missing values, a fact that minimizes the risk of data leakage due to imputation, a common issue in proteomic data sets. Several highly ranked proteins, such as COMP, F9, CST3, HP, DCD, LUM, and SHBG, were also among the upregulated with significant adjusted p-values and absolute FC greater than 0.4. Although the absolute FC for F9, AMBP, SERPINA4, or SAA4 was not substantial, the difference in their abundance when comparing GBM patients to healthy controls was significant (adjusted *P* < 0.05). FGL1 was the only protein which did not show altered differential abundance in GBM compared to control (adjusted *P* > 0.05). Given the inevitability of recurrence in GBM,^43^^,^^44^ we investigated whether a classifier trained exclusively on plasma samples from patients with pGBM (AUC = 0.959, 95% CI: 0.9166-1.00; accuracy 92% (95% CI: 0.827-0.969); 87% specificity and 93% sensitivity could accurately detect recurrence. Remarkably, the F9-COMP classifier, trained on pGBM cases, achieved an AUC of 0.9733 and 100% specificity when applied to reGBM samples (82% accuracy with 95% CI: 0.6956-0.9048, 76% sensitivity and 100% specificity), demonstrating its robustness across disease stages (Figure 4H-I). This cross-stage generalizability (particularly notable given the extensive molecular divergence observed in tumor tissue between primary and recurrent GBM) suggests that the classifier captures stable, disease-intrinsic features of the GBM plasma proteome, possibly enabling early detection of recurrence without the need for retraining on recurrent cases. These findings highlight the potential of this dual-marker panel for real-time, noninvasive disease surveillance throughout the GBM clinical trajectory.

**Figure 4.** Machine learning-based discovery of biomarkers for glioblastoma detection. (A-C) Dot plots showing feature directionality and importance across outer folds of nested cross-validation for the best-performing model (A), model 9 (B), and model 19 (C). (D-F) Receiver operating characteristic (ROC) curves for GBM vs. healthy controls using the top discriminative features from the best-performing model (D), model 9 (E), and model 19 (F), with area under the curve (AUCs) of 0.96, 0.95, and 0.92, respectively. (G) Violin plots of abundance for 11 representative biomarkers across healthy controls (*n* =45), primary GBM (pGBM, *n* =57), and recurrent GBM (reGBM, *n* = 45). Pink squares denote median; Ns—nonsignificant; *P < .05; **P < .01; ***P < .001 and ****P < .0001 (pair wise Wilcoxon test). (H) ROC curve for distinguishing pGBM from healthy controls using F9 and COMP (AUC = 0.96; 95% CI: 0.92-1.00). (I) ROC curve for reGBM validation using the pGBM-trained model (AUC = 0.97; 95% CI: 0.94-1.00).

#### Validation of F9 and COMP as longitudinal biomarkers of GBM response to therapy

**Figure 5.**
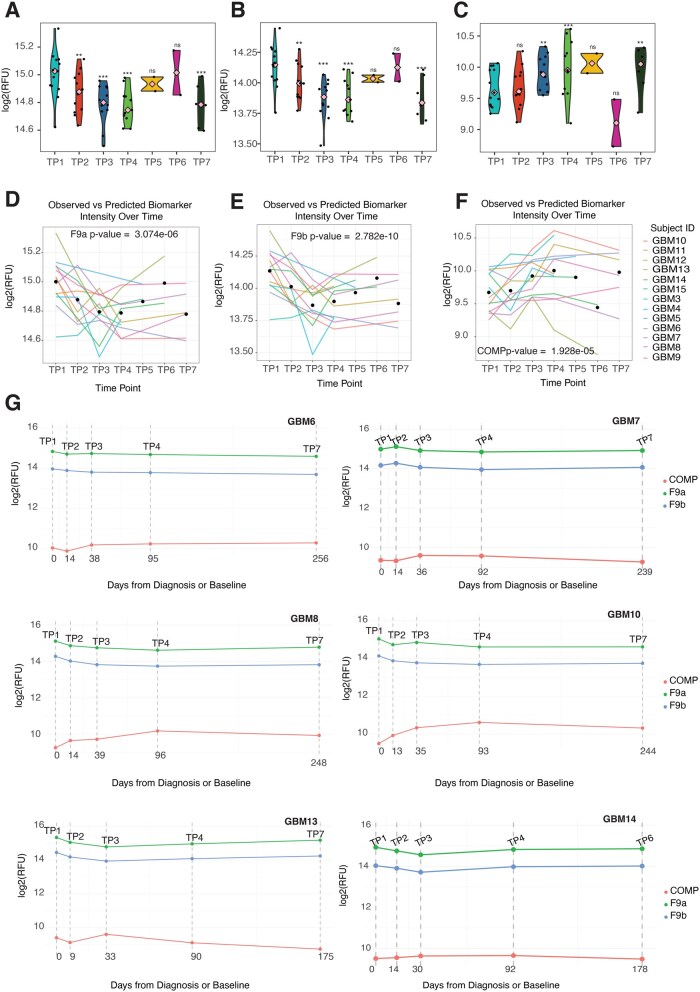
Longitudinal plasma profiling of F9 and COMP in glioblastoma (GBM) patients. (A-C) Violin plots showing the distribution of relative fluorescence units (RFUs) for F9a (A), F9b (B), and COMP (C) across all time points. Pink squares denote median; Ns, nonsignificant; **P < .01; ***P < .001 (Satterthwaite’s method for *t*-test). (D-F) Individual patient trajectories and model-based predictions for F9a (D), F9b (E), and COMP (F) derived from mixed-effects linear modeling. Wald chi-square tests verified the variation in RFU over time (*P* < .05) across the 3 models (G) Representative longitudinal profiles illustrating distinct biomarker dynamics and potential associations with treatment response or disease progression. TP1= pre-surgery; TP2 = post-surgery before oncological treatment; TP3 = mid-radio(RT)-chemotherapy with Temozolomide (day 15 of RT/TMZ); TP4 = post-adjuvant temozolomide (TMZ) course 1; TP5-TP7 = post-adjuvant TMZ courses 2, 4, and 6, respectively.

.—To evaluate the potential of F9 (measured using 2 independent aptamers F9a: seq.4876.32, F9b: seq.5307.12) and COMP (measured using a single aptamer seq.8043.153) biomarkers for monitoring GBM progression and treatment response, we profiled longitudinal samples from a cohort of 13 GBM patients (Supplementary Table S6A), yielding a total of 58 plasma samples collected across up to 7 standardized clinical time points. These included: presurgery (TP1; *n* = 13), postsurgery before oncological treatment (TP2; *n* = 12), mid-radio (RT)-chemotherapy with Temozolomide (TMZ) (day 15 of RT/TMZ, TP3; *n* = 12), after the first course of adjuvant temozolomide (TP4; *n* = 11), and subsequent adjuvant cycles (TP5 with *n* = 2; TP6 with *n* = 2; TP7 with *n* = 6). The median follow-up duration was 413 days (range: 230-536 days). Significant reductions in F9 relative fluorescence units (RFUs; Figure 5A and B and Supplementary Table S6B) were observed from TP1 to TP2 (β_2F9a_ = −0.12; β_2F9b_ = −0.12), TP3 (β_3F9a_ = −0.21; β_3F9b_ = −0.27), TP4 (β_4F9a_ = −0.21; β_4F9b_ = −0.24), and TP7 (β_7F9a_ = −0.22; β_7F9b_ = −0.25). In contrast, COMP levels (Figure 5C) increased steadily across treatment time points, with significant increases from TP1 to TP3 (β_3COMP_ = 0.25), TP4 (β_4COMP_ = 0.33), and TP7 (β_7COMP_ = 0.31). Linear mixed-effects modeling (Figure 5D-F; Supplementary Figure S5 and Supplementary Table S6C-E) revealed distinct and opposing temporal trajectories for F9 and COMP, underscoring their dynamic tumor behavior in response to therapy. Model-based predictions and ANOVA identified ‘time point’ as a significant fixed effect for both biomarkers, indicating that their levels changed meaningfully over the course of the standard-of-care treatment. To further illustrate their clinical relevance, representative patient-level profiles (for those with complete 5 time points) are shown in Figure 5G). Together, these findings highlight the potential of F9 and COMP as noninvasive, longitudinal biomarkers for GBM monitoring. Their opposing dynamics and consistent trends across patients emphasize their potential to complement standard imaging. Overall, our findings highlight the importance of clinical validation for our candidate biomarkers, as they might open a door for earlier and more precise insights into treatment response and disease progression.

## Discussion

Proteomics is rapidly transforming the landscape of noninvasive cancer diagnostics, offering a systems-level perspective that integrates tumor biology with host response. It uniquely captures both tumor-secreted proteins and systemic physiological changes such as inflammation, immune modulation, and coagulation. This contrasts with ctDNA/cell-free DNA, and methylation-based assays,^10^^,^^11^^,^^45^ which are often constrained by low analyte abundance, particularly in central nervous system tumors where the BBB limits the release of tumor-derived nucleic acids into circulation.^5^ Our study demonstrates that plasma proteomics offers a robust platform for GBM biomarker discovery, capturing both tumor-secreted proteins and systemic physiological responses, such as immune-inflammation and coagulation. Several studies have investigated the plasma proteome of GBM patients using MS (either label-free or TMT-labelled MS), but methodological and design limitations remain. We selected the TMT-labelled MS approach for neat plasma analysis due to its ability to multiplex multiple samples in a single run, minimizing batch effects and enhancing quantification precision, critical for detecting subtle proteomic changes in complex matrices like plasma. This method also improves sensitivity for low-abundance proteins, making it well-suited for biomarker discovery.^46^ Our plasma proteomic analysis reveals a distinct systemic signature in GBM patients, characterized by the upregulation of inflammatory and coagulation pathways and the downregulation of vascular and structural maintenance processes. The downregulation of pathways involved in cell adhesion, actin cytoskeleton organization, and BBB maintenance suggests systemic impairment of vascular integrity and tissue remodeling. These alterations may reflect tumor-induced endothelial dysfunction or the shedding of structural proteins from compromised vasculature. The suppression of wound healing and vascular morphogenesis pathways further supports the notion of disrupted tissue repair mechanisms in GBM patients.

The pronounced enrichment of inflammatory pathways including acute inflammatory response, complement activation, and humoral immune response aligns with previous reports of systemic immune dysregulation in GBM. Elevated levels of acute-phase proteins and complement components in plasma may reflect both tumor-driven inflammation and a broader host response to malignancy. Notably, the upregulation of coagulation-related pathways, including fibrin clot formation and zymogen activation, supports the concept of a hypercoagulable state in GBM, which has been associated with increased risk of thromboembolic events and poor prognosis. Boonyewan et al.^22^ reported increased platelet count after chemo-irradiation as predictive of GBM patient survival. Further supporting this, Mandoj et al.^47^ reviewed the biological basis of hypercoagulability in brain tumors, particularly GBM, and described how oncogenic mutations (eg, EGFRvIII, PTEN loss) drive tissue factor expression and thrombin generation, promoting tumor progression and angiogenesis. They highlighted the concept of tumor-specific “coagulomes,” where molecular subtypes of GBM exhibit distinct prothrombotic profiles. To infer the cellular origins of altered plasma proteins in GBM, we adapted and applied MCP-counter to proteomic data,^41^ revealing enrichment of astrocyte-associated proteins in GBM and depletion of immune cell markers compared to controls. Intriguingly, our data indicate increased relative frequency of neurons and reduced number of immune cells at recurrence. This is in contrast with published studies employing scRNA-sequencing data. Thus, this approach has limitations as the MCP-counter analysis using bulk proteome data may underrepresent immune populations due to protein abundance bias. These findings should therefore be interpreted as indicative, warranting validation through orthogonal single-cell or spatial approaches.

Importantly, our and other studies converge on key biological themes—coagulation, immune modulation, and extracellular matrix dynamics underscoring the relevance of these systemic processes in GBM pathophysiology and their utility for noninvasive biomarker development.^21^^,^^25^^,^^36^ Unlike Liu et al., who focused on mechanistic insights using a nanoparticle-enabled pipeline in a small patient set (*n* = 10) and murine models (GL261), and Clavreul et al., who emphasized prognostic serum markers without longitudinal assessment, our work integrates discovery-driven proteomics with machine learning and network analysis in a large, clinically annotated cohort. By comparing systemic and tumor proteomes, validating a dual-marker classifier, and demonstrating longitudinal biomarker dynamics, this study advances beyond prior efforts to deliver a clinically actionable approach for GBM surveillance.

Our identification of F9 and COMP as top-performing plasma biomarkers—both implicated in coagulation and extracellular matrix remodeling—highlights a plausible mechanistic link between tumor biology and systemic haemostatic alterations. Recognizing the potential concern that these findings could represent artefacts of plasma contamination, we performed a rigorous cross-check against the list of common plasma contaminants reported by Geyer et al. Neither F9 nor COMP was associated with contamination from platelets, erythrocytes, or coagulation-related artefacts, supporting their biological relevance. In contrast, 33.3% of proteins prioritized by Liu et al., were classified as common contaminants, including markers from erythrocytes (*n* = 3), platelets (*n* = 8), coagulation pathways (*n* = 4), or shared among panels (*n* = 1). These comparisons underscore the importance of implementing contamination-aware workflows in plasma proteomics to ensure biomarker specificity and reproducibility.

While F9 up-regulation in GBM plasma and tumor tissue may reflect the tumor’s pro-thrombotic and inflammatory microenvironment, COMP, markedly down-regulated in plasma (and not detected in tumor tissue), likely mirrors the absence of COMP-producing stromal cells and the proteolytic degradation of extracellular matrix components within the immunosuppressive tumor niche. These opposing trends not only likely enhance the specificity of a dual-marker panel but also provide mechanistic insight into GBM pathophysiology. The validation of F9 and COMP using aptamer-based profiling in a longitudinal cohort provides compelling evidence for their utility as dynamic, noninvasive biomarkers of treatment response. In contrast to their static diagnostic role, these markers exhibited distinct temporal trajectories: F9 was elevated at diagnosis and declined following surgery and chemoradiotherapy; conversely, COMP was suppressed at baseline and increased during treatment. These opposing trends not only enhance the specificity of the F9-COMP biomarker pair but also mirror the dual systemic signature observed in our untargeted proteomic analyses.

Our findings uncover a striking contrast between the tumor and systemic plasma proteomes in GBM. While tumor tissue demonstrates profound heterogeneity and dynamic subpopulation shifts at recurrence, the plasma proteome remains remarkably stable. The minimal changes observed in plasma between primary and recurrent GBM likely reflect systemic buffering mechanisms that conceal tumor evolution. This phenomenon has important clinical implications: if circulating protein profiles remain largely unaffected by intracranial molecular reprogramming, plasma-based assays may lack the sensitivity required for early detection of recurrence. Such limitations could delay therapeutic intervention and compromise patient outcomes. To improve diagnostic specificity, comparative profiling across GBM, lower-grade gliomas, and brain metastases is warranted. Future studies should also assess whether integrating plasma biomarker analysis with advanced imaging modalities enhances the diagnostic performance of candidate proteins such as F9 and COMP.

## Supplementary Material

vdag015_Supplementary_Data

## Data Availability

In line with GDPR and Danish law, only fully anonymized datasets are publicly available. Personally identifiable information has been removed or anonymized. Raw data and metadata will be accessible to qualified researchers upon publication, under appropriate data and material transfer agreements.
