# Supplementary material for: Noninvasive detection and monitoring of glioblastoma subtypes via dual-marker plasma proteomics
Source: Neurooncol Adv. 2026 Feb 5;8(1):vdag015. doi: 10.1093/noajnl/vdag015 (PMC13012891; doi:10.1093/noajnl/vdag015)
Supplement: vdag015_Supplementary_Data [file vdag015_supplementary_data.docx]

## **SUPPLEMENTARY DATA**

## **Supplementary Methods**

## **Sample Preparation for TMT-MS**

In brief, 10 µL of plasma was lysed using 90 µL lysis buffer and boiled at 95 °C for 5 min. Subsequently, samples were sonicated at 4 °C for 5 x 30 s in a BioRuptor sonication water bath (Diagnode). Protein concentrations were measured using Pierce Rapid Gold BCA Assay (Thermo Fisher Scientific) and 25 µg protein was used for digestion. Samples were diluted 1:3 in digestion buffer and digested with 250 ng LysC at 37 °C for 1 h with agitation. Samples were diluted further to 1:10 using digested buffer and digested overnight with 250 ng trypsin at 37 °C with agitation. Enzyme activity was quenched using 2 % trifluoroacetic acid (TFA) to a final concentration of 1 %. Samples were then desalted using SOLAµ solid phase extraction plates (Thermo Fisher Scientific). The plates were activated using 200 µL methanol, then 200 µL buffer B and equilibrated 2x with 200 µL buffer A’. Subsequently, samples were loaded onto the plates by centrifugation at 1,500 rpm for 1 min, washed 2x with 200 µL buffer A and eluted 2x with 30 µL buffer B’ into clean PCR plates. The eluted peptides were concentrated using an Eppendorf centrifugal vacuum concentrator at 60 °C and reconstituted in 20 µL 50 mM 4-(2-hydroxyethyl)-1-piperazine ethane sulfonic acid (HEPES) for tandem mass tag (TMT) labelling. Protein concentrations were determined using a Nanodrop, and 10 µg peptide, in a total of 22 µL, was used for the labelling. From each sample, 2 µL was transferred to a new tube to make a normalisation mix. All samples including the normalisation mix were labelled according to the manufacturer’ instruction, then mixed 1:1:1:1:1:1:1:1:1:1:1:1:1:1:1:1 (16-plex) and diluted with appropriate amount of 2 % TFA to bring percentage of acetonitrile below 5 %. Subsequently, the samples were cleaned up using SOLAµ solid phase extraction plates and concentrated as described above. Before mass spectrometry (MS) analysis, the samples were resuspended in 16 µL high pH buffer A and 30 µg peptide was fractionated using an offline Ultimate 3000 liquid chromatography system (Thermo Fisher Scientific) at 5 µL/min flowrate. The peptides were separated on a 120 min gradient (5 % to 35 % acetonitrile), while fractions were collected every 120 sec. The 60 fractions were pooled into 30 final fractions, acidified to pH < 2 using 1 % TFA and loaded onto Evotips (Evosep) according to the manufacturer’s instructions.

## **MS Data Acquisition**

Peptides were eluted over a 44 min gradient and analysed on an Orbitrap eclipse tribrid mass spectrometer (Thermo Fisher Scientific) running in a SPS-MS3 top speed method. Full MS spectra were acquired at 120,000 resolution, with an automated gain control (AGC) target of 2 × 10^5^, maximum injection time of 50 ms and a scan range of 375–1500 m/z. The MS2 spectra were obtained in the linear ion trap operating at rapid speed, with an AGC target value of 1 × 10^4^, maximum injection time of 50 ms, a normalised collision-induced dissociation (CID) collision energy of 35 and an intensity threshold of 5e3. MS2 spectra were sent on-the-fly to the real time search (RTS) module^1^, where they were searched against the homo sapiens UniProt database in real-time. Modifications for the RTS algorithm were Methionine oxidation (variable), Cysteine carbamidomethyl (static), and TMTPro on K residues and peptide N-termini (static). Only those MS2 scans that resulted in a peptide identification were subjected to SPS-MS3 analysis. For these, 5 precursor fragments were selected for SPS-MS3 analysis, fragmented with a normalised higher-energy CID collision energy of 63, and ions collected for a maximum of 86 ms or AGC target of 100,000. MS3 spectra were collected at 50,000 resolution and a scan range of 100-500 for reporter ion quantification. Dynamic exclusion was set to 60 sec, and ions with a charge state < 2, > 7 or unknown were excluded. MS performance was verified for consistency by running complex cell lysate quality control standards, and chromatography was monitored to check for reproducibility. The raw files were analysed using Proteome Discoverer (version 2.4). TMT SPS-MS3 quantitation was enabled in the processing and consensus steps, and spectra were matched against the 9606 Human databases obtained from UniProt. Dynamic modifications were set as Oxidation (M), Deamidation (N, Q), and Acetyl on protein N-termini. Cysteine carbamidomethyl and TMT 16-plex were set as static modifications. All results were filtered to an 1 % FDR, and protein quantitation was done using the built-in Minora Feature Detector with statistical significance testing done with the built-in t-test.

**MS Data Processing**

In Proteome Discoverer, the abundances were scaled by setting the scaling mode parameter to “on all average”, which resulted in all batch-specific TMT normalisation mixes were set to 100, and all other samples in the same batch adjusted up or down relative to 100.

**Data filtering and quality control**

Proteins identified with more than two peptides and fewer than 30% of missing values across all samples were retained. The correlation of the duplicated samples was assessed using limma::duplicateCorrelation^2^, due to a high consensus correlation (0.887) duplicated samples were removed at random. Missing values were imputed using the function impute::impute.knn() (version 1.80.0), averaging the corresponding values from the k-nearest neighbours. The resulting proteome numbering of 329 in the plasma samples (n = 132) and 5,011 in tissue samples (n =201) was used for downstream analysis.

## **Differential Abundance Analysis (DAA)**

The raw values were log2 transformed, and DAA was conducted using the limma package (version 3.62.2)^2^. Limma employs an empirical Bayes method to moderate the standard error of the estimated log2 fold changes (FC). DA features were classified as up-regulated, if they exhibited a FC > 0.5 with an adjusted p value < 0.05 or down-regulated, if they showed a FC < -0.5, with an adjusted p-value < 0.05. All the models were adjusted for gender and age group. When DAA was conducted on paired primary-recurrence samples, the function limma::duplicateCorrelation() was utilised to estimate the correlation between the matched pairs. Subsequently, the ‘block’ argument was incorporated into the limma:: lmFit() function to assess a paired test accurately.

## **Gene Set Enrichment Analysis and Protein- Protein Interaction**

Functional enrichment annotation of Gene Ontology Biological Process, Reactome pathway, and PPI (default settings, interaction score = 0.4) analyses were conducted utilising STRING database (<https://string-db.org/>)^3^, clusterProfiler (version 4.14.6)^4^ and ReactomePA: gsePathway (version 1.50.0)^5^. For functional enrichment annotation, the most relevant and non-redundant pathways were retained.

**Correlation analysis of plasma and tissue proteome**

To ensure protein abundances are fully comparable across plasma and tissue samples, and to reduce systemic batch effects between measurement modalities before downstream analyses, preprocessCore::normalize.quantiles() (version 1.68.0) was applied to force all samples from both tissue and plasma to share the same overall intensity abundance distribution. The correlations between each plasma protein and the corresponding in tissue were calculated using the WGCNA::corAndPvalue() function with the Pearson method, and the resulting p-values were adjusted. Proteins with adjusted p-value < 0.05 were considered moderate and statistically significantly correlated in tissue‐to‐plasma.

**Bioinformatic software**

Data analysis was conducted using the R statistical software package (version 4.4.2), RStudio (version 2024.12.0+46). For data visualisation different R libraries were used: ggplot2 (version 3.5.2), circlice() (version 0.4.16), ggalluvial() (version 0.12.5) complexHeatmap() (version 2.22.0), ggpubr() (version 0.6.0). The in-house script and datasets used for the analysis and figures generation are available via GitHub, https://github.com/Brain-Tumor-Biology/Integrative-Tumour-Plasma-Proteomics-Project.

**Supplementary References**

1 Schweppe, D. K. *et al.* Full-Featured, Real-Time Database Searching Platform Enables Fast and Accurate Multiplexed Quantitative Proteomics. *Journal of Proteome Research* **19**, 2026-2034 (2020). <https://doi.org:10.1021/acs.jproteome.9b00860>

2 Ritchie, M. E. *et al.* limma powers differential expression analyses for RNA-sequencing and microarray studies. *Nucleic Acids Res* **43**, e47 (2015). <https://doi.org:10.1093/nar/gkv007>

3 Szklarczyk, D. *et al.* The STRING database in 2023: protein-protein association networks and functional enrichment analyses for any sequenced genome of interest. *Nucleic Acids Res* **51**, D638-D646 (2023). <https://doi.org:10.1093/nar/gkac1000>

4 Yu, G., Wang, L. G., Han, Y. & He, Q. Y. clusterProfiler: an R package for comparing biological themes among gene clusters. *OMICS* **16**, 284-287 (2012). <https://doi.org:10.1089/omi.2011.0118>

5 Yu, G. & He, Q. Y. ReactomePA: an R/Bioconductor package for reactome pathway analysis and visualization. *Mol Biosyst* **12**, 477-479 (2016). <https://doi.org:10.1039/c5mb00663e>

## **Supplementary Figures and legends**

## **
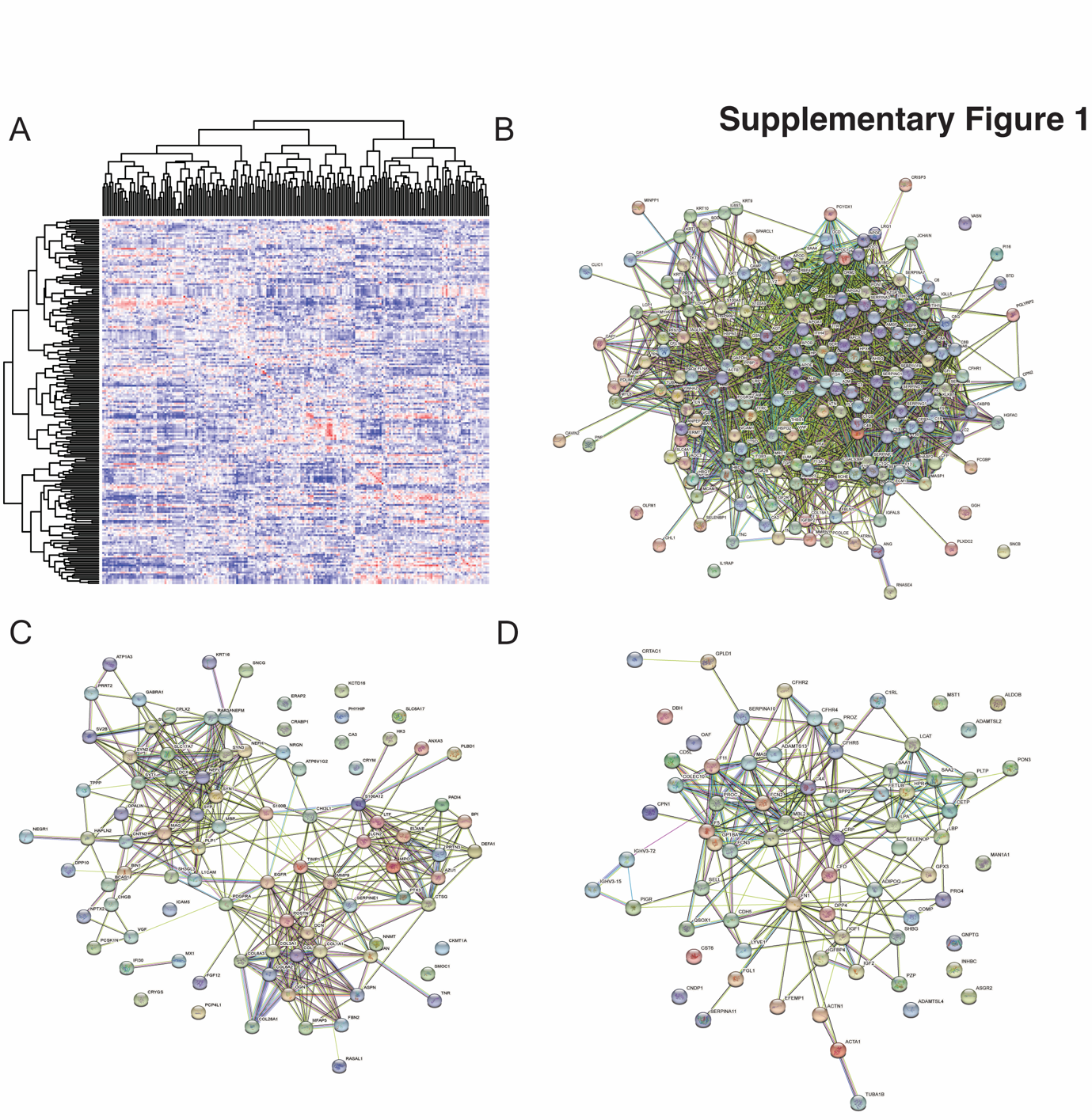
**

**Supplementary Figure 1. Correlation and protein-protein interaction of tissue and plasma proteins.**

**(A**) Heatmap showing Pearson correlation coefficients between log₂-transformed protein abundances in matched tissue and plasma samples. The colour scale indicates correlation strength, from negative (blue) to positive (red).

(**B-D**) PPI networks of proteins shared between tissue and plasma (198 nodes, 3,256 edges; **B**), unique to tissue (91 nodes, 377 edges; **C**)and unique to plasma (72 nodes, 222 edges; **D**) (STRING score ≥ 0.4).

**Supplementary Figure 2. Supplementary information supporting Figure 2.**

(**A-B**) Dot plot of enriched reactome pathways from single module identified in the plasma proteome of primary (**A**) and recurrent (**B**) GBM patients. Bubble size reflects gene count; colour indicates adjusted p-value; enrichment score derived from STRING.
(**C**) WGCNA analysis of GBM (primary and recurrent) tissue proteomes supporting Figure 2F-G. The top panel shows unmerged modules; the bottom panels show merged modules by eigengene similarity.


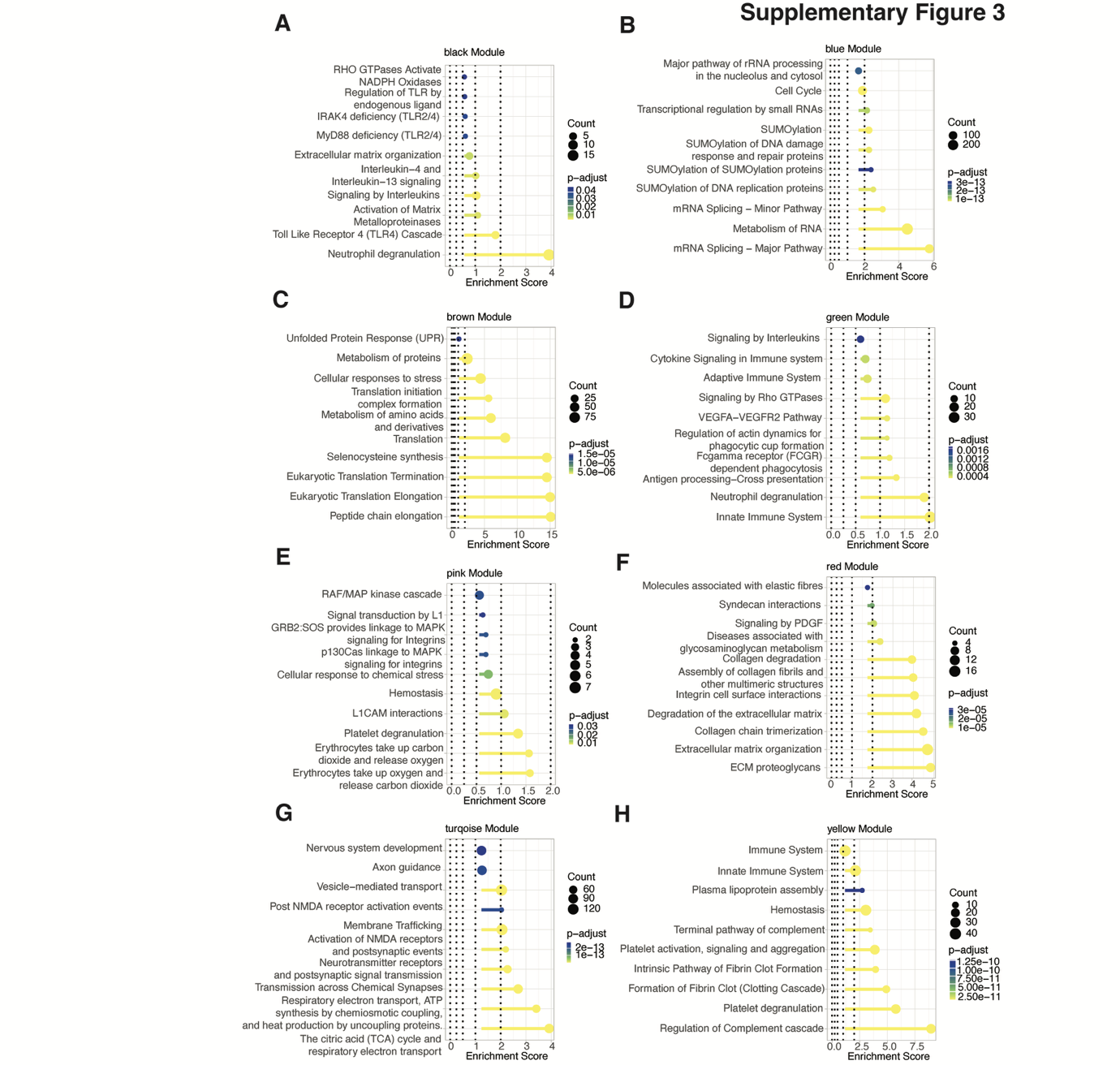


**Supplementary Figure 3. Enriched reactome pathways of the proteins for WGCNA modules.**

(**A-H**) Shows enriched terms from blue, brown, red, pink, turquoise, yellow, green, and black modules identified by WGCNA analysis of primary and recurrent GBM tissue proteome.

**
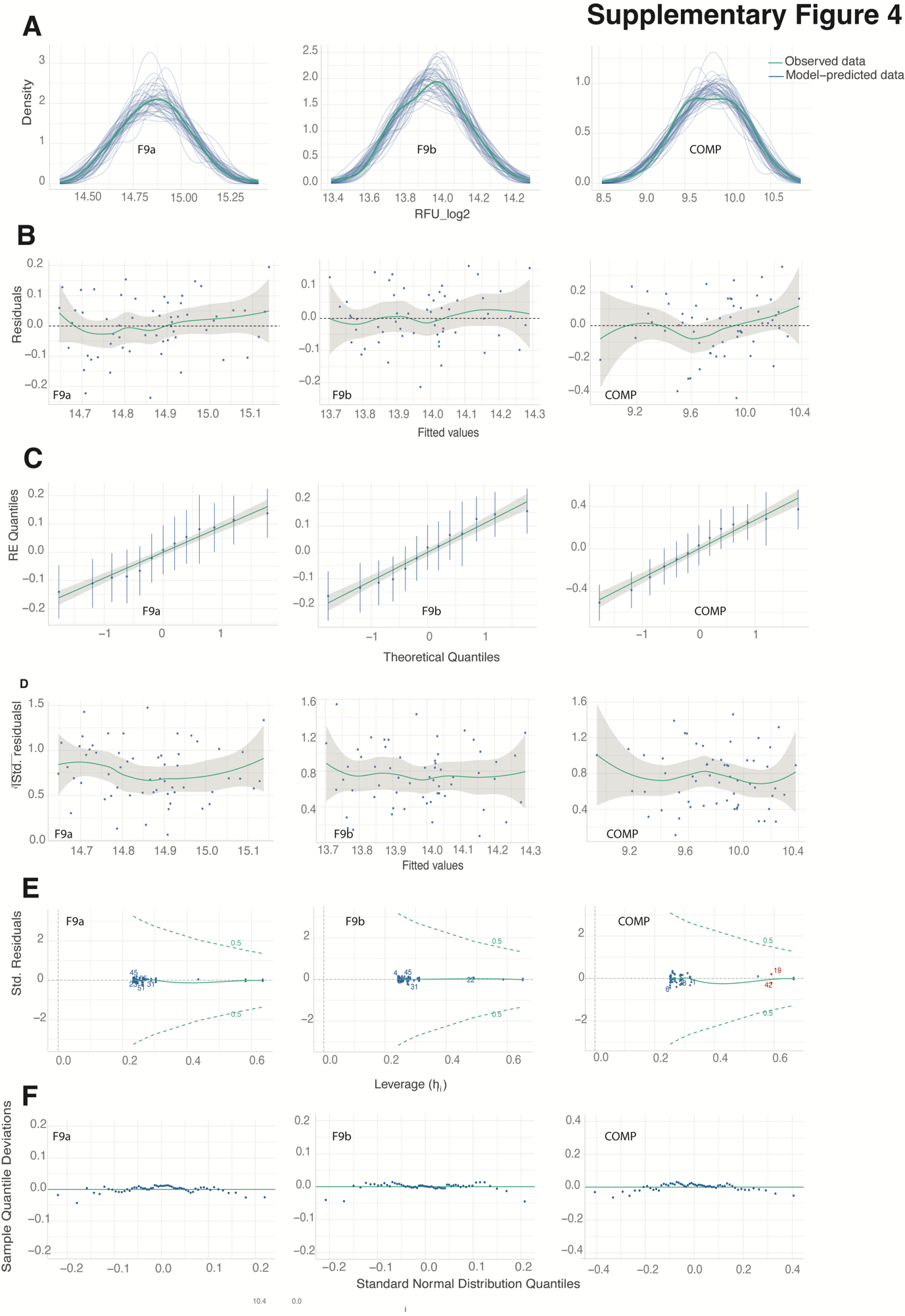
**

**Supplementary Figure 4.** **Model diagnostic plot supporting Figure 5D-F.**

(**A-F**) Visual assessment of posterior predictive checks (**A**) linearity (**B**), homogeneity of variance (**C**), influential observations (**D**), and the normality of residuals (**E**) and random effects (**F**) for F9a, F9b, and COMP.

**Supplementary Tables**

**Supplementary Table 1. Tissue cohort - histopathological characteristics**

|  | Primary  N = 125^1^ | Recurrent  N = 76^1^ |
| --- | --- | --- |
| Variable |  |  |
| Sex |  |  |
| Female | 51 (41%) | 35 (46%) |
| Male | 74 (59%) | 41 (54%) |
| Age Group |  |  |
| > 80 | 13 (10%) | 1 (1.3%) |
| 20-29 | 0 (0%) | 3 (4.0%) |
| 30-39 | 2 (1.6%) | 4 (5.3%) |
| 40-59 | 45 (36%) | 35 (47%) |
| 60-79 | 65 (52%) | 32 (43%) |
| Diagnosis |  |  |
| Glioblastoma (grade 4) | 119 (98%) | 66 (99%) |
| Gliosarcoma (grade 4) | 3 (2.5%) | 1 (1.5%) |
| Overall survival |  |  |
| 0-6 months | 23 (18%) | 1 (1.3%) |
| 36-240 months | 23 (18%) | 26 (34%) |
| 6-36 months | 79 (63%) | 49 (64%) |
| Resection |  |  |
| Complete | 97 (78%) | 47 (62%) |
| Partial | 28 (22%) | 29 (38%) |
| Hemisphere |  |  |
| Left | 52 (42%) | 36 (47%) |
| Right | 73 (58%) | 40 (53%) |
| Lobe location |  |  |
| Frontal | 41 (33%) | 29 (38%) |
| Frontotemporal | 0 (0%) | 1 (1.3%) |
| Occipital | 17 (14%) | 8 (11%) |
| Parietal | 22 (18%) | 7 (9.2%) |
| Temporal | 43 (34%) | 30 (39%) |
| Temporooccipital | 1 (0.8%) | 1 (1.3%) |
| Thalamus | 1 (0.8%) | 0 (0%) |

^1^n (%)

**Supplementary Table 2. Plasma cohort - histopathological characteristics**

|  | Control  N =30^1^ | Primary  N = 57^1^ | Recurrent  N = 45^1^ |
| --- | --- | --- | --- |
| Variable |  |  |  |
| Sex |  |  |  |
| Female | 17 (57%) | 25 (44%) | 21 (47%) |
| Male | 13 (43%) | 32 (56%) | 24 (53%) |
| Age Group |  |  |  |
| > 80 | 1 (3.3%) | 3 (5.3%) | 1 (2.2%) |
| 20-2 | 3 (10%) | 0 (0%) | 0 (0%) |
| 30-39 | 1 (3.3%) | 1 (1.8%) | 0 (0%) |
| 40-59 | 10 (33%) | 25 (44%) | 22 (49%) |
| 60-79 | 15 (50%) | 28 (49%) | 22 (49%) |
| Diagnosis |  |  |  |
| Glioblastoma (grade 4) |  | 54 (95%) | 44 (98%) |
| Gliosarcoma (grade 4) |  | 3 (5.3%) | 1 (2.2%) |
| Overall survival |  |  |  |
| 0-6 months |  | 6 (11%) | 0 (0%) |
| 36-240 months |  | 11 (19%) | 9 (20%) |
| 6-36 months |  | 40 (70%) | 36 (80%) |
| Resection |  |  |  |
| Complete |  | 46 (81%) | 29 (64%) |
| Partial |  | 11 (19%) | 16 (36%) |
| Hemisphere |  |  |  |
| Left |  | 25 (44%) | 25 (56%) |
| Right |  | 32 (56%) | 20 (44%) |
| Lobe location |  |  |  |
| Frontal |  | 19 (33%) | 17 (38%) |
| Occipital |  | 9 (16%) | 6 (13%) |
| Parietal |  | 8 (14%) | 5 (11%) |
| Temporal |  | 20 (35%) | 16 (36%) |
| Temporooccipital |  | 1 (1.8%) | 1 (2.2%) |

^1^n (%)

| Supplementary Table 3A. Differential abundant tissue proteins in pGBM vs reGBM (adjusted p value < 0.05 & absolute FC < 0.05) | | | | | | |
| --- | --- | --- | --- | --- | --- | --- |
| Protein ID | **logFC** | **AveExpr** | **t** | **P.Value** | **adj.P.Val** | **B** |
| MPO | 1.39099413 | 5.71211886 | 2.85386922 | 0.0063931 | 0.03302662 | -2.4946112 |
| IGF2BP3 | 1.3813206 | 5.29681626 | 3.90656214 | 0.00029706 | 0.00759461 | 0.25902582 |
| SPP1.iso5 | 1.37599896 | 6.13548681 | 4.80109334 | 1.6272E-05 | 0.00291583 | 2.91575929 |
| PADI4 | 1.36831441 | 5.74729961 | 3.6212287 | 0.00071362 | 0.01131627 | -0.5356544 |
| PLBD1 | 1.26379344 | 6.53289101 | 2.9172657 | 0.00538947 | 0.03010776 | -2.3441605 |
| CSPG4 | 1.23977647 | 6.23970479 | 5.40042461 | 2.1188E-06 | 0.00263481 | 4.79013832 |
| ANGPT2 | 1.21539634 | 6.26517507 | 5.28457791 | 3.1548E-06 | 0.00263481 | 4.42392159 |
| ELANE | 1.2088065 | 5.92809074 | 2.64172306 | 0.0111498 | 0.04594338 | -2.9811027 |
| ENPEP | 1.17495285 | 6.36852302 | 4.41264963 | 5.8895E-05 | 0.0044048 | 1.7360423 |
| DCHS1 | 1.16862985 | 6.38157439 | 5.45590826 | 1.7501E-06 | 0.00263481 | 4.96605547 |
| S100A12 | 1.16622797 | 5.5550311 | 2.76482224 | 0.00809778 | 0.03780265 | -2.7020547 |
| DEFA1 | 1.11931473 | 5.05102893 | 2.87083741 | 0.00610869 | 0.03228971 | -2.454565 |
| GUSB | 1.10552547 | 6.55043695 | 3.78898325 | 0.00042776 | 0.00895549 | -0.0721632 |
| CHI3L1 | 1.08396951 | 6.39211654 | 2.69214893 | 0.00979034 | 0.04262328 | -2.8678996 |
| MCM3 | 1.0641993 | 5.85791409 | 4.25700761 | 9.7624E-05 | 0.00509577 | 1.27369796 |
| LCN2 | 1.05417509 | 5.92122663 | 2.84939026 | 0.0064702 | 0.03328764 | -2.5051545 |
| LTF | 1.05339653 | 5.68046219 | 2.61214618 | 0.0120256 | 0.04801761 | -3.0467745 |
| CRISP3 | 1.04636983 | 6.22211631 | 2.97046777 | 0.00466262 | 0.02791443 | -2.2161755 |
| TNC | 1.03488837 | 6.30085842 | 3.59303101 | 0.00077693 | 0.0115869 | -0.612462 |
| MCM6 | 1.01279406 | 5.93533347 | 4.04253267 | 0.00019375 | 0.00633361 | 0.64804712 |
| HMGB2 | 1.00544199 | 6.21251087 | 4.98451874 | 8.7716E-06 | 0.00291583 | 3.48354013 |
| MCM2 | 0.98920421 | 5.99982871 | 4.41604746 | 5.8245E-05 | 0.0044048 | 1.74620775 |
| NDC80 | 0.98584711 | 6.04564576 | 4.52597079 | 4.0609E-05 | 0.00391327 | 2.0766318 |
| NASP | 0.98499628 | 6.08689338 | 4.64713847 | 2.7199E-05 | 0.00363986 | 2.44417952 |
| TOP2A | 0.98217 | 5.58322303 | 3.30922679 | 0.00179587 | 0.01720672 | -1.3664369 |
| SMC2 | 0.9802618 | 6.16707759 | 4.75721423 | 1.8847E-05 | 0.0030333 | 2.78086144 |
| PCNA | 0.97953058 | 6.05825445 | 4.52810333 | 4.0324E-05 | 0.00391327 | 2.08307128 |
| RNASEH2B | 0.97288416 | 6.25699407 | 6.32265975 | 8.5528E-08 | 0.00042858 | 7.74130039 |
| MCM4 | 0.97201253 | 5.95756273 | 4.16137537 | 0.00013274 | 0.00576674 | 0.9929989 |
| IL1RAP | 0.96749183 | 6.27380888 | 3.74506338 | 0.00048957 | 0.00929258 | -0.1945651 |
| BCHE | 0.96685376 | 6.36172209 | 4.38845262 | 6.3735E-05 | 0.00456851 | 1.66373738 |
| PLOD3 | 0.96249487 | 6.075252 | 3.48124711 | 0.00108495 | 0.01305238 | -0.9136712 |
| NES | 0.95856927 | 6.02695148 | 3.17237147 | 0.00265729 | 0.02133584 | -1.7167146 |
| IGFBP2 | 0.95556447 | 6.1707039 | 3.71786614 | 0.00053207 | 0.00994846 | -0.2699931 |
| SMC4 | 0.94627118 | 6.10488126 | 4.32440109 | 7.8498E-05 | 0.00479699 | 1.47308633 |
| IGF2BP2 | 0.9381369 | 5.92209282 | 3.06646632 | 0.00357747 | 0.02449002 | -1.9813588 |
| FKBP10 | 0.92720381 | 6.24144629 | 3.40035913 | 0.00137719 | 0.01480925 | -1.1282453 |
| PDIA4 | 0.92517937 | 6.1646928 | 3.77517572 | 0.00044633 | 0.00898474 | -0.1107225 |
| HIST1H1B | 0.92216754 | 5.96153477 | 3.87894522 | 0.00032375 | 0.00799178 | 0.1807886 |
| SMARCC1 | 0.91066901 | 6.13625669 | 4.5628432 | 3.5958E-05 | 0.0037324 | 2.18812378 |
| CDK6 | 0.90171157 | 5.67107684 | 2.92487242 | 0.00527945 | 0.02979847 | -2.3259571 |
| NCAPD2 | 0.89440346 | 6.05081063 | 4.09212795 | 0.00016555 | 0.00608153 | 0.79146241 |
| CDKN2C | 0.89284445 | 5.91213499 | 3.20387528 | 0.00242991 | 0.02039581 | -1.6368936 |
| PLOD1 | 0.88603298 | 6.19395465 | 2.94128132 | 0.00504919 | 0.02916084 | -2.2865805 |
| MAN2B1 | 0.87989122 | 6.67548232 | 3.26940774 | 0.00201446 | 0.01844702 | -1.4692894 |
| CD276 | 0.87674411 | 6.39804988 | 3.44985299 | 0.00119056 | 0.01371396 | -0.9972964 |
| PXDN | 0.87573415 | 6.45174644 | 3.93731903 | 0.00026982 | 0.00736879 | 0.34647316 |
| CRELD2 | 0.86784976 | 6.31120103 | 3.10459385 | 0.00321623 | 0.02322543 | -1.8867465 |
| MT2A | 0.86625992 | 6.01298979 | 2.91396312 | 0.00543791 | 0.03030265 | -2.3520537 |
| PSPH | 0.86264458 | 5.81777516 | 3.70020539 | 0.00056154 | 0.0102901 | -0.3188194 |
| KPNA2 | 0.86036033 | 5.93536791 | 4.05508851 | 0.0001862 | 0.00626209 | 0.68428039 |
| POLA1 | 0.85756898 | 6.04270011 | 4.64270962 | 2.7602E-05 | 0.00363986 | 2.43068703 |
| PTN | 0.85743447 | 6.22907241 | 4.11096607 | 0.00015592 | 0.00590658 | 0.84614136 |
| MARCKSL1 | 0.85646085 | 6.23441658 | 4.67226839 | 2.502E-05 | 0.00348261 | 2.52081822 |
| FCGBP | 0.85139746 | 6.35602769 | 3.82385514 | 0.0003841 | 0.00863103 | 0.02553704 |
| P3H1 | 0.84996335 | 6.17210763 | 2.99376059 | 0.00437413 | 0.02719949 | -2.1596542 |
| RCC2 | 0.835023 | 6.15621205 | 3.40335344 | 0.00136515 | 0.01476331 | -1.1203543 |
| NTN1 | 0.83431367 | 6.87296691 | 3.0300928 | 0.00395728 | 0.02584525 | -2.0709086 |
| FABP7 | 0.83357974 | 6.13229815 | 3.96242471 | 0.0002494 | 0.00704713 | 0.41809518 |
| GGH | 0.83225979 | 6.64986726 | 4.86311589 | 1.3213E-05 | 0.00291583 | 3.10706668 |
| GALNS | 0.8262001 | 7.00659374 | 3.6734099 | 0.00060927 | 0.01058137 | -0.3926667 |
| SSR1 | 0.82399273 | 6.52791537 | 4.3374138 | 7.5251E-05 | 0.00471354 | 1.51172985 |
| NAMPT | 0.82032484 | 5.99754051 | 2.85963594 | 0.00629509 | 0.03282923 | -2.4810196 |
| CDK1 | 0.81911379 | 6.04120895 | 4.20817323 | 0.00011425 | 0.00550469 | 1.13002388 |
| DCTPP1 | 0.81605893 | 6.2780678 | 3.37640924 | 0.0014772 | 0.01520977 | -1.1912139 |
| CASP3 | 0.81560858 | 6.61020381 | 4.0439241 | 0.0001929 | 0.00633361 | 0.65205994 |
| ANP32A | 0.81314002 | 6.40201461 | 5.15885187 | 4.8501E-06 | 0.00291583 | 4.02834025 |
| NFIA | 0.81011312 | 6.03886718 | 3.82669575 | 0.00038074 | 0.00863088 | 0.03351532 |
| XPO5 | 0.80741053 | 6.1436665 | 4.82273214 | 1.5133E-05 | 0.00291583 | 2.98242132 |
| MKI67 | 0.80267289 | 5.59886132 | 3.25755863 | 0.00208422 | 0.01878423 | -1.499749 |
| YBX1 | 0.80066264 | 5.93341566 | 3.72970898 | 0.00051314 | 0.00963057 | -0.2371836 |
| SET.iso2 | 0.79740496 | 6.36213585 | 5.02965704 | 7.5275E-06 | 0.00291583 | 3.62414984 |
| VANGL2 | 0.79365109 | 6.31898483 | 3.67033047 | 0.00061499 | 0.01058137 | -0.4011353 |
| RPL32 | 0.79173588 | 6.51123363 | 4.27626857 | 9.1737E-05 | 0.00500079 | 1.33055313 |
| PUS7 | 0.79143926 | 6.27978173 | 4.61995547 | 2.9767E-05 | 0.003729 | 2.36143449 |
| MCM5 | 0.78719772 | 6.10270583 | 3.30214794 | 0.00183301 | 0.01738278 | -1.3847767 |
| TSR1 | 0.78624809 | 6.14656529 | 3.7836453 | 0.00043485 | 0.00895549 | -0.0870786 |
| SELENON | 0.78336104 | 6.23445325 | 3.6871123 | 0.00058439 | 0.0104232 | -0.3549388 |
| CALR | 0.78301795 | 6.76684422 | 3.91031414 | 0.0002936 | 0.00756514 | 0.26967575 |
| EHMT2 | 0.78228207 | 6.06026973 | 4.17276672 | 0.00012799 | 0.00567565 | 1.0262927 |
| FKBP9 | 0.78224442 | 6.12349336 | 3.52206036 | 0.00096096 | 0.01243573 | -0.8043139 |
| GLA | 0.78128097 | 6.48405302 | 3.74886951 | 0.00048389 | 0.00927368 | -0.1839866 |
| HNRNPAB | 0.77839583 | 6.11184185 | 3.65313095 | 0.00064795 | 0.01075126 | -0.4483654 |
| CBX3 | 0.77786081 | 6.11499125 | 4.22268373 | 0.00010904 | 0.00531361 | 1.17264254 |
| NHLRC3 | 0.7737066 | 6.71538927 | 3.5239146 | 0.00095566 | 0.01241367 | -0.7993285 |
| H6PD | 0.76839572 | 6.49044167 | 3.1420371 | 0.00289508 | 0.02201769 | -1.7931023 |
| SYNCRIP | 0.76580718 | 6.38034947 | 5.11288605 | 5.6728E-06 | 0.00291583 | 3.88425138 |
| RRM1 | 0.76458032 | 6.14322757 | 3.85455543 | 0.00034925 | 0.00829484 | 0.11191945 |
| HAT1 | 0.7636143 | 5.95814706 | 3.50098372 | 0.0010232 | 0.01278355 | -0.8608777 |
| BANF1 | 0.76209401 | 6.33246452 | 3.08390563 | 0.00340775 | 0.02401723 | -1.9381775 |
| KIAA0754 | 0.76175983 | 6.30687061 | 4.00705352 | 0.00021673 | 0.00666278 | 0.54594041 |
| TUBB2B | 0.75984466 | 6.23513664 | 3.78622737 | 0.0004314 | 0.00895549 | -0.0798651 |
| SPP1 | 0.75654954 | 6.31788707 | 3.0322189 | 0.00393408 | 0.02575019 | -2.0656935 |
| SSB | 0.74634063 | 6.4319564 | 4.89647506 | 1.1809E-05 | 0.00291583 | 3.21025658 |
| GOLIM4 | 0.74356457 | 6.2311391 | 3.81168011 | 0.00039883 | 0.00880411 | -0.0086248 |
| NME4 | 0.74328427 | 6.36276367 | 3.4514341 | 0.00118501 | 0.01369867 | -0.9930951 |
| HIRIP3 | 0.74203794 | 6.1073414 | 3.52463817 | 0.0009536 | 0.01241367 | -0.7973827 |
| RPLP2 | 0.73931291 | 6.61019794 | 3.6971653 | 0.00056677 | 0.0102901 | -0.327212 |
| NUDT1 | 0.73902207 | 6.18538326 | 4.01760824 | 0.00020963 | 0.00658513 | 0.576273 |
| NAGA | 0.73854425 | 6.44930338 | 3.91696093 | 0.00028756 | 0.00756514 | 0.28855457 |
| SPATA5 | 0.73850179 | 6.4042185 | 4.35420921 | 7.1254E-05 | 0.00456851 | 1.56167448 |
| PARP1 | 0.73803502 | 6.20633274 | 4.4387104 | 5.4084E-05 | 0.0043334 | 1.81408499 |
| AASS | 0.73732364 | 6.21975144 | 3.85082172 | 0.00035332 | 0.00829484 | 0.1013955 |
| ZNF326 | 0.73720846 | 6.37671633 | 4.56897203 | 3.5238E-05 | 0.0037324 | 2.20668643 |
| CALU | 0.73631513 | 6.28889436 | 2.72816399 | 0.00891456 | 0.04016492 | -2.7861051 |
| SEC24A | 0.73604738 | 6.67802097 | 4.17376872 | 0.00012758 | 0.00567565 | 1.02922314 |
| HMGB3 | 0.73358911 | 6.04464793 | 3.76823598 | 0.00045596 | 0.00898474 | -0.1300755 |
| RBBP4 | 0.73355723 | 6.15859718 | 4.3192884 | 7.9811E-05 | 0.00481845 | 1.45791592 |
| HMGN1 | 0.73306945 | 6.13345201 | 3.81489259 | 0.00039489 | 0.00875573 | 0.0003838 |
| RRP9 | 0.73215709 | 5.99238777 | 3.68948505 | 0.00058019 | 0.01042046 | -0.3483982 |
| WDR5 | 0.73136087 | 6.38793938 | 5.33251541 | 2.6762E-06 | 0.00263481 | 4.57527518 |
| DPY30 | 0.7295143 | 7.09876241 | 3.56259876 | 0.00085129 | 0.01193402 | -0.6949875 |
| ARAP3 | 0.72767719 | 6.06799244 | 3.13537346 | 0.00294993 | 0.02223203 | -1.8098203 |
| CDK2 | 0.72563073 | 6.02438019 | 3.43382798 | 0.00124818 | 0.01403453 | -1.0398147 |
| CRTAP | 0.72257305 | 6.41489647 | 2.72788399 | 0.00892108 | 0.04016492 | -2.786744 |
| KDM1A | 0.72073448 | 6.50071855 | 4.5583387 | 3.6497E-05 | 0.0037324 | 2.17448635 |
| HM13 | 0.71724921 | 6.24601409 | 3.84873195 | 0.00035561 | 0.00829484 | 0.09550742 |
| LSM7 | 0.71588892 | 6.33808495 | 4.88207371 | 1.2396E-05 | 0.00291583 | 3.16568424 |
| ATXN2L | 0.71442917 | 6.24353136 | 4.15960747 | 0.00013349 | 0.00576674 | 0.98783533 |
| SREK1 | 0.71422573 | 6.21866849 | 4.05603206 | 0.00018564 | 0.00626209 | 0.6870053 |
| PNO1 | 0.71235409 | 6.24004216 | 3.32162063 | 0.00173256 | 0.01676033 | -1.3342702 |
| DPP7 | 0.70688768 | 6.5406347 | 3.15765777 | 0.00277023 | 0.0216745 | -1.7538245 |
| DDR1 | 0.70444609 | 6.24511248 | 3.45251221 | 0.00118125 | 0.01369867 | -0.9902298 |
| TOP1 | 0.70254261 | 6.0775602 | 4.17536161 | 0.00012693 | 0.00567565 | 1.03388231 |
| ANP32B | 0.70222184 | 6.26793988 | 3.57611503 | 0.00081746 | 0.01180484 | -0.658382 |
| NIFK | 0.70202576 | 6.13114219 | 3.85245853 | 0.00035153 | 0.00829484 | 0.10600845 |
| HMGB1 | 0.70191159 | 6.30233573 | 4.35796007 | 7.039E-05 | 0.00456851 | 1.57283879 |
| PRPF4 | 0.70129707 | 6.36212612 | 5.05080935 | 7.006E-06 | 0.00291583 | 3.69015293 |
| BUD31 | 0.70057066 | 6.07012104 | 4.08620575 | 0.0001687 | 0.00608153 | 0.77429586 |
| U2AF2 | 0.70013698 | 6.18597197 | 4.27601477 | 9.1813E-05 | 0.00500079 | 1.32980327 |
| TROVE2 | 0.69981229 | 6.46779147 | 4.87946254 | 1.2506E-05 | 0.00291583 | 3.15760662 |
| NCL | 0.69943882 | 6.24499573 | 4.37240552 | 6.7157E-05 | 0.00456851 | 1.61587016 |
| PTBP1 | 0.69925904 | 6.05397503 | 2.98661468 | 0.00446081 | 0.02736001 | -2.1770254 |
| PTMS | 0.69915118 | 6.31705303 | 3.90251907 | 0.00030083 | 0.00761334 | 0.24755521 |
| SYNE2 | 0.69894967 | 6.18784011 | 3.71514046 | 0.00053652 | 0.00999436 | -0.2775367 |
| SCAF4 | 0.69810228 | 6.24055968 | 4.39675262 | 6.2032E-05 | 0.00456851 | 1.6885221 |
| MSH2 | 0.69782582 | 6.08738404 | 3.51223095 | 0.00098952 | 0.01259316 | -0.8307171 |
| RRP8 | 0.69750779 | 6.3192982 | 3.63271376 | 0.00068927 | 0.01110596 | -0.5042771 |
| PSMB9 | 0.69661461 | 6.40889389 | 3.01686742 | 0.00410452 | 0.02643669 | -2.1032949 |
| HLA.A.iso2 | 0.69611933 | 6.28612921 | 2.7741539 | 0.0079012 | 0.03743563 | -2.6805322 |
| DCPS | 0.69608628 | 6.34862284 | 3.97924524 | 0.00023656 | 0.00693228 | 0.46620123 |
| GALNT7 | 0.69449416 | 6.32083596 | 4.01272119 | 0.00021289 | 0.00658513 | 0.56222382 |
| SNRPD3 | 0.69417744 | 6.44195209 | 4.78548124 | 1.7146E-05 | 0.00291583 | 2.8677196 |
| UBA2 | 0.69385173 | 6.27100765 | 4.56562844 | 3.5629E-05 | 0.0037324 | 2.19655846 |
| IFI16 | 0.69126153 | 6.25040903 | 2.82652761 | 0.00687736 | 0.03463939 | -2.5587938 |
| HMGA1 | 0.69065647 | 6.0150028 | 2.90867246 | 0.00551634 | 0.03051036 | -2.3646859 |
| MOV10 | 0.6902923 | 6.23999575 | 3.26600087 | 0.00203429 | 0.01850057 | -1.4780541 |
| PTGFRN | 0.68730568 | 6.45966819 | 3.22278948 | 0.00230236 | 0.01981885 | -1.5887346 |
| TRIP10 | 0.68518688 | 6.40609953 | 3.67440462 | 0.00060743 | 0.01058137 | -0.3899304 |
| HDHD5 | 0.68516685 | 6.14602607 | 3.93510613 | 0.0002717 | 0.00736879 | 0.34017051 |
| P4HTM | 0.68514597 | 6.83748606 | 3.20925256 | 0.00239299 | 0.02022135 | -1.62322 |
| TRMT6 | 0.68241039 | 6.11674276 | 3.89299865 | 0.00030989 | 0.00773026 | 0.22056751 |
| PAPOLA | 0.68236728 | 6.22817362 | 4.09203767 | 0.0001656 | 0.00608153 | 0.79120061 |
| CROT | 0.68110992 | 6.35191367 | 3.30717493 | 0.00180656 | 0.01722988 | -1.3717553 |
| G3BP1 | 0.680802 | 6.14552939 | 3.89736731 | 0.0003057 | 0.00769778 | 0.23294745 |
| PHF6 | 0.68065578 | 6.05125337 | 3.40831447 | 0.00134542 | 0.01472034 | -1.1072716 |
| ZBTB20 | 0.67994626 | 6.26182939 | 2.74715454 | 0.0084824 | 0.03906739 | -2.7426629 |
| RFC2 | 0.67981076 | 6.01225101 | 3.24616766 | 0.00215344 | 0.01916674 | -1.528967 |
| ILF2 | 0.67921384 | 6.53366195 | 4.81052723 | 1.5766E-05 | 0.00291583 | 2.94481099 |
| SMARCD2 | 0.67841521 | 6.61871418 | 2.76520038 | 0.00808973 | 0.03780265 | -2.7011836 |
| MTA1 | 0.67799451 | 6.39689206 | 4.62114243 | 2.965E-05 | 0.003729 | 2.36504418 |
| APEX1 | 0.67656418 | 6.17887159 | 3.76393825 | 0.00046202 | 0.00900847 | -0.1420515 |
| SMARCA5 | 0.67619903 | 6.184407 | 4.02962936 | 0.00020182 | 0.00646196 | 0.61086448 |
| PDIA6 | 0.67504704 | 6.40649623 | 3.55121804 | 0.0008808 | 0.01203493 | -0.7257499 |
| GLB1 | 0.67267521 | 6.63285674 | 3.02927226 | 0.00396627 | 0.02584525 | -2.0729206 |
| NUP210 | 0.67240311 | 6.1778978 | 4.18694733 | 0.0001223 | 0.00567565 | 1.06779317 |
| QSOX2 | 0.67116265 | 6.49250522 | 3.68705279 | 0.0005845 | 0.0104232 | -0.3551029 |
| SUMF2 | 0.67057799 | 6.18573752 | 3.57016645 | 0.00083218 | 0.01186791 | -0.6745017 |
| CNPY4 | 0.67009231 | 6.4624376 | 2.91552386 | 0.00541497 | 0.03021649 | -2.3483243 |
| FEN1 | 0.66868621 | 5.98917156 | 3.7482076 | 0.00048487 | 0.00927368 | -0.1858267 |
| HDGF | 0.667223 | 6.24702591 | 4.22216818 | 0.00010922 | 0.00531361 | 1.17112724 |
| IMPDH2 | 0.66666539 | 6.34088238 | 4.14508409 | 0.00013984 | 0.00577125 | 0.94545226 |
| VWF | 0.66657049 | 6.35573035 | 3.11015411 | 0.00316651 | 0.02313029 | -1.872886 |
| BCAT1.iso5 | 0.6662548 | 6.40176029 | 2.69739885 | 0.00965793 | 0.04226716 | -2.856025 |
| NFIB | 0.66587834 | 6.10302826 | 2.998034 | 0.00432306 | 0.02719949 | -2.1492527 |
| MASP1 | 0.66493865 | 6.53243297 | 3.79117827 | 0.00042487 | 0.00895549 | -0.0660268 |
| THOC5 | 0.66457868 | 6.22483606 | 3.78730975 | 0.00042997 | 0.00895549 | -0.0768405 |
| MEPCE | 0.66361278 | 6.28456363 | 4.51149784 | 4.2591E-05 | 0.00395224 | 2.03295758 |
| HEXIM1 | 0.66248777 | 6.25441786 | 3.37673616 | 0.00147579 | 0.01520977 | -1.1903562 |
| GTF2I | 0.66106792 | 6.08380281 | 3.64034335 | 0.00067354 | 0.01099239 | -0.4834034 |
| RPS3A | 0.66040587 | 6.4920921 | 4.59868399 | 3.194E-05 | 0.0037324 | 2.29679848 |
| XRN2 | 0.6586296 | 6.29219729 | 4.01399184 | 0.00021204 | 0.00658513 | 0.56587588 |
| THOC2 | 0.65852265 | 6.34509747 | 4.04107664 | 0.00019465 | 0.00633361 | 0.64384864 |
| SCIN | 0.65829242 | 6.33896132 | 2.61515889 | 0.01193359 | 0.04783938 | -3.04011 |
| SIAE | 0.65745252 | 6.62793127 | 3.05373003 | 0.00370639 | 0.02499694 | -2.0127943 |
| SUPT16H | 0.65707614 | 6.17752244 | 3.6364218 | 0.00068159 | 0.01101751 | -0.4941353 |
| PTMA | 0.65573718 | 6.23440247 | 3.19487912 | 0.0024929 | 0.02066714 | -1.6597375 |
| THYN1 | 0.65509513 | 6.30778414 | 4.45005897 | 5.211E-05 | 0.00428075 | 1.84812376 |
| SRSF12 | 0.65406269 | 6.05213191 | 2.95771226 | 0.00482803 | 0.02838825 | -2.2470024 |
| CNPY2 | 0.65311332 | 6.51391657 | 3.05110768 | 0.00373347 | 0.02501127 | -2.0192561 |
| TEAD1 | 0.65225991 | 6.33419472 | 3.79140626 | 0.00042457 | 0.00895549 | -0.0653893 |
| WDR12 | 0.6516148 | 6.44055723 | 3.56957301 | 0.00083367 | 0.01186791 | -0.676109 |
| DHPS | 0.65131506 | 6.10528298 | 4.2417803 | 0.00010254 | 0.00519002 | 1.22882423 |
| NRDC | 0.64872882 | 6.56395013 | 4.85799665 | 1.3442E-05 | 0.00291583 | 3.09124926 |
| CDKAL1 | 0.64738622 | 6.38839248 | 4.58993306 | 3.2878E-05 | 0.0037324 | 2.27023729 |
| PLXDC1 | 0.64696115 | 6.38526295 | 3.67869062 | 0.00059956 | 0.01054176 | -0.3781357 |
| ADAM9 | 0.64537301 | 6.29302978 | 3.59494587 | 0.00077247 | 0.01155471 | -0.6072565 |
| RPL22 | 0.64446558 | 6.48879843 | 4.51816285 | 4.1666E-05 | 0.00393944 | 2.05306398 |
| GPX7 | 0.64439608 | 6.33423865 | 2.88589866 | 0.00586614 | 0.03161464 | -2.4188826 |
| DDOST | 0.64314714 | 6.46300401 | 3.63931483 | 0.00067564 | 0.01099239 | -0.4862187 |
| ARHGDIB | 0.64159154 | 6.48079128 | 2.80517185 | 0.00727902 | 0.03559294 | -2.608626 |
| IVNS1ABP | 0.64149753 | 6.42852108 | 4.0005329 | 0.00022123 | 0.00671871 | 0.52721962 |
| BCCIP | 0.64129524 | 6.5121006 | 3.826115 | 0.00038142 | 0.00863088 | 0.03188397 |
| SLC2A5 | 0.64044586 | 6.47605308 | 3.18938941 | 0.00253209 | 0.02082575 | -1.6736577 |
| ITGB8 | 0.6403842 | 6.36350769 | 3.07648588 | 0.00347901 | 0.02411251 | -1.9565689 |
| MSI2 | 0.63989095 | 6.2863325 | 3.29126069 | 0.00189156 | 0.0176182 | -1.4129368 |
| AKAP13 | 0.63969295 | 6.41290284 | 3.23029702 | 0.00225351 | 0.01946951 | -1.5695703 |
| CPSF2 | 0.63648241 | 6.2497982 | 4.59920235 | 3.1885E-05 | 0.0037324 | 2.29837237 |
| KDM3B | 0.63582137 | 6.21646962 | 3.55491995 | 0.0008711 | 0.01199194 | -0.7157495 |
| SERPINB8 | 0.63543665 | 6.4264422 | 3.47348738 | 0.00111019 | 0.01318284 | -0.9343812 |
| PRPF40A | 0.63475425 | 6.3241992 | 4.06396549 | 0.00018103 | 0.00625632 | 0.70992799 |
| EEF2 | 0.6338074 | 6.34581722 | 4.74902175 | 1.937E-05 | 0.0030333 | 2.75571748 |
| SMARCA4 | 0.6332809 | 6.03761023 | 3.06470723 | 0.00359502 | 0.0245766 | -1.9857056 |
| LMNB1 | 0.63297132 | 6.02484867 | 2.5886516 | 0.01276565 | 0.04981599 | -3.0985539 |
| ANAPC7 | 0.63267193 | 6.57046002 | 5.16561821 | 4.7394E-06 | 0.00291583 | 4.04957593 |
| SNRPE | 0.63203962 | 6.73098143 | 3.58153266 | 0.00080426 | 0.01180484 | -0.6436883 |
| NUP43 | 0.63117873 | 6.16423891 | 3.56762299 | 0.00083856 | 0.01190372 | -0.6813895 |
| IQGAP2 | 0.63027802 | 6.29233428 | 2.7649933 | 0.00809414 | 0.03780265 | -2.7016606 |
| EFTUD2 | 0.62953242 | 6.32589743 | 4.79011325 | 1.6882E-05 | 0.00291583 | 2.88196772 |
| NHP2 | 0.62829285 | 6.22651728 | 3.53327607 | 0.00092934 | 0.01241367 | -0.7741365 |
| LIG3 | 0.62813122 | 6.15023592 | 3.40514387 | 0.001358 | 0.01476331 | -1.1156341 |
| POLR1A | 0.6259281 | 6.33418136 | 3.81013183 | 0.00040074 | 0.00880751 | -0.0129652 |
| ZC3HAV1L | 0.625201 | 6.30514968 | 2.61264696 | 0.01201026 | 0.04801761 | -3.0456671 |
| DNMT1 | 0.62479237 | 6.05809567 | 3.26126001 | 0.00206219 | 0.01868647 | -1.4902414 |
| VIRMA | 0.6240062 | 6.33121847 | 4.36660795 | 6.8436E-05 | 0.00456851 | 1.5985932 |
| RPL38 | 0.62374299 | 6.52536498 | 3.51123753 | 0.00099245 | 0.01259316 | -0.8333832 |
| H1FX | 0.62341767 | 6.32441602 | 4.18398683 | 0.00012347 | 0.00567565 | 1.05912411 |
| DCK | 0.6209719 | 6.50075183 | 3.25061399 | 0.00212617 | 0.01895767 | -1.5175696 |
| DDX47 | 0.62005724 | 6.39841386 | 3.47662549 | 0.00109992 | 0.01314031 | -0.926009 |
| HDAC1 | 0.61793119 | 6.25904819 | 3.0495472 | 0.00374967 | 0.02506205 | -2.0230997 |
| LGALS3BP | 0.61761265 | 6.65519075 | 2.90454849 | 0.00557821 | 0.03075072 | -2.3745216 |
| HCFC1.iso4 | 0.61751472 | 6.340944 | 4.78012267 | 1.7457E-05 | 0.00291583 | 2.85124183 |
| RPL28 | 0.61726661 | 6.40823198 | 4.15061634 | 0.00013739 | 0.00577125 | 0.96158925 |
| XAB2 | 0.61708484 | 6.24532858 | 3.70702731 | 0.00054997 | 0.01013203 | -0.2999734 |
| TRIM27 | 0.6168962 | 6.53888579 | 4.579972 | 3.398E-05 | 0.0037324 | 2.24002422 |
| HSPA5 | 0.61676668 | 6.46008934 | 3.68273715 | 0.00059223 | 0.01044945 | -0.3669934 |
| STT3B | 0.61638802 | 6.57650229 | 2.68804552 | 0.00989499 | 0.0429669 | -2.8771694 |
| KDELC2 | 0.61638665 | 6.43559068 | 3.20086797 | 0.0024508 | 0.02052043 | -1.6445345 |
| DTYMK | 0.61633618 | 6.29848791 | 3.25823418 | 0.00208018 | 0.01878162 | -1.4980143 |
| RCC1 | 0.61566232 | 6.15179517 | 3.21766024 | 0.00233631 | 0.01991029 | -1.601812 |
| CHD4 | 0.61449484 | 6.11955623 | 3.48086536 | 0.00108618 | 0.01305238 | -0.9146906 |
| MTA2 | 0.61436515 | 6.13499213 | 3.66245136 | 0.00062989 | 0.01059182 | -0.4227861 |
| PTPN12 | 0.61404068 | 6.21751792 | 3.18624638 | 0.00255478 | 0.02094894 | -1.6816207 |
| HEXB | 0.61276407 | 6.48187476 | 2.71622312 | 0.0091966 | 0.04089098 | -2.8133106 |
| FMC1 | 0.61183879 | 6.18001612 | 3.65550603 | 0.0006433 | 0.0107362 | -0.4418505 |
| NOL11 | 0.61062089 | 6.31642338 | 3.55855211 | 0.00086167 | 0.01193402 | -0.7059319 |
| RPLP0 | 0.6098533 | 6.50470616 | 4.24859312 | 0.00010031 | 0.00517457 | 1.2488929 |
| PSME3 | 0.60911396 | 6.20057695 | 3.74539966 | 0.00048907 | 0.00929258 | -0.1936307 |
| MAN2B2 | 0.60855708 | 6.65075351 | 3.07972342 | 0.00344775 | 0.02408712 | -1.9485475 |
| DBR1 | 0.60777734 | 6.27267289 | 3.47622833 | 0.00110121 | 0.01314031 | -0.9270688 |
| PHAX | 0.60766317 | 6.22104486 | 3.48601011 | 0.00106973 | 0.01297921 | -0.9009461 |
| HNRNPD | 0.6075599 | 6.61522803 | 3.35024917 | 0.00159431 | 0.01600993 | -1.2596931 |
| HAUS6 | 0.60739305 | 6.28950991 | 3.17362529 | 0.00264787 | 0.02133198 | -1.7135472 |
| P4HB | 0.60738271 | 6.40751809 | 2.86581835 | 0.00619157 | 0.03255439 | -2.4664273 |
| TOP2B | 0.6059579 | 6.30995161 | 3.9769643 | 0.00023827 | 0.00694158 | 0.45967225 |
| IDH1 | 0.60570139 | 6.30790772 | 3.25642338 | 0.00209102 | 0.01881171 | -1.5026638 |
| LARP7 | 0.60476795 | 6.29925031 | 4.0694175 | 0.00017793 | 0.00625453 | 0.72569262 |
| IFI35 | 0.60334772 | 6.48099296 | 2.69479341 | 0.00972344 | 0.04241961 | -2.8619203 |
| MSI1 | 0.60227644 | 6.10527321 | 3.41424247 | 0.00132221 | 0.01459377 | -1.0916242 |
| HSP90B1 | 0.60204511 | 6.57945065 | 3.33677487 | 0.00165802 | 0.01634147 | -1.2948412 |
| DLG5 | 0.60200572 | 6.32572657 | 3.96124416 | 0.00025033 | 0.00704713 | 0.41472245 |
| GAR1 | 0.60127067 | 6.28753537 | 3.28765471 | 0.00191134 | 0.01776939 | -1.4222513 |
| PLA2G4A | 0.60094346 | 6.51458409 | 3.15607039 | 0.00278268 | 0.02169947 | -1.7578216 |
| PPP4R1 | 0.60062399 | 6.40306883 | 3.75649197 | 0.00047271 | 0.00917837 | -0.1627847 |
| FIP1L1 | 0.60039424 | 6.47654934 | 2.98449426 | 0.00448684 | 0.02738557 | -2.1821747 |
| RPS12 | 0.59979505 | 6.48109436 | 4.35648803 | 7.0727E-05 | 0.00456851 | 1.56845688 |
| IFT81 | 0.59968226 | 6.46288669 | 4.13437407 | 0.0001447 | 0.00577125 | 0.91423882 |
| RPL6 | 0.5983336 | 6.55414154 | 4.35090235 | 7.2024E-05 | 0.00456851 | 1.55183486 |
| POFUT1 | 0.59828773 | 6.35428953 | 3.41450599 | 0.00132118 | 0.01459377 | -1.0909283 |
| IPO4 | 0.59798856 | 6.12889365 | 2.60241911 | 0.0123271 | 0.04871537 | -3.0682537 |
| PCDHGC3 | 0.59797005 | 6.73117295 | 3.09262196 | 0.00332579 | 0.02367145 | -1.9165357 |
| PKN2 | 0.5974143 | 6.40060523 | 3.98288066 | 0.00023387 | 0.00693228 | 0.47661095 |
| RUVBL1 | 0.59735115 | 6.5508702 | 3.80804831 | 0.00040333 | 0.00882565 | -0.0188046 |
| METTL2B | 0.5962353 | 6.30781195 | 3.43018407 | 0.00126164 | 0.01414339 | -1.049467 |
| PRPF38A | 0.59579911 | 6.24257102 | 3.09240308 | 0.00332783 | 0.02367145 | -1.9170796 |
| ADNP | 0.59576547 | 6.20546181 | 4.22494201 | 0.00010825 | 0.00531361 | 1.17928082 |
| RPS19 | 0.59383103 | 6.43654957 | 3.52138718 | 0.00096289 | 0.01243573 | -0.8061235 |
| TRMT5 | 0.59334877 | 6.22931195 | 3.18899835 | 0.0025349 | 0.02082575 | -1.6746488 |
| MSH6 | 0.59315437 | 6.0802957 | 2.97790531 | 0.00456863 | 0.02761566 | -2.1981599 |
| CIZ1 | 0.59155418 | 6.1929074 | 3.14242316 | 0.00289193 | 0.02201769 | -1.7921331 |
| RPS23 | 0.59092022 | 6.76600588 | 3.52770495 | 0.00094492 | 0.01241367 | -0.7891331 |
| ADA | 0.58995673 | 6.46286345 | 3.0798119 | 0.0034469 | 0.02408712 | -1.9483282 |
| VRK1 | 0.58973658 | 6.18669083 | 3.41874631 | 0.00130482 | 0.01456226 | -1.0797255 |
| CLIC4 | 0.58868822 | 6.43011268 | 3.07334256 | 0.00350962 | 0.02422414 | -1.9643516 |
| RPL23 | 0.58797809 | 6.49465676 | 4.2979033 | 8.5537E-05 | 0.00500079 | 1.39453982 |
| RB1 | 0.58746913 | 6.14647526 | 2.70447751 | 0.00948201 | 0.04167925 | -2.8399877 |
| GRWD1 | 0.58745697 | 6.28459999 | 3.66792109 | 0.00061951 | 0.01058137 | -0.4077586 |
| CSTF1 | 0.58744614 | 6.40460629 | 4.28592005 | 8.8919E-05 | 0.00500079 | 1.3590821 |
| HNRNPU | 0.5872241 | 6.26595244 | 4.11079054 | 0.000156 | 0.00590658 | 0.84563136 |
| HPF1 | 0.58667383 | 6.29034836 | 3.64152181 | 0.00067115 | 0.01099057 | -0.4801772 |
| SRRT | 0.58586141 | 6.26989325 | 4.11632714 | 0.00015328 | 0.00590658 | 0.86172256 |
| SNRPB2 | 0.58559564 | 6.29942635 | 3.96954382 | 0.00024389 | 0.00698355 | 0.43844387 |
| FOXK1 | 0.58490346 | 6.3266869 | 3.6831323 | 0.00059151 | 0.01044945 | -0.3659049 |
| CREBBP | 0.58443993 | 6.10251887 | 3.53397422 | 0.0009274 | 0.01241367 | -0.7722562 |
| TMX1 | 0.58434507 | 6.21404469 | 2.99689071 | 0.00433667 | 0.02719949 | -2.1520364 |
| ZHX1 | 0.58349237 | 6.39507992 | 3.91483936 | 0.00028948 | 0.00756514 | 0.28252702 |
| CBX5 | 0.58300399 | 6.1274766 | 2.82812683 | 0.00684813 | 0.03455786 | -2.5550516 |
| POLR1C | 0.58212155 | 6.40014043 | 4.69846003 | 2.2931E-05 | 0.003283 | 2.60083841 |
| ACTL6A | 0.58164069 | 6.27969983 | 4.06816044 | 0.00017864 | 0.00625453 | 0.72205696 |
| ATAT1 | 0.58123063 | 6.34456778 | 2.82865567 | 0.00683848 | 0.03454399 | -2.5538138 |
| STAG1 | 0.58044675 | 6.41356336 | 3.93134712 | 0.00027492 | 0.00736879 | 0.32946824 |
| RPL26 | 0.57928895 | 6.43750929 | 4.04912754 | 0.00018975 | 0.00633361 | 0.66707202 |
| TMEM214 | 0.57731699 | 6.30789067 | 3.40684898 | 0.00135122 | 0.01475158 | -1.1111374 |
| AQR | 0.57666054 | 6.58450337 | 4.41614582 | 5.8226E-05 | 0.0044048 | 1.74650204 |
| SLC16A1 | 0.57645427 | 6.24064221 | 2.63471395 | 0.01135189 | 0.04626716 | -2.9967145 |
| HNRNPC | 0.57604463 | 6.37448554 | 4.08727347 | 0.00016812 | 0.00608153 | 0.77739001 |
| RPL3 | 0.57590359 | 6.46872251 | 4.26196066 | 9.6076E-05 | 0.00506776 | 1.28830851 |
| RHOC | 0.57520144 | 6.35591084 | 2.79303532 | 0.00751686 | 0.03632806 | -2.6368279 |
| POP1 | 0.57487496 | 6.33971512 | 3.33091909 | 0.00168645 | 0.0164383 | -1.3100897 |
| DCTD | 0.57483578 | 6.39041093 | 3.21963679 | 0.00232317 | 0.01989985 | -1.5967741 |
| ABCF1 | 0.57477126 | 6.33314224 | 3.97183019 | 0.00024214 | 0.00697341 | 0.44498268 |
| PHF3 | 0.57391089 | 6.45762858 | 4.26269066 | 9.585E-05 | 0.00506776 | 1.29046245 |
| BUB3 | 0.5732307 | 6.30878036 | 4.10029895 | 0.0001613 | 0.00603201 | 0.81516563 |
| PLXNA3 | 0.57288745 | 6.46670814 | 4.08848956 | 0.00016748 | 0.00608153 | 0.78091456 |
| SSR4 | 0.57260635 | 6.44256833 | 3.24312917 | 0.00217226 | 0.01924916 | -1.5367501 |
| CTDSPL2 | 0.57242383 | 6.27796481 | 3.17635431 | 0.00262748 | 0.02120175 | -1.7066506 |
| PRPF31 | 0.57238402 | 6.39229881 | 3.58761268 | 0.0007897 | 0.01166455 | -0.6271835 |
| EXOSC10 | 0.57030578 | 6.45631162 | 4.1497479 | 0.00013777 | 0.00577125 | 0.9590555 |
| EXOSC4 | 0.57002345 | 6.27108647 | 3.37645861 | 0.00147699 | 0.01520977 | -1.1910844 |
| SETMAR | 0.56977945 | 6.21927332 | 3.11258218 | 0.00314502 | 0.02307362 | -1.8668283 |
| CEBPZ | 0.56896588 | 6.14979525 | 2.91247348 | 0.00545989 | 0.03036569 | -2.355612 |
| KHDRBS1 | 0.56895605 | 6.3125379 | 3.49299494 | 0.00104778 | 0.01278355 | -0.8822673 |
| RBBP7 | 0.56880785 | 6.11777038 | 3.37745335 | 0.0014727 | 0.01520977 | -1.1884742 |
| NOVA1 | 0.56762 | 6.24533411 | 3.1512072 | 0.00282116 | 0.02175832 | -1.7700593 |
| BZW2 | 0.56750154 | 6.32938847 | 4.14456564 | 0.00014007 | 0.00577125 | 0.94394046 |
| SEMA5A | 0.56633777 | 6.44673502 | 3.79251537 | 0.00042312 | 0.00895549 | -0.0622878 |
| NOS1AP | 0.56602244 | 6.19767008 | 2.65291021 | 0.01083406 | 0.04527895 | -2.9561223 |
| NOP9 | 0.565846 | 6.21985981 | 3.07980638 | 0.00344695 | 0.02408712 | -1.9483419 |
| NCBP1 | 0.56528264 | 6.23818135 | 3.84335307 | 0.0003616 | 0.00830024 | 0.08035929 |
| ESF1 | 0.56517821 | 5.98912852 | 2.86544688 | 0.00619774 | 0.03255439 | -2.4673047 |
| PHF2 | 0.56509715 | 6.15749814 | 3.12732756 | 0.00301746 | 0.02248345 | -1.8299759 |
| BCL2L2 | 0.56483382 | 6.3657583 | 4.28454019 | 8.9317E-05 | 0.00500079 | 1.35500174 |
| PCID2 | 0.56431288 | 6.19752237 | 3.36164403 | 0.00154225 | 0.01565691 | -1.2299036 |
| SAE1 | 0.56430554 | 6.31566674 | 3.95210107 | 0.00025761 | 0.00721169 | 0.38861756 |
| RPL18 | 0.56420454 | 6.42237423 | 3.6170496 | 0.00072268 | 0.01138788 | -0.5470584 |
| MED18 | 0.56419969 | 6.23054051 | 3.19873141 | 0.00246574 | 0.02053048 | -1.6499603 |
| RIPK1 | 0.56377162 | 6.35579474 | 3.44118362 | 0.00122141 | 0.01387862 | -1.0203126 |
| CLN5 | 0.56366391 | 6.50979175 | 2.74864302 | 0.00844936 | 0.038951 | -2.7392488 |
| THOC1 | 0.56304802 | 6.24810525 | 3.36418427 | 0.00153087 | 0.01562361 | -1.2232545 |
| RNASET2 | 0.56269236 | 6.63296687 | 3.12589331 | 0.00302965 | 0.02248345 | -1.8335653 |
| SIRT6 | 0.56249759 | 6.65763767 | 3.27535057 | 0.00198031 | 0.0182414 | -1.4539871 |
| TARS | 0.5624925 | 6.52385055 | 4.29233464 | 8.7093E-05 | 0.00500079 | 1.37805749 |
| PAPSS1 | 0.56242352 | 6.24819057 | 3.39654029 | 0.00139269 | 0.01489237 | -1.1383032 |
| HMGCS1 | 0.5618287 | 5.83677663 | 3.38808721 | 0.0014276 | 0.01508704 | -1.1605431 |
| RPS5 | 0.56071293 | 6.45627321 | 4.35435327 | 7.122E-05 | 0.00456851 | 1.5621032 |
| GPC1 | 0.56038739 | 6.38530149 | 4.45405875 | 5.1432E-05 | 0.00428075 | 1.86012838 |
| TAF7 | 0.56018821 | 6.26444719 | 3.23626407 | 0.00221538 | 0.01937392 | -1.5543186 |
| SEC61A1 | 0.56018221 | 6.22387684 | 3.0888351 | 0.00336117 | 0.02382162 | -1.9259429 |
| WDR92 | 0.56001744 | 6.46306293 | 3.52589645 | 0.00095003 | 0.01241367 | -0.7939984 |
| MRTO4 | 0.55948303 | 6.1435387 | 3.23502771 | 0.00222323 | 0.01940869 | -1.5574802 |
| SIPA1 | 0.55901907 | 6.32245768 | 2.98926764 | 0.00442845 | 0.02735833 | -2.1705795 |
| PPP4R2 | 0.55892937 | 6.22695621 | 3.69244381 | 0.00057498 | 0.01040153 | -0.3402391 |
| ARID2 | 0.55862131 | 6.20222558 | 2.99554836 | 0.0043527 | 0.02719949 | -2.155304 |
| RAD21 | 0.55830299 | 6.31148728 | 2.96915976 | 0.00467933 | 0.02794772 | -2.2193407 |
| FKBP5 | 0.55782767 | 6.2743731 | 3.07807437 | 0.00346364 | 0.02408712 | -1.9526339 |
| UGGT2 | 0.55776545 | 6.1992265 | 2.85885484 | 0.00630828 | 0.03285947 | -2.4828617 |
| GTF3C2 | 0.55623932 | 6.47328177 | 3.57104705 | 0.00082999 | 0.01186791 | -0.6721163 |
| MPG | 0.555988 | 6.20781923 | 3.24165643 | 0.00218145 | 0.01924916 | -1.540521 |
| GTPBP4 | 0.55471662 | 6.18743124 | 3.36136549 | 0.00154351 | 0.01565691 | -1.2306325 |
| SH3PXD2B | 0.55450825 | 6.24660148 | 2.94113417 | 0.00505121 | 0.02916084 | -2.2869343 |
| SMAD1 | 0.55365731 | 6.21386435 | 2.97813421 | 0.00456576 | 0.02761566 | -2.197605 |
| FLT1 | 0.55319638 | 6.43102069 | 3.39024977 | 0.00141859 | 0.0150287 | -1.1548565 |
| UBE2I | 0.55297494 | 6.37664364 | 4.17501355 | 0.00012707 | 0.00567565 | 1.03286419 |
| BRAT1 | 0.55274113 | 6.03884644 | 3.02755881 | 0.00398511 | 0.0259006 | -2.077121 |
| RBM28 | 0.55260639 | 6.19767407 | 3.22064567 | 0.00231649 | 0.01987662 | -1.594202 |
| NUP133 | 0.55181836 | 6.46622619 | 4.07633935 | 0.00017407 | 0.00620994 | 0.74572103 |
| CTDP1 | 0.55135075 | 6.71737101 | 3.71205521 | 0.0005416 | 0.01001453 | -0.2860719 |
| PSMB8 | 0.55124906 | 6.58579072 | 2.84100957 | 0.00661678 | 0.03386792 | -2.5248517 |
| RBM39 | 0.55106591 | 6.20826058 | 3.43551005 | 0.00124201 | 0.01401733 | -1.0353571 |
| RPL9 | 0.55090348 | 6.66253558 | 4.01653642 | 0.00021034 | 0.00658513 | 0.57319107 |
| SMCHD1 | 0.54956543 | 6.20616213 | 2.81737835 | 0.00704686 | 0.03515867 | -2.5801753 |
| PALD1 | 0.54879093 | 6.44721552 | 3.5872248 | 0.00079062 | 0.01166455 | -0.6282369 |
| SGTA | 0.54792042 | 6.40547525 | 4.17676506 | 0.00012636 | 0.00567565 | 1.037988 |
| DEK | 0.54737669 | 6.51061692 | 2.66738559 | 0.01043768 | 0.04430855 | -2.9236857 |
| RPA3 | 0.54651575 | 6.48386977 | 4.71574608 | 2.1647E-05 | 0.00319032 | 2.65372931 |
| IRF2BP2 | 0.54601426 | 6.28007688 | 3.10883498 | 0.00317824 | 0.02314848 | -1.8761757 |
| PBDC1 | 0.54409515 | 6.41689243 | 4.02862948 | 0.00020246 | 0.00646196 | 0.60798544 |
| CLPX | 0.5435957 | 6.49381726 | 3.84847751 | 0.0003559 | 0.00829484 | 0.09479064 |
| NID1 | 0.5422787 | 6.35041298 | 2.89333486 | 0.00574971 | 0.03121541 | -2.4012181 |
| MBD3 | 0.54154954 | 6.07495223 | 3.05396764 | 0.00370395 | 0.02499694 | -2.0122086 |
| NELFB.iso2 | 0.54106716 | 6.23450058 | 3.59830931 | 0.00076468 | 0.01154166 | -0.5981093 |
| RPL31 | 0.54074806 | 6.43569983 | 3.69001965 | 0.00057924 | 0.01042046 | -0.3469243 |
| PNN | 0.54022973 | 6.32350548 | 3.96561052 | 0.00024692 | 0.00703018 | 0.42719911 |
| RASSF2 | 0.53955139 | 6.28568415 | 3.4011325 | 0.00137407 | 0.01480748 | -1.1262076 |
| HIP1 | 0.53954651 | 6.22032031 | 3.7336993 | 0.00050691 | 0.00954944 | -0.2261166 |
| RPL5 | 0.53942411 | 6.43141602 | 3.93916006 | 0.00026827 | 0.00736879 | 0.35171794 |
| JPT2 | 0.53934367 | 6.00768701 | 2.72362477 | 0.00902082 | 0.0403962 | -2.7964571 |
| BZW1 | 0.53906209 | 6.4043187 | 3.44318032 | 0.00121424 | 0.01387862 | -1.0150145 |
| H3C1 | 0.53859305 | 6.11199269 | 2.78503386 | 0.00767757 | 0.03688619 | -2.6553741 |
| RPS14 | 0.53854694 | 6.38008602 | 3.60861863 | 0.0007413 | 0.01152648 | -0.5700433 |
| GALC | 0.53778632 | 6.66833894 | 2.85808071 | 0.00632138 | 0.03289352 | -2.484687 |
| GTF3C3 | 0.53742417 | 6.18931637 | 3.29798253 | 0.00185521 | 0.0174745 | -1.3955573 |
| PPAT | 0.53693005 | 6.37964575 | 2.61017989 | 0.01208599 | 0.04811585 | -3.0511212 |
| ZMYM3 | 0.53619891 | 6.17810683 | 3.08834899 | 0.00336574 | 0.02382162 | -1.92715 |
| MBD2 | 0.53606761 | 6.21882008 | 2.65485787 | 0.01077993 | 0.04515661 | -2.9517655 |
| CTPS2 | 0.53527854 | 6.41234804 | 3.98568845 | 0.00023182 | 0.0069145 | 0.48465387 |
| DNAJC9 | 0.53482054 | 6.09122035 | 2.93806729 | 0.00509354 | 0.0292313 | -2.294305 |
| BOP1 | 0.53428123 | 6.23498968 | 3.10171732 | 0.00324224 | 0.02324302 | -1.8939108 |
| STAG2 | 0.53409245 | 6.20342599 | 3.15019325 | 0.00282924 | 0.02177779 | -1.7726093 |
| NSUN2 | 0.53383401 | 6.27858445 | 3.79363837 | 0.00042166 | 0.00895549 | -0.0591471 |
| RPS4X | 0.53357434 | 6.48746305 | 3.66722719 | 0.00062082 | 0.01058137 | -0.4096657 |
| CPNE2 | 0.53329882 | 6.23315499 | 3.10403739 | 0.00322125 | 0.02322543 | -1.8881328 |
| RPL36A | 0.53225431 | 6.28505326 | 3.93223428 | 0.00027415 | 0.00736879 | 0.33199362 |
| KNOP1 | 0.53216443 | 6.23330967 | 3.00577776 | 0.00423191 | 0.02706131 | -2.1303792 |
| RPL18A | 0.53203918 | 6.49031465 | 3.48349275 | 0.00107775 | 0.01303374 | -0.9076728 |
| PRPF40A.iso3 | 0.53099922 | 6.2822454 | 3.45189945 | 0.00118339 | 0.01369867 | -0.9918584 |
| MALT1 | 0.53042241 | 6.20725966 | 3.1511273 | 0.0028218 | 0.02175832 | -1.7702602 |
| MED8 | 0.53004091 | 6.25093006 | 3.60259003 | 0.00075489 | 0.01153275 | -0.5864608 |
| RPL4 | 0.52954871 | 6.48719728 | 3.77963991 | 0.00044024 | 0.00895549 | -0.0982635 |
| GAA | 0.52925443 | 6.5268978 | 2.76541879 | 0.00808508 | 0.03780265 | -2.7006804 |
| GRIK3 | 0.52875954 | 6.40708791 | 2.61222407 | 0.01202321 | 0.04801761 | -3.0466023 |
| MYG1 | 0.52867436 | 6.46858928 | 3.99157838 | 0.00022756 | 0.00682814 | 0.50153414 |
| RP2 | 0.52856017 | 6.59303681 | 3.76933935 | 0.00045441 | 0.00898474 | -0.1269997 |
| PAF1 | 0.52841909 | 6.17511267 | 3.15529118 | 0.00278882 | 0.02169947 | -1.7597832 |
| PCF11 | 0.52795342 | 6.17260134 | 3.23284364 | 0.00223716 | 0.01941461 | -1.5630633 |
| SUPT6H | 0.52748328 | 6.35870163 | 4.13994712 | 0.00014215 | 0.00577125 | 0.93047662 |
| PRPF4B | 0.52740425 | 6.24969624 | 3.07785737 | 0.00346574 | 0.02408712 | -1.9531715 |
| RPL35A | 0.52727624 | 6.36871756 | 3.29581398 | 0.00186686 | 0.01751842 | -1.4011664 |
| IPO8 | 0.52699122 | 6.30512711 | 3.33243272 | 0.00167906 | 0.01640112 | -1.3061498 |
| HNRNPH1 | 0.52633897 | 6.26191365 | 3.1352045 | 0.00295133 | 0.02223203 | -1.8102438 |
| EXOSC8 | 0.52607251 | 6.21859147 | 3.05128673 | 0.00373162 | 0.02501127 | -2.0188151 |
| WDR3 | 0.52545834 | 6.19650019 | 2.77238436 | 0.00793813 | 0.0375264 | -2.6846174 |
| RPL7 | 0.52496185 | 6.53339853 | 3.61006251 | 0.00073807 | 0.01152648 | -0.566109 |
| SF3A3 | 0.52376077 | 6.38632086 | 3.15996427 | 0.00275223 | 0.0216745 | -1.7480144 |
| TMED5 | 0.52370802 | 6.44973794 | 3.16986284 | 0.00267624 | 0.02135898 | -1.7230494 |
| OTUD6B | 0.52349411 | 6.43993541 | 4.13595724 | 0.00014398 | 0.00577125 | 0.91885063 |
| CMTR1 | 0.52263908 | 6.35316888 | 3.90419529 | 0.00029926 | 0.00761208 | 0.25231011 |
| PPP4R3A | 0.52247795 | 6.29701762 | 3.11100206 | 0.00315899 | 0.02310905 | -1.8707708 |
| RPS16 | 0.5218014 | 6.40482822 | 3.44252766 | 0.00121658 | 0.01387862 | -1.0167465 |
| POGK | 0.52152034 | 6.23153855 | 3.53332771 | 0.00092919 | 0.01241367 | -0.7739974 |
| SF3B5 | 0.52124676 | 6.30679371 | 3.76510308 | 0.00046037 | 0.00900847 | -0.1388063 |
| SF3A1 | 0.52071549 | 6.32226006 | 3.51222016 | 0.00098956 | 0.01259316 | -0.830746 |
| POLR3C | 0.52001917 | 6.2121427 | 3.47618166 | 0.00110136 | 0.01314031 | -0.9271934 |
| HDAC2 | 0.51976705 | 6.38138012 | 3.64971913 | 0.00065469 | 0.01079156 | -0.4577202 |
| PPIL2 | 0.51933951 | 6.21025933 | 3.09213213 | 0.00333035 | 0.02367145 | -1.917753 |
| ALYREF | 0.5182379 | 6.22291009 | 3.52933485 | 0.00094034 | 0.01241367 | -0.784747 |
| TXNDC12 | 0.5182138 | 6.53188734 | 3.58687639 | 0.00079145 | 0.01166455 | -0.629183 |
| HYOU1 | 0.51811316 | 6.52763919 | 3.84448503 | 0.00036033 | 0.00830024 | 0.08354626 |
| SEC23B | 0.51785464 | 6.35714905 | 3.16778647 | 0.00269201 | 0.02139879 | -1.7282902 |
| MLH1 | 0.51755273 | 6.37289912 | 2.9856879 | 0.00447217 | 0.02738557 | -2.1792763 |
| SNRPF | 0.51749206 | 6.23919045 | 3.04548073 | 0.00379221 | 0.02523004 | -2.0331095 |
| ELAC2 | 0.51745878 | 6.28701505 | 3.95021518 | 0.00025914 | 0.00721416 | 0.3832366 |
| DPY19L1 | 0.51721152 | 6.27900486 | 3.18363632 | 0.00257378 | 0.02097105 | -1.6882297 |
| NUMA1 | 0.5169265 | 6.23517393 | 3.46035282 | 0.00115419 | 0.01348172 | -0.969376 |
| RPL15 | 0.51670212 | 6.39536644 | 3.71319087 | 0.00053972 | 0.01001453 | -0.2829305 |
| PDAP1 | 0.51634793 | 6.38261938 | 4.07512848 | 0.00017474 | 0.00620994 | 0.74221629 |
| PA2G4 | 0.51625222 | 6.42462367 | 4.36468344 | 6.8866E-05 | 0.00456851 | 1.59286006 |
| IPO7 | 0.51619937 | 6.61652729 | 3.7404355 | 0.00049656 | 0.00938975 | -0.20742 |
| HNRNPUL1 | 0.51579831 | 6.22170251 | 2.97187406 | 0.00464471 | 0.02790723 | -2.2127714 |
| RPS27L | 0.51524572 | 6.41869655 | 2.77461508 | 0.0078916 | 0.03743563 | -2.6794672 |
| NIPBL | 0.51425072 | 6.24842985 | 3.51142299 | 0.00099191 | 0.01259316 | -0.8328855 |
| TRIR | 0.51374378 | 6.18450517 | 3.18040122 | 0.0025975 | 0.02106165 | -1.6964166 |
| GPS2 | 0.51191842 | 6.24751063 | 3.25222301 | 0.00211638 | 0.01893409 | -1.5134428 |
| BYSL | 0.51140181 | 6.14967397 | 3.50525412 | 0.00101029 | 0.01268807 | -0.8494326 |
| RPS9 | 0.51125929 | 6.48178059 | 3.26865088 | 0.00201885 | 0.01844702 | -1.471237 |
| EML4 | 0.51074853 | 6.51300299 | 2.81648422 | 0.00706363 | 0.03515867 | -2.5822622 |
| PPP1R10 | 0.51036052 | 6.4149417 | 3.37415809 | 0.00148695 | 0.01526862 | -1.1971191 |
| ZNF207 | 0.50957649 | 6.26828074 | 3.81521265 | 0.0003945 | 0.00875573 | 0.00128152 |
| CRNKL1 | 0.50947333 | 6.21663406 | 2.9630116 | 0.00475865 | 0.02818631 | -2.234206 |
| COPG2 | 0.50945685 | 6.43930575 | 3.46925086 | 0.0011242 | 0.01325503 | -0.945677 |
| ZNF800 | 0.5092271 | 6.28283152 | 3.11459874 | 0.00312728 | 0.02297774 | -1.861795 |
| CCAR2 | 0.50896982 | 6.36607753 | 4.24585566 | 0.0001012 | 0.00517457 | 1.24082751 |
| RFC3 | 0.50812065 | 6.32948048 | 3.07041786 | 0.00353833 | 0.02433965 | -1.9715883 |
| DIS3 | 0.50810447 | 6.23158107 | 3.39332009 | 0.0014059 | 0.01495742 | -1.1467793 |
| ZPR1 | 0.50789476 | 6.3460549 | 4.02942104 | 0.00020195 | 0.00646196 | 0.61026462 |
| CDK2AP1 | 0.50607833 | 6.26245866 | 2.80649853 | 0.00725345 | 0.03559294 | -2.605538 |
| SNRPA | 0.50581272 | 6.33460833 | 3.53669511 | 0.0009199 | 0.0123914 | -0.7649264 |
| PPIB | 0.50470657 | 6.35550981 | 2.81638924 | 0.00706541 | 0.03515867 | -2.5824839 |
| RPS20 | 0.50469051 | 6.53847637 | 2.88883402 | 0.00581992 | 0.0314846 | -2.4119134 |
| ADRM1 | 0.50459668 | 6.77422801 | 3.49656669 | 0.00103672 | 0.01278355 | -0.8727075 |
| USP39 | 0.50447951 | 6.20727873 | 3.33276344 | 0.00167745 | 0.01640112 | -1.3052887 |
| NOP2 | 0.50370175 | 6.04502117 | 2.63896605 | 0.01122889 | 0.04604512 | -2.9872471 |
| GART | 0.50303327 | 6.50436583 | 3.59512937 | 0.00077204 | 0.01155471 | -0.6067576 |
| METTL3 | 0.50297381 | 6.39567371 | 3.3562846 | 0.00156653 | 0.01579456 | -1.2439222 |
| PRMT1 | 0.50272964 | 6.36330824 | 3.18127224 | 0.0025911 | 0.02104373 | -1.6942129 |
| TRIM33 | 0.50229587 | 6.19743554 | 2.68731733 | 0.00991367 | 0.04301073 | -2.8788133 |
| MOGS | 0.50214001 | 6.36469925 | 2.86098675 | 0.00627234 | 0.03280864 | -2.4778332 |
| ARID1A | 0.50207848 | 6.19483744 | 3.00291503 | 0.0042654 | 0.0271587 | -2.1373602 |
| SHMT2 | 0.50181286 | 5.95745291 | 2.65197526 | 0.01086013 | 0.04535008 | -2.958213 |
| TCEA1 | 0.50134382 | 6.07782162 | 2.7302016 | 0.00886724 | 0.04007072 | -2.7814542 |
| EIF6 | 0.50109567 | 6.3162768 | 3.7787638 | 0.00044143 | 0.00895549 | -0.1007092 |
| SELENOH | 0.50098227 | 6.38092357 | 3.18573117 | 0.00255852 | 0.02094894 | -1.6829256 |
| ELAVL1 | 0.50049245 | 6.54493712 | 2.89046561 | 0.00579438 | 0.03140538 | -2.4080376 |
| MINK1 | -0.5069104 | 6.44091737 | -2.6860986 | 0.009945 | 0.04304649 | -2.8815639 |
| NDUFS8 | -0.5082778 | 6.42179105 | -2.8060164 | 0.00726273 | 0.03559294 | -2.6066602 |
| MPP1 | -0.5086432 | 6.75685289 | -3.4170797 | 0.00131123 | 0.01456888 | -1.0841296 |
| CSRP1 | -0.5108283 | 6.44559248 | -3.1697887 | 0.0026768 | 0.02135898 | -1.7232364 |
| HRAS | -0.5122099 | 6.49577038 | -2.7926186 | 0.00752515 | 0.03632806 | -2.6377948 |
| AKAP10 | -0.5166847 | 6.45580449 | -2.6014394 | 0.01235784 | 0.04875997 | -3.0704138 |
| HDHD3 | -0.518176 | 6.69326652 | -3.1205836 | 0.00307519 | 0.02276184 | -1.8468446 |
| WFS1 | -0.5202663 | 6.57625832 | -3.4335681 | 0.00124913 | 0.01403453 | -1.0405032 |
| ACADSB | -0.5240546 | 6.49465795 | -3.1289898 | 0.00300339 | 0.02246268 | -1.8258145 |
| NDUFA9 | -0.5294292 | 6.57883957 | -2.8413707 | 0.0066104 | 0.03386792 | -2.5240037 |
| SHTN1 | -0.5295036 | 6.94167443 | -3.0937094 | 0.0033157 | 0.02366803 | -1.913833 |
| CPNE8 | -0.5374097 | 6.57286494 | -2.7735041 | 0.00791474 | 0.03745116 | -2.6820325 |
| IDH3B | -0.5389991 | 6.44323259 | -2.6791886 | 0.01012439 | 0.04362279 | -2.8971428 |
| NDUFS6 | -0.539417 | 6.43934664 | -2.636939 | 0.01128738 | 0.04613462 | -2.9917619 |
| HSPA2 | -0.5394842 | 6.28860819 | -2.6783529 | 0.01014628 | 0.04363413 | -2.8990248 |
| JCAD | -0.5416129 | 6.55466378 | -3.2681072 | 0.00202201 | 0.01844702 | -1.472636 |
| FAM162A | -0.541761 | 6.48307346 | -3.1928514 | 0.00250731 | 0.02073285 | -1.664881 |
| MTMR2 | -0.5441247 | 6.42948005 | -2.6643685 | 0.01051918 | 0.04455759 | -2.9304569 |
| CLTA | -0.5451366 | 6.5409148 | -2.9678948 | 0.00469555 | 0.02798181 | -2.2224009 |
| ANXA11 | -0.5457466 | 6.74687395 | -4.8952975 | 1.1856E-05 | 0.00291583 | 3.20661059 |
| AP2M1 | -0.5470155 | 6.66720135 | -2.8957392 | 0.00571253 | 0.03106977 | -2.3955 |
| SMAP2 | -0.5470185 | 6.48631988 | -3.3374534 | 0.00165475 | 0.01634147 | -1.2930732 |
| LRRFIP1.iso4 | -0.5481635 | 6.37355597 | -3.0001245 | 0.00429827 | 0.02719949 | -2.1441608 |
| NUBPL | -0.5482727 | 6.24090797 | -2.7272825 | 0.00893511 | 0.04019194 | -2.7881163 |
| PTER | -0.5535949 | 6.6269949 | -3.4034592 | 0.00136473 | 0.01476331 | -1.1200755 |
| MXRA7.iso2 | -0.5558846 | 6.37729918 | -3.0691701 | 0.00355065 | 0.02433965 | -1.9746744 |
| C16orf70 | -0.5581742 | 6.60197506 | -2.7279347 | 0.0089199 | 0.04016492 | -2.7866284 |
| NDUFA6 | -0.5582286 | 6.58178795 | -2.9377154 | 0.00509841 | 0.0292313 | -2.2951504 |
| ARL15 | -0.5605086 | 6.55938767 | -3.1120702 | 0.00314954 | 0.02307362 | -1.8681059 |
| SLC9A1 | -0.5644366 | 6.49341277 | -2.6119875 | 0.01203046 | 0.04801761 | -3.0471254 |
| ATP6V1E1 | -0.5646132 | 6.48969642 | -2.6547938 | 0.01078171 | 0.04515661 | -2.9519089 |
| CYLD | -0.5669852 | 6.64124458 | -3.1062478 | 0.00320136 | 0.02321569 | -1.8826253 |
| ATP6V1B2 | -0.5678447 | 6.54982904 | -2.6069738 | 0.01218507 | 0.04833689 | -3.0582034 |
| NDUFS4 | -0.5721517 | 6.4094897 | -2.6856611 | 0.00995627 | 0.04304649 | -2.8825512 |
| DNAJB4 | -0.5761341 | 6.70267188 | -3.4120858 | 0.00133061 | 0.01462524 | -1.0973189 |
| NCEH1 | -0.5821178 | 6.51405508 | -3.1165141 | 0.00311052 | 0.02288814 | -1.8570124 |
| PALM2 | -0.5841081 | 6.407633 | -2.5877126 | 0.01279607 | 0.04982217 | -3.1006161 |
| C1orf198 | -0.5897057 | 6.17364011 | -2.951039 | 0.00491674 | 0.0285821 | -2.2630945 |
| MAP2K4 | -0.5901775 | 6.48482004 | -2.7199484 | 0.00910774 | 0.04064013 | -2.8048321 |
| COX7A2L | -0.5919581 | 6.40866751 | -2.7069157 | 0.0094221 | 0.04152519 | -2.8344568 |
| COX6B1 | -0.5924234 | 6.35305302 | -2.810703 | 0.00717296 | 0.0354125 | -2.5957447 |
| VTI1A | -0.5926757 | 6.44354477 | -3.0051445 | 0.0042393 | 0.02706131 | -2.1319239 |
| CCDC6 | -0.5939059 | 6.44076398 | -3.662761 | 0.00062929 | 0.01059182 | -0.4219358 |
| SBF1 | -0.5939877 | 6.42766432 | -2.6756353 | 0.01021778 | 0.04383674 | -2.9051423 |
| WDR37 | -0.5946418 | 6.39748562 | -2.9936671 | 0.00437526 | 0.02719949 | -2.1598817 |
| ATP5L | -0.5985361 | 6.82932983 | -2.6678834 | 0.01042429 | 0.04430855 | -2.9225679 |
| Sep.06 | -0.5997692 | 6.5246662 | -2.8757215 | 0.00602903 | 0.03203763 | -2.4430078 |
| AAK1 | -0.6052821 | 6.46558728 | -3.04461 | 0.00380137 | 0.02523004 | -2.0352518 |
| DOCK5 | -0.6064141 | 6.59037582 | -3.1280019 | 0.00301175 | 0.02248345 | -1.8282879 |
| PRKAB2 | -0.6064345 | 6.46274195 | -3.0745842 | 0.0034975 | 0.02419561 | -1.9612779 |
| NDUFA4 | -0.6080519 | 6.41670715 | -2.5960326 | 0.01252879 | 0.04916349 | -3.0823241 |
| HSPB8 | -0.6104313 | 6.18206659 | -2.7795659 | 0.00778922 | 0.03711649 | -2.6680265 |
| RAP2B | -0.6114201 | 6.62339087 | -4.2767987 | 9.158E-05 | 0.00500079 | 1.3321195 |
| NDUFA10 | -0.612022 | 6.46293169 | -2.9606568 | 0.00478937 | 0.02826755 | -2.239894 |
| NAPEPLD | -0.6188353 | 6.40418604 | -2.621992 | 0.0117273 | 0.04731521 | -3.0249732 |
| SIRT5 | -0.6214772 | 6.75249052 | -2.7668319 | 0.00805507 | 0.03780265 | -2.697424 |
| ADGRL1 | -0.6232706 | 6.44859818 | -2.6414338 | 0.01115807 | 0.04594338 | -2.9817477 |
| ME1 | -0.624069 | 6.53316333 | -2.8131649 | 0.00712621 | 0.03525117 | -2.5900056 |
| ATP6V1A | -0.6302385 | 6.4910486 | -2.9694826 | 0.0046752 | 0.02794772 | -2.2185595 |
| NDUFA12 | -0.6330072 | 6.49595911 | -2.9705896 | 0.00466106 | 0.02791443 | -2.2158807 |
| SRC | -0.6384181 | 6.30407668 | -2.9960463 | 0.00434674 | 0.02719949 | -2.154092 |
| SFXN3 | -0.6465891 | 6.59222204 | -2.9513448 | 0.00491264 | 0.0285821 | -2.2623576 |
| OPTN | -0.6467734 | 6.4851747 | -4.0580342 | 0.00018447 | 0.00626209 | 0.69278834 |
| ATP6V1D | -0.6499076 | 6.47032599 | -3.6643649 | 0.00062624 | 0.01059182 | -0.4175302 |
| HK1 | -0.6499706 | 6.44930563 | -2.6135423 | 0.01198288 | 0.04799857 | -3.0436868 |
| CISD3 | -0.6514335 | 6.56650062 | -4.4654679 | 4.9544E-05 | 0.00420786 | 1.89439273 |
| CISD1 | -0.6577124 | 6.48100197 | -3.0425985 | 0.00382263 | 0.02527069 | -2.0401989 |
| CAMK1D | -0.6578702 | 6.35664591 | -2.909839 | 0.00549896 | 0.03051036 | -2.361902 |
| NDUFS7 | -0.6707158 | 6.41827217 | -2.9744385 | 0.00461221 | 0.02784555 | -2.2065611 |
| PPP3R1 | -0.6733051 | 6.40328088 | -2.8715166 | 0.00609756 | 0.03226489 | -2.4529586 |
| CHCHD6 | -0.6763416 | 6.39945923 | -2.8194324 | 0.00700847 | 0.03509932 | -2.5753791 |
| AHNAK2 | -0.6790693 | 6.42857449 | -3.675814 | 0.00060483 | 0.01058137 | -0.3860527 |
| IDH3A | -0.6804001 | 6.36201227 | -2.8307476 | 0.00680046 | 0.03442134 | -2.5489157 |
| PALM | -0.6825758 | 6.36329426 | -2.6651206 | 0.01049881 | 0.04450893 | -2.9287696 |
| DKK3 | -0.6841971 | 6.42897753 | -2.9709511 | 0.00465645 | 0.02791443 | -2.2150057 |
| ATP1A2 | -0.6867869 | 6.31720847 | -2.6511977 | 0.01088185 | 0.04538065 | -2.9599512 |
| FTH1 | -0.6904954 | 6.67461435 | -3.179203 | 0.00260635 | 0.02109919 | -1.6994475 |
| PDXP | -0.692918 | 6.33503623 | -2.6549715 | 0.01077678 | 0.04515661 | -2.9515112 |
| IDH3G | -0.6933718 | 6.41664465 | -2.5882188 | 0.01277967 | 0.04981599 | -3.0995044 |
| GPR158 | -0.6973372 | 6.46506386 | -2.8192722 | 0.00701146 | 0.03509932 | -2.5757534 |
| ARRB1 | -0.6988094 | 6.58215061 | -4.2926071 | 8.7016E-05 | 0.00500079 | 1.37886361 |
| NCOA7.iso2 | -0.6999855 | 6.53983187 | -3.5269424 | 0.00094707 | 0.01241367 | -0.7911847 |
| ATP1B1 | -0.7004409 | 6.36628548 | -2.6745786 | 0.01024571 | 0.04388142 | -2.9075199 |
| SHC3 | -0.7010472 | 6.36068334 | -2.6943979 | 0.00973342 | 0.04241961 | -2.8628148 |
| NSF | -0.7024346 | 6.43991002 | -2.7963256 | 0.00745168 | 0.03611253 | -2.6291906 |
| DNAJB2 | -0.7033287 | 6.29620556 | -2.9286315 | 0.00522585 | 0.02972678 | -2.3169496 |
| DMXL2 | -0.7078029 | 6.51993987 | -2.8984459 | 0.00567094 | 0.03106977 | -2.3890589 |
| TPM2.iso3 | -0.7136296 | 6.51466155 | -2.8457212 | 0.006534 | 0.03354699 | -2.5137828 |
| MYO5A | -0.7145519 | 6.43391569 | -3.0462917 | 0.00378369 | 0.02521286 | -2.031114 |
| ELFN2 | -0.7182842 | 6.51062224 | -2.8264869 | 0.00687811 | 0.03463939 | -2.5588891 |
| CIT.iso4 | -0.7201347 | 6.27149952 | -2.6200985 | 0.01178413 | 0.04750622 | -3.0291705 |
| SPTBN4 | -0.7216182 | 6.4640684 | -3.2158799 | 0.00234821 | 0.01997768 | -1.6063481 |
| HPRT1 | -0.7237896 | 6.54483026 | -3.3011078 | 0.00183853 | 0.01738278 | -1.3874695 |
| NT5DC3 | -0.7260969 | 6.50073971 | -3.1537343 | 0.0028011 | 0.02169947 | -1.7637015 |
| ADD3 | -0.7263012 | 6.43749886 | -3.9275193 | 0.00027823 | 0.00737671 | 0.31857515 |
| KCNJ10 | -0.7272379 | 6.16950465 | -2.895972 | 0.00570895 | 0.03106977 | -2.3949462 |
| HPCAL1 | -0.730013 | 6.42231701 | -2.6813575 | 0.01006777 | 0.04341615 | -2.892256 |
| INPP5A | -0.7301462 | 6.40352545 | -3.0373474 | 0.00387864 | 0.02547294 | -2.0531039 |
| SCAI | -0.7306144 | 6.22983885 | -2.6994519 | 0.0096066 | 0.04211608 | -2.8513767 |
| REEP2 | -0.7321938 | 6.15076741 | -3.1472933 | 0.00285249 | 0.02188945 | -1.7798996 |
| PIP5K1C | -0.7328021 | 6.42399303 | -2.9274073 | 0.00524325 | 0.02972678 | -2.319884 |
| EHD3 | -0.7330028 | 6.35103791 | -3.0142577 | 0.00413417 | 0.02659349 | -2.1096746 |
| ROGDI | -0.7338317 | 6.50367936 | -3.0464221 | 0.00378232 | 0.02521286 | -2.030793 |
| CADM2 | -0.7409331 | 6.27825825 | -2.6473818 | 0.01098905 | 0.04566013 | -2.9684766 |
| NECAP1 | -0.7415074 | 6.33583446 | -2.6892976 | 0.00986295 | 0.04286491 | -2.8743419 |
| GDAP1 | -0.7432075 | 6.39755176 | -3.0494145 | 0.00375106 | 0.02506205 | -2.0234265 |
| IQSEC1 | -0.7439115 | 6.34469412 | -2.9273433 | 0.00524416 | 0.02972678 | -2.3200372 |
| ANK3 | -0.7445973 | 6.40182632 | -2.9678436 | 0.00469621 | 0.02798181 | -2.2225247 |
| ANLN | -0.7452568 | 5.91236086 | -2.6274363 | 0.01156527 | 0.04680292 | -3.0128923 |
| DDHD2 | -0.7460849 | 6.60904431 | -3.3821136 | 0.00145277 | 0.01516868 | -1.1762399 |
| CDK18 | -0.7470526 | 6.5655762 | -3.7967827 | 0.00041759 | 0.00895549 | -0.0503506 |
| SNTA1 | -0.7543359 | 6.49569469 | -3.165624 | 0.00270854 | 0.02140769 | -1.733746 |
| SH3GLB2 | -0.7610776 | 6.32104434 | -3.3356749 | 0.00166333 | 0.016343 | -1.2977068 |
| MAPRE3 | -0.7646624 | 6.31090343 | -2.7152215 | 0.00922062 | 0.04092519 | -2.8155888 |
| SPTBN2 | -0.7691078 | 6.26787195 | -2.7447768 | 0.00853543 | 0.03919643 | -2.7481139 |
| ASPA | -0.7691787 | 6.18244501 | -2.7261671 | 0.00896117 | 0.04023692 | -2.7906606 |
| LYRM9 | -0.7736809 | 6.3806009 | -2.9997039 | 0.00430325 | 0.02719949 | -2.1451854 |
| SLC25A12 | -0.7737724 | 6.39474347 | -2.9273697 | 0.00524378 | 0.02972678 | -2.3199739 |
| NECTIN1 | -0.7759699 | 6.35212856 | -2.7370203 | 0.00871056 | 0.03960854 | -2.7658723 |
| OPA1 | -0.7762696 | 6.28022492 | -3.1017275 | 0.00324215 | 0.02324302 | -1.8938856 |
| CDKL5 | -0.7779784 | 6.47710783 | -3.2040397 | 0.00242878 | 0.02039581 | -1.6364758 |
| ACBD7 | -0.7785499 | 6.27130226 | -2.9087373 | 0.00551537 | 0.03051036 | -2.364531 |
| NDUFV3 | -0.7807943 | 6.27390178 | -2.8771405 | 0.00600607 | 0.03198345 | -2.4396477 |
| CPNE5 | -0.7812662 | 6.33259648 | -2.7743537 | 0.00789704 | 0.03743563 | -2.6800708 |
| FAM171A1 | -0.7814019 | 6.37021165 | -3.6696274 | 0.00061631 | 0.01058137 | -0.4030682 |
| Sep.04 | -0.7840436 | 6.26786566 | -2.8990238 | 0.0056621 | 0.03106977 | -2.3876832 |
| CORO2A | -0.7846877 | 6.47393375 | -3.3023518 | 0.00183193 | 0.01738278 | -1.3842488 |
| PPFIA3 | -0.7847747 | 6.34428636 | -2.7265468 | 0.00895229 | 0.04023311 | -2.7897946 |
| ARFGAP1 | -0.7862354 | 6.28896113 | -2.8037409 | 0.0073067 | 0.03559294 | -2.6119556 |
| TINAGL1 | -0.7891583 | 6.51531059 | -3.242512 | 0.00217611 | 0.01924916 | -1.5383304 |
| GLS | -0.7908304 | 6.48243535 | -2.8724715 | 0.00608193 | 0.03221623 | -2.4506996 |
| CNP | -0.7987204 | 6.07296701 | -2.6414819 | 0.01115669 | 0.04594338 | -2.9816403 |
| ATPIF1 | -0.7991494 | 6.37875903 | -3.3801214 | 0.00146126 | 0.01520977 | -1.1814711 |
| GPM6B | -0.8003515 | 6.16486243 | -2.5862233 | 0.01284446 | 0.04993298 | -3.1038858 |
| SYNPO | -0.8019788 | 6.44927303 | -3.3853454 | 0.0014391 | 0.01514988 | -1.1677496 |
| CDH13 | -0.8048243 | 6.27071891 | -3.051773 | 0.00372658 | 0.02501127 | -2.0176171 |
| ME3 | -0.806975 | 6.34379495 | -2.8566396 | 0.00634584 | 0.03291823 | -2.4880842 |
| FAM171B | -0.8091542 | 6.27905969 | -2.9223409 | 0.00531583 | 0.02987349 | -2.3320188 |
| TPD52L1 | -0.809717 | 6.41662702 | -2.8328229 | 0.00676294 | 0.03429386 | -2.5440541 |
| ADD1 | -0.8101127 | 6.33767087 | -3.9312634 | 0.00027499 | 0.00736879 | 0.32922997 |
| C2CD2L | -0.8116649 | 6.5596161 | -3.0348354 | 0.0039057 | 0.02561712 | -2.0592721 |
| SYT2 | -0.8194187 | 6.09147801 | -2.8446247 | 0.00655318 | 0.03361103 | -2.5163601 |
| ATP6V0D1 | -0.8265444 | 6.35034817 | -2.9846387 | 0.00448506 | 0.02738557 | -2.181824 |
| EDIL3 | -0.832458 | 6.48321057 | -3.0226283 | 0.00403976 | 0.02615409 | -2.0891991 |
| DNAJC5 | -0.8375118 | 6.27991327 | -3.134153 | 0.00296008 | 0.02223203 | -1.8128797 |
| REPS2 | -0.8410119 | 6.4333129 | -3.565124 | 0.00084487 | 0.01193402 | -0.6881543 |
| CEP170B | -0.8413206 | 6.31680351 | -2.9136511 | 0.0054425 | 0.03030265 | -2.352799 |
| WDR7 | -0.8424424 | 6.39825336 | -3.4713693 | 0.00111718 | 0.01320322 | -0.9400296 |
| STXBP6 | -0.8437996 | 6.21699419 | -3.2046442 | 0.0024246 | 0.02039581 | -1.6349394 |
| UNC13A | -0.8473078 | 6.48069232 | -3.2746062 | 0.00198455 | 0.01824698 | -1.4559047 |
| CAVIN1 | -0.8537466 | 6.34971896 | -4.3728848 | 6.7052E-05 | 0.00456851 | 1.61729893 |
| GDA | -0.8668619 | 6.20892889 | -2.619347 | 0.01180676 | 0.04750622 | -3.0308358 |
| RYR2 | -0.8685455 | 6.26425589 | -2.8053959 | 0.0072747 | 0.03559294 | -2.6081046 |
| ATP6V0A1 | -0.8801261 | 6.31731946 | -2.8817868 | 0.00593145 | 0.03175482 | -2.4286369 |
| NRXN3 | -0.8813825 | 6.20349845 | -2.7437313 | 0.00855885 | 0.03923914 | -2.7505096 |
| ATP6V1H | -0.8828208 | 6.44221721 | -3.7510544 | 0.00048066 | 0.00926378 | -0.1779115 |
| ADCY5 | -0.8835963 | 6.32710914 | -3.9792476 | 0.00023656 | 0.00693228 | 0.46620808 |
| LMO7 | -0.8838119 | 6.53612037 | -3.1478846 | 0.00284773 | 0.02188649 | -1.7784133 |
| KIAA0513 | -0.8842316 | 6.27865529 | -2.7670528 | 0.00805039 | 0.03780265 | -2.6969147 |
| LAMP5 | -0.8843549 | 6.23877818 | -2.8325184 | 0.00676843 | 0.03429386 | -2.5447676 |
| CALB1 | -0.8856429 | 5.73845162 | -2.88541 | 0.00587387 | 0.03161541 | -2.4200424 |
| RGS7 | -0.8880898 | 6.17525122 | -2.5884517 | 0.01277212 | 0.04981599 | -3.098993 |
| HPCAL4 | -0.8885948 | 6.13500254 | -2.6689961 | 0.01039442 | 0.04430855 | -2.9200689 |
| DLG2 | -0.8886243 | 6.11478593 | -2.6457104 | 0.01103631 | 0.04578057 | -2.9722081 |
| MACROD2.iso1 | -0.8920289 | 6.21363538 | -2.7851843 | 0.00767452 | 0.03688619 | -2.6550257 |
| AKR1C3 | -0.8932832 | 6.22493794 | -4.1451459 | 0.00013981 | 0.00577125 | 0.94563249 |
| GPM6A | -0.898274 | 6.27595317 | -2.7797079 | 0.0077863 | 0.03711649 | -2.6676982 |
| MYO1D | -0.90055 | 6.46019563 | -4.1334839 | 0.00014512 | 0.00577125 | 0.91164619 |
| WASF1 | -0.9039751 | 6.2247449 | -2.9291005 | 0.0052192 | 0.02972678 | -2.3158251 |
| CPNE7 | -0.9060491 | 6.04481629 | -2.9645755 | 0.00473836 | 0.02813261 | -2.2304268 |
| GNB5 | -0.9065134 | 6.12543287 | -2.6244157 | 0.01165491 | 0.04709901 | -3.0195973 |
| PDK2 | -0.9130231 | 6.34649296 | -3.4927626 | 0.0010485 | 0.01278355 | -0.882889 |
| CNN1 | -0.9201588 | 6.10825435 | -2.9809269 | 0.00453095 | 0.02755411 | -2.1908323 |
| GNAO1 | -0.9201774 | 6.1523228 | -2.6782866 | 0.01014802 | 0.04363413 | -2.8991743 |
| ATL1 | -0.9202733 | 6.31921178 | -3.5111627 | 0.00099268 | 0.01259316 | -0.8335841 |
| MBLAC2 | -0.9235193 | 6.27206234 | -3.2374867 | 0.00220764 | 0.01934001 | -1.5511916 |
| SRCIN1 | -0.9246834 | 6.23322863 | -3.2123711 | 0.00237181 | 0.02014435 | -1.6152834 |
| OXR1 | -0.9275895 | 6.22145293 | -3.0801647 | 0.00344351 | 0.02408712 | -1.9474538 |
| IQSEC2 | -0.9306226 | 6.44866627 | -3.2521055 | 0.00211709 | 0.01893409 | -1.5137441 |
| ERC2 | -0.9323434 | 6.15434829 | -2.8820768 | 0.00592683 | 0.03175482 | -2.4279494 |
| PDE1B | -0.933216 | 6.20563606 | -2.8036247 | 0.00730895 | 0.03559294 | -2.6122259 |
| ACTR3B | -0.934272 | 6.21115833 | -2.8375207 | 0.00667871 | 0.03408045 | -2.5330397 |
| DMTN | -0.9345269 | 6.18890787 | -2.9934439 | 0.00437794 | 0.02719949 | -2.1604245 |
| TMEM65 | -0.9353131 | 6.22737266 | -3.6632256 | 0.00062841 | 0.01059182 | -0.4206597 |
| ITPKA | -0.936146 | 6.19351818 | -2.8166877 | 0.00705981 | 0.03515867 | -2.5817873 |
| IGSF8 | -0.936743 | 6.06068685 | -2.7148198 | 0.00923027 | 0.04093178 | -2.8165023 |
| GJA1 | -0.9378746 | 6.15327405 | -3.5602316 | 0.00085735 | 0.01193402 | -0.7013905 |
| GABBR2 | -0.9394584 | 5.99630577 | -2.6898346 | 0.00984924 | 0.04284247 | -2.8731289 |
| CDC42.iso1 | -0.9398449 | 6.22092389 | -2.863021 | 0.00623821 | 0.03266424 | -2.4730325 |
| TUBB4A | -0.9424949 | 6.15191981 | -2.7202741 | 0.00910001 | 0.04064013 | -2.8040907 |
| MBP.iso4 | -0.9545026 | 5.737895 | -2.7567213 | 0.00827209 | 0.03838097 | -2.720697 |
| RBP7 | -0.9554823 | 6.30385101 | -3.251669 | 0.00211974 | 0.01893409 | -1.5148638 |
| GSTM5 | -0.9558058 | 6.30109662 | -2.9945583 | 0.00436456 | 0.02719949 | -2.1577133 |
| PPP1R14A | -0.9569466 | 6.01516455 | -3.1259149 | 0.00302947 | 0.02248345 | -1.8335113 |
| PCLO | -0.9583864 | 5.97529763 | -2.6047101 | 0.01225547 | 0.04850882 | -3.0632001 |
| CD200 | -0.960423 | 6.10639292 | -2.990867 | 0.00440904 | 0.0273099 | -2.1666917 |
| PPM1H | -0.9605584 | 6.1924926 | -3.0514717 | 0.0037297 | 0.02501127 | -2.0183595 |
| PVALB | -0.9689539 | 6.35663875 | -2.8398552 | 0.00663721 | 0.03393783 | -2.5275618 |
| DNAJC6 | -0.9696883 | 6.2467787 | -3.0601086 | 0.00364129 | 0.02485899 | -1.9970614 |
| SLC7A14 | -0.9711097 | 6.08950789 | -2.6983662 | 0.00963372 | 0.04219803 | -2.8538353 |
| GRM5 | -0.9711344 | 6.17135881 | -2.9870319 | 0.00445571 | 0.02736001 | -2.1760118 |
| SCAMP5 | -0.9728124 | 6.11916096 | -2.7038948 | 0.00949638 | 0.04170583 | -2.841309 |
| SULT4A1 | -0.97476 | 6.17579818 | -2.7740199 | 0.00790399 | 0.03743563 | -2.6808417 |
| KCTD8 | -0.9769203 | 5.85244645 | -2.6340436 | 0.01137139 | 0.04628924 | -2.998206 |
| CAMKK1 | -0.9775083 | 6.14390226 | -2.8759006 | 0.00602613 | 0.03203763 | -2.4425837 |
| NIPSNAP3B | -0.9794308 | 6.22793095 | -3.096567 | 0.00328931 | 0.02351315 | -1.9067277 |
| SNAP91 | -0.9795321 | 6.07681127 | -2.6195005 | 0.01180214 | 0.04750622 | -3.0304957 |
| ICAM5 | -0.9851483 | 6.16324645 | -2.6859942 | 0.00994769 | 0.04304649 | -2.8817995 |
| CADPS | -0.9866503 | 6.27263843 | -2.7400999 | 0.00864063 | 0.03936201 | -2.7588258 |
| SHANK3 | -0.9883811 | 6.06859558 | -2.8514085 | 0.00643535 | 0.03317649 | -2.5004051 |
| FBXO2 | -0.9898331 | 6.25232516 | -3.041568 | 0.00383356 | 0.02529411 | -2.0427327 |
| TUBA4A | -0.9928446 | 6.16467345 | -3.0064526 | 0.00422406 | 0.02706131 | -2.1287331 |
| GNAZ | -0.999213 | 6.24920875 | -3.109088 | 0.00317599 | 0.02314848 | -1.8755449 |
| CHGA | -1.0041931 | 6.13663465 | -3.2676412 | 0.00202472 | 0.01844702 | -1.4738349 |
| MBP | -1.005941 | 5.51673573 | -2.7349642 | 0.00875753 | 0.03978603 | -2.7705737 |
| PKM.isom1 | -1.0095721 | 6.44819959 | -3.630403 | 0.00069411 | 0.01111237 | -0.5105945 |
| SLC1A2 | -1.0101439 | 6.02503586 | -2.9034683 | 0.00559452 | 0.03080674 | -2.3770962 |
| DPP10 | -1.0136963 | 5.91315235 | -2.6628521 | 0.01056037 | 0.04469426 | -2.9338582 |
| SYT7 | -1.0144561 | 6.16922125 | -2.7458606 | 0.00851122 | 0.03916413 | -2.7456297 |
| NEBL | -1.0166922 | 6.08833278 | -3.5805963 | 0.00080653 | 0.01180484 | -0.6462287 |
| AK5 | -1.0189107 | 6.39700527 | -3.2980388 | 0.0018549 | 0.0174745 | -1.3954116 |
| PPP3CB | -1.0198293 | 6.10832467 | -3.3651208 | 0.00152669 | 0.01561279 | -1.2208024 |
| HSPA12A | -1.0206086 | 6.08817127 | -3.0252457 | 0.00401066 | 0.02603294 | -2.0827891 |
| RASAL1 | -1.0262866 | 6.0461142 | -2.6191545 | 0.01181256 | 0.04750622 | -3.0312624 |
| DNM1.iso2 | -1.0288562 | 6.14883721 | -2.8641848 | 0.00621877 | 0.03259649 | -2.4702851 |
| STXBP1.iso2 | -1.0290752 | 5.92252111 | -2.6422741 | 0.01113405 | 0.04594338 | -2.979874 |
| RAB3C | -1.0359733 | 5.98516827 | -2.7217838 | 0.00906425 | 0.04053872 | -2.800652 |
| GRM3 | -1.0369369 | 6.10705425 | -3.495548 | 0.00103986 | 0.01278355 | -0.8754345 |
| PSD3 | -1.0407806 | 6.12993185 | -2.9217958 | 0.00532369 | 0.02987349 | -2.3333235 |
| SYNPR | -1.0577802 | 6.07821813 | -2.6228834 | 0.01170063 | 0.04724565 | -3.0229965 |
| SNAP25 | -1.0585284 | 6.00891444 | -2.9382841 | 0.00509053 | 0.0292313 | -2.293784 |
| RASGRF2 | -1.059525 | 6.46966922 | -3.6507604 | 0.00065262 | 0.01079156 | -0.4548658 |
| BRSK2.iso4 | -1.0595611 | 6.14359597 | -3.2005442 | 0.00245306 | 0.02052043 | -1.6453568 |
| ERMN | -1.0614459 | 5.99009861 | -3.5744183 | 0.00082163 | 0.01183103 | -0.6629813 |
| PRELP | -1.0616652 | 5.90779086 | -2.6114822 | 0.01204596 | 0.04802093 | -3.0482426 |
| BSN | -1.0640463 | 5.96975647 | -2.7245508 | 0.00899905 | 0.04037085 | -2.7943461 |
| ADAP1 | -1.0646935 | 6.26679992 | -3.2545205 | 0.00210247 | 0.01888082 | -1.507548 |
| GRIN2B | -1.0715248 | 6.10445308 | -2.7977097 | 0.00742442 | 0.03605014 | -2.6259761 |
| SEPT4.iso5 | -1.0790858 | 6.41826944 | -3.153953 | 0.00279937 | 0.02169947 | -1.7631513 |
| SLC8A2 | -1.0795204 | 6.04880222 | -3.1029125 | 0.00323141 | 0.02324302 | -1.8909346 |
| PRNP | -1.0895181 | 6.3677151 | -4.1231309 | 0.00014999 | 0.00582631 | 0.88150968 |
| SNCB | -1.0912758 | 6.04155713 | -2.8601684 | 0.00628611 | 0.03282923 | -2.4797636 |
| OGDHL | -1.0921732 | 6.00286658 | -3.1419775 | 0.00289556 | 0.02201769 | -1.7932521 |
| NCDN | -1.1075541 | 6.32120378 | -2.6422853 | 0.01113373 | 0.04594338 | -2.9798492 |
| PACSIN1 | -1.1081525 | 5.83162471 | -2.7800115 | 0.00778006 | 0.03711649 | -2.666996 |
| TPRG1L | -1.1096752 | 6.16323309 | -3.5433235 | 0.00090185 | 0.01221401 | -0.7470571 |
| MAG | -1.1124745 | 5.42569143 | -2.7668597 | 0.00805448 | 0.03780265 | -2.6973599 |
| PHF24 | -1.1183411 | 6.14791366 | -2.9192706 | 0.00536027 | 0.03001152 | -2.3393658 |
| CAMK2A | -1.1261198 | 5.93147757 | -2.7790966 | 0.00779887 | 0.03711649 | -2.6691117 |
| SGIP1 | -1.1287873 | 6.00552261 | -2.9879775 | 0.00444416 | 0.02735833 | -2.1737147 |
| STXBP5 | -1.1304812 | 6.26610876 | -3.3303122 | 0.00168943 | 0.0164383 | -1.3116693 |
| NUF2 | -1.1311228 | 5.99226928 | -3.2959968 | 0.00186587 | 0.01751842 | -1.4006937 |
| PRKCG | -1.1327881 | 5.99434541 | -3.0547093 | 0.00369633 | 0.02499635 | -2.0103804 |
| SLC17A7 | -1.1332289 | 5.93514593 | -2.9070716 | 0.00554028 | 0.03060898 | -2.3685051 |
| PHYHIP | -1.1480691 | 5.97014321 | -2.6672161 | 0.01044225 | 0.04430855 | -2.9240663 |
| SYT12 | -1.1485107 | 6.01516749 | -3.2837692 | 0.00193287 | 0.01792213 | -1.4322809 |
| CPNE6 | -1.1493786 | 6.11153318 | -3.5321117 | 0.00093257 | 0.01241367 | -0.7772717 |
| NEFH | -1.1519085 | 5.66766994 | -2.7557026 | 0.00829426 | 0.03843492 | -2.7230386 |
| TPM1.iso7 | -1.1546805 | 6.1877153 | -5.69706 | 7.5957E-07 | 0.00190311 | 5.73394898 |
| PLCXD3 | -1.1571397 | 6.93053949 | -3.4985731 | 0.00103056 | 0.01278355 | -0.8673349 |
| PRKCB | -1.1613944 | 6.05393551 | -3.5514227 | 0.00088027 | 0.01203493 | -0.7251973 |
| SH3GL2 | -1.1624208 | 6.08074663 | -3.313808 | 0.00177222 | 0.01704526 | -1.3545553 |
| CAMKV | -1.1662488 | 5.9239646 | -2.9306135 | 0.00519779 | 0.02969913 | -2.3121972 |
| TUBA8 | -1.1664116 | 6.08614615 | -2.8559604 | 0.0063574 | 0.03292411 | -2.4896846 |
| CPLX2 | -1.167365 | 5.94515201 | -2.7286642 | 0.00890292 | 0.04016492 | -2.7849635 |
| SYNGAP1 | -1.1744002 | 5.96164108 | -3.1337212 | 0.00296368 | 0.02223203 | -1.8139621 |
| CAVIN3 | -1.1790175 | 5.92530679 | -4.5403952 | 3.8723E-05 | 0.00388085 | 2.12020924 |
| SYT1 | -1.1861522 | 5.82670211 | -2.7571711 | 0.00826232 | 0.03837118 | -2.7196629 |
| HCN2 | -1.1910699 | 6.12706746 | -3.5282262 | 0.00094345 | 0.01241367 | -0.7877305 |
| SNCG | -1.2010407 | 5.83945761 | -2.8858179 | 0.00586742 | 0.03161464 | -2.4190743 |
| BSCL2 | -1.204103 | 6.13919964 | -3.5583759 | 0.00086213 | 0.01193402 | -0.7064083 |
| RAB3A | -1.2082626 | 5.84446808 | -2.819682 | 0.00700382 | 0.03509932 | -2.5747964 |
| GRIA1 | -1.2104126 | 6.08043835 | -3.6846859 | 0.00058872 | 0.01044945 | -0.361625 |
| SLC12A5 | -1.2196916 | 5.90104062 | -2.8834318 | 0.00590524 | 0.03171615 | -2.4247357 |
| SV2A | -1.2268855 | 5.73196169 | -2.6677555 | 0.01042773 | 0.04430855 | -2.9228552 |
| INA | -1.2287731 | 5.77721697 | -2.9881927 | 0.00444153 | 0.02735833 | -2.1731918 |
| SYN2 | -1.231312 | 5.81441383 | -2.7154396 | 0.00921539 | 0.04092519 | -2.8150928 |
| DLG4 | -1.2381814 | 6.01827202 | -3.376182 | 0.00147818 | 0.01520977 | -1.19181 |
| SNPH | -1.2474736 | 6.18076062 | -4.1852347 | 0.00012298 | 0.00567565 | 1.06277786 |
| SCG5.iso2 | -1.2521085 | 6.2701791 | -2.7215902 | 0.00906883 | 0.04053872 | -2.8010931 |
| FGF12 | -1.2655435 | 5.90935173 | -2.9932429 | 0.00438036 | 0.02719949 | -2.1609136 |
| SV2B | -1.2851321 | 5.87257907 | -3.3422316 | 0.00163193 | 0.01625769 | -1.2806173 |
| TPPP | -1.2902823 | 5.85855824 | -3.1674472 | 0.0026946 | 0.02139879 | -1.7291463 |
| MYH11 | -1.3144335 | 5.95147355 | -4.3284738 | 7.7467E-05 | 0.00479245 | 1.48517606 |
| RTN1.iso | -1.3148265 | 5.62333478 | -2.8494671 | 0.00646887 | 0.03328764 | -2.5049737 |
| PYGM | -1.3190699 | 6.07180795 | -3.7714693 | 0.00045145 | 0.00898474 | -0.121061 |
| DLGAP3 | -1.3427885 | 6.13951776 | -3.8253137 | 0.00038237 | 0.00863088 | 0.02963315 |
| STX1A | -1.3439885 | 5.77087615 | -3.317138 | 0.00175521 | 0.01691415 | -1.3459128 |
| ATP2B4.isoxa | -1.3465247 | 5.80995072 | -2.8978521 | 0.00568004 | 0.03106977 | -2.3904721 |
| SYP | -1.3675354 | 5.78714186 | -3.1339851 | 0.00296148 | 0.02223203 | -1.8133006 |
| KCTD16 | -1.3727201 | 5.81250911 | -3.1458738 | 0.00286393 | 0.02194366 | -1.7834665 |
| SH3GL3 | -1.4073153 | 5.82834133 | -3.4939736 | 0.00104474 | 0.01278355 | -0.8796483 |
| RTN4.isoc | -1.4451978 | 6.02542185 | -3.3495672 | 0.00159748 | 0.01600993 | -1.2614742 |
| GABRA1 | -1.4468309 | 5.91550692 | -3.4751992 | 0.00110457 | 0.01314731 | -0.9298147 |
| PCSK1N | -1.486737 | 5.77449639 | -3.464616 | 0.00113973 | 0.01337516 | -0.9580258 |
| AOC3 | -1.555612 | 5.91015135 | -4.5840356 | 3.3526E-05 | 0.0037324 | 2.25234683 |
| COL1A1 | -1.5986515 | 5.42337882 | -3.6974813 | 0.00056622 | 0.0102901 | -0.3263398 |
| COL1A2 | -1.6479348 | 5.35045487 | -3.5723623 | 0.00082672 | 0.01186791 | -0.6685529 |
| OGN | -2.0063636 | 4.73352504 | -3.6003987 | 0.00075989 | 0.01153877 | -0.5924247 |
| logFC: log2 fold change; AveExpr: Average expression; t: moderated t-statistic;P.Value p value; adj.P.Val: adjusted p value (Benjamini-Hochberg); B: Bayes factor  Supplementary Table 3B. Differential abundant plasma proteins in pGBM vs reGBM (adjusted p value < 0.05 & absolute FC < 0.05)   \| Protein ID \| logFC \| AveExpr \| t \| P.Value \| adj.P.Val \| B \| \| --- \| --- \| --- \| --- \| --- \| --- \| --- \| \| PI16 \| -0.5559 \| 6.39590267 \| -5.3702232 \| 2.57E-06 \| 4.23E-04 \| 4.63214397 \| \| HBG1 \| -0.6791056 \| 6.13534342 \| -2.9227624 \| 0.00538448 \| 0.03666841 \| -2.4733143 \|   logFC: log2 fold change; AveExpr: Average expression; t: moderated t-statistic;P.Value p value; adj.P.Val: adjusted p value (Benjamini-Hochberg); B: Bayes factor | | | | | | |

**Supplementary Table 4 Representative pathways for module 'turqoise' in plasma pGBM and reGBM**

**Supplementary Table 4A Primary Plasma Representative Top 12 Pathways**

| Reactome Term ID | Term description | Observed gene count | Background gene count | Strength | Signal | False discovery rate | Matching proteins in your network (labels) |
| --- | --- | --- | --- | --- | --- | --- | --- |
| HSA-114608 | Platelet degranulation | 13 | 126 | 1.86 | 5.01 | 4.64E-18 | VCL,PFN1,TUBA4A,ITGA2B,FERMT3,TLN1,TAGLN2,FLNA,CAP1,ACTN1,WDR1,CFL1,ITGB3 |
| HSA-76002 | Platelet activation, signaling and aggregation | 15 | 260 | 1.61 | 4.02 | 4.64E-18 | VCL,PFN1,TUBA4A,RAP1B,ITGA2B,FERMT3,TLN1,TAGLN2,FLNA,CAP1,ACTN1,YWHAZ,WDR1,CFL1,ITGB3 |
| HSA-109582 | Hemostasis | 17 | 607 | 1.29 | 2.62 | 2.27E-16 | VCL,PFN1,TUBA4A,RAP1B,ITGA2B,FERMT3,TLN1,TUBA1B,TAGLN2,FLNA,CAP1,ACTN1,YWHAZ,WDR1,CFL1,ITGB3,ACTB |
| HSA-6802948 | Signaling by high-kinase activity BRAF mutants | 6 | 36 | 2.07 | 2.97 | 1.07E-08 | VCL,RAP1B,ITGA2B,TLN1,ITGB3,ACTB |
| HSA-5674135 | MAP2K and MAPK activation | 6 | 40 | 2.02 | 2.87 | 1.58E-08 | VCL,RAP1B,ITGA2B,TLN1,ITGB3,ACTB |
| HSA-9656223 | Signaling by RAF1 mutants | 6 | 41 | 2.01 | 2.86 | 1.58E-08 | VCL,RAP1B,ITGA2B,TLN1,ITGB3,ACTB |
| HSA-6802946 | Signaling by moderate kinase activity BRAF mutants | 6 | 45 | 1.97 | 2.78 | 2.27E-08 | VCL,RAP1B,ITGA2B,TLN1,ITGB3,ACTB |
| HSA-6802955 | Paradoxical activation of RAF signaling by kinase inactive BRAF | 6 | 45 | 1.97 | 2.78 | 2.27E-08 | VCL,RAP1B,ITGA2B,TLN1,ITGB3,ACTB |
| HSA-9649948 | Signaling downstream of RAS mutants | 6 | 45 | 1.97 | 2.78 | 2.27E-08 | VCL,RAP1B,ITGA2B,TLN1,ITGB3,ACTB |
| HSA-422475 | Axon guidance | 11 | 551 | 1.15 | 1.58 | 2.40E-08 | MYH9,PFN1,TUBA4A,ITGA2B,TLN1,TUBA1B,CAP1,CFL1,MYL6,ITGB3,ACTB |
| HSA-195258 | RHO GTPase Effectors | 9 | 292 | 1.34 | 1.9 | 3.47E-08 | MYH9,PFN1,TUBA4A,TUBA1B,FLNA,YWHAZ,CFL1,MYL6,ACTB |
| HSA-6802952 | Signaling by BRAF and RAF1 fusions | 6 | 65 | 1.81 | 2.45 | 9.43E-08 | VCL,RAP1B,ITGA2B,TLN1,ITGB3,ACTB |

**Supplementary Table 4B Representative pathways for module 'turqoise' in plasma pGBM and reGBM**

| Reactome Term ID | Term description | Observed gene count | Background gene count | Strength | Signal | False discovery rate | Matching proteins in your network (labels) |
| --- | --- | --- | --- | --- | --- | --- | --- |
| HSA-114608 | Platelet degranulation | 18 | 126 | 1.93 | 6.96 | 2.48E-27 | VCL,PFN1,TUBA4A,THBS1,ITGA2B,FERMT3,PPBP,PF4,TLN1,HSPA5,TAGLN2,FLNA,CAP1,ACTN1,MMRN1,WDR1,CFL1,ITGB3 |
| HSA-76002 | Platelet activation, signaling and aggregation | 20 | 260 | 1.66 | 5.27 | 2.04E-26 | VCL,PFN1,TUBA4A,RAP1B,THBS1,ITGA2B,FERMT3,PPBP,PF4,TLN1,HSPA5,TAGLN2,FLNA,CAP1,ACTN1,MMRN1,YWHAZ,WDR1,CFL1,ITGB3 |
| HSA-109582 | Hemostasis | 22 | 607 | 1.34 | 3.23 | 6.62E-23 | VCL,PFN1,TUBA4A,RAP1B,THBS1,ITGA2B,FERMT3,PPBP,PF4,TLN1,HSPA5,TUBA1B,TAGLN2,FLNA,CAP1,ACTN1,MMRN1,YWHAZ,WDR1,CFL1,ITGB3,ACTB |
| HSA-6802948 | Signaling by high-kinase activity BRAF mutants | 6 | 36 | 2 | 2.67 | 3.10E-08 | VCL,RAP1B,ITGA2B,TLN1,ITGB3,ACTB |
| HSA-5674135 | MAP2K and MAPK activation | 6 | 40 | 1.95 | 2.58 | 4.60E-08 | VCL,RAP1B,ITGA2B,TLN1,ITGB3,ACTB |
| HSA-9656223 | Signaling by RAF1 mutants | 6 | 41 | 1.94 | 2.57 | 4.60E-08 | VCL,RAP1B,ITGA2B,TLN1,ITGB3,ACTB |
| HSA-162582 | Signal Transduction | 20 | 2540 | 0.67 | 0.8 | 5.12E-08 | VCL,MYH9,PFN1,TUBA4A,RAP1B,THBS1,ITGA2B,PPBP,PF4,TLN1,TUBA1B,FLNA,TFRC,ACTN1,YWHAZ,CFL1,MYL6,ITGB3,TPM4,ACTB |
| HSA-6802946 | Signaling by moderate kinase activity BRAF mutants | 6 | 45 | 1.9 | 2.51 | 5.86E-08 | VCL,RAP1B,ITGA2B,TLN1,ITGB3,ACTB |
| HSA-6802955 | Paradoxical activation of RAF signaling by kinase inactive BRAF | 6 | 45 | 1.9 | 2.51 | 5.86E-08 | VCL,RAP1B,ITGA2B,TLN1,ITGB3,ACTB |
| HSA-9649948 | Signaling downstream of RAS mutants | 6 | 45 | 1.9 | 2.51 | 5.86E-08 | VCL,RAP1B,ITGA2B,TLN1,ITGB3,ACTB |
| HSA-194315 | Signaling by Rho GTPases | 12 | 672 | 1.03 | 1.33 | 8.74E-08 | MYH9,PFN1,TUBA4A,TUBA1B,FLNA,TFRC,ACTN1,YWHAZ,CFL1,MYL6,TPM4,ACTB |
| HSA-422475 | Axon guidance | 11 | 551 | 1.08 | 1.38 | 1.51E-07 | MYH9,PFN1,TUBA4A,ITGA2B,TLN1,TUBA1B,CAP1,CFL1,MYL6,ITGB3,ACTB |

| Supplementary Table 5A. Differential abundant plasma proteins in ALL_GBM (p value < 0.05) | | | | | | |
| --- | --- | --- | --- | --- | --- | --- |
| Protein ID | **logFC** | **AveExpr** | **t** | **P.Value** | **adj.P.Val** | **B** |
| SAA1 | 1.16782494 | 5.95195677 | 5.08856483 | 1.34E-06 | 2.29E-05 | 4.89025537 |
| SAA2 | 0.75446917 | 5.91490168 | 2.16763826 | 0.03214842 | 0.07949487 | -4.4745312 |
| HP | 0.66201607 | 6.48131445 | 6.79428671 | 4.38E-10 | 4.80E-08 | 12.6395187 |
| APOE | 0.47906111 | 6.52995022 | 4.28396863 | 3.71E-05 | 3.03E-04 | 1.72556386 |
| COL18A1 | 0.46584791 | 6.48328743 | 3.74181239 | 2.81E-04 | 0.00175917 | -0.1806863 |
| SERPINA3 | 0.45581638 | 6.66109933 | 6.07522144 | 1.48E-08 | 6.97E-07 | 9.22746324 |
| VWF | 0.45355115 | 6.59284889 | 3.68849519 | 3.39E-04 | 0.00206833 | -0.3573349 |
| F9 | 0.41901078 | 6.59696217 | 9.22921537 | 1.12E-15 | 3.69E-13 | 25.1739514 |
| CST3 | 0.40797166 | 6.63162341 | 6.43486311 | 2.60E-09 | 1.77E-07 | 10.911086 |
| APOC3 | 0.38384162 | 6.55251251 | 3.94890523 | 1.32E-04 | 9.08E-04 | 0.52421576 |
| SAA4 | 0.38068815 | 6.57545051 | 4.93475479 | 2.60E-06 | 3.56E-05 | 4.25692322 |
| AZGP1 | 0.37749204 | 6.59599794 | 5.07954912 | 1.39E-06 | 2.29E-05 | 4.85278527 |
| PRG4 | 0.37402657 | 6.54195465 | 4.31700489 | 3.26E-05 | 2.90E-04 | 1.84793323 |
| S100A8 | 0.37024843 | 6.27977852 | 2.14673388 | 0.03381412 | 0.08240625 | -4.5177227 |
| ORM1 | 0.36782084 | 6.62935907 | 4.95823309 | 2.35E-06 | 3.36E-05 | 4.35277698 |
| MRC1 | 0.3503865 | 6.64785061 | 4.68727739 | 7.34E-06 | 8.62E-05 | 3.26514686 |
| SERPINA4 | 0.34565762 | 6.62151591 | 5.92510306 | 3.02E-08 | 1.24E-06 | 8.5395966 |
| S100A9 | 0.32331907 | 6.36611246 | 2.12186512 | 0.03589255 | 0.08556992 | -4.5686004 |
| HPX | 0.32000001 | 6.72479611 | 6.16473152 | 9.66E-09 | 5.30E-07 | 9.64190572 |
| INHBC | 0.31744588 | 6.66476466 | 4.25811754 | 4.10E-05 | 3.21E-04 | 1.63028932 |
| PROC | 0.30834481 | 6.62080569 | 5.21326828 | 7.77E-07 | 1.60E-05 | 5.41286138 |
| APCS | 0.29718711 | 6.63364349 | 4.95838527 | 2.35E-06 | 3.36E-05 | 4.35339925 |
| AGT | 0.28692601 | 6.57637551 | 4.32583223 | 3.15E-05 | 2.88E-04 | 1.88074655 |
| APOL1 | 0.28151226 | 6.6881171 | 4.27896937 | 3.78E-05 | 3.03E-04 | 1.70710605 |
| CPB2 | 0.27646349 | 6.71242368 | 5.2843223 | 5.68E-07 | 1.41E-05 | 5.714181 |
| APOD | 0.25723218 | 6.52540635 | 2.35731906 | 0.02001569 | 0.05226318 | -4.0650615 |
| OAF | 0.25567856 | 6.69004444 | 3.14474691 | 0.0020928 | 0.00873152 | -2.040286 |
| ANG | 0.25152791 | 6.60926278 | 4.27998668 | 3.77E-05 | 3.03E-04 | 1.71086079 |
| SPARCL1 | 0.24028311 | 6.59247442 | 2.51513792 | 0.01321251 | 0.03881176 | -3.7005975 |
| RBP4 | 0.23780364 | 6.63408201 | 3.38837472 | 9.50E-04 | 0.00504093 | -1.3134859 |
| SERPING1 | 0.21389638 | 6.62304219 | 4.28382817 | 3.71E-05 | 3.03E-04 | 1.72504504 |
| RNASE4 | 0.21333547 | 6.65515352 | 4.46477432 | 1.81E-05 | 1.87E-04 | 2.4035917 |
| VTN | 0.21315161 | 6.58356026 | 5.12377394 | 1.15E-06 | 2.10E-05 | 5.03699473 |
| C5 | 0.20978744 | 6.63616915 | 4.57456105 | 1.16E-05 | 1.30E-04 | 2.82503206 |
| SERPINA10 | 0.19835462 | 6.62994692 | 3.4082803 | 8.89E-04 | 0.00479461 | -1.252117 |
| IGFBP4 | 0.19675864 | 6.79299918 | 2.08315007 | 0.03934754 | 0.09116437 | -4.6467107 |
| F10 | 0.19641225 | 6.66436532 | 4.22593489 | 4.65E-05 | 3.47E-04 | 1.51227273 |
| SERPINC1 | 0.19605187 | 6.69497209 | 4.84256398 | 3.84E-06 | 5.05E-05 | 3.88345926 |
| ORM2 | 0.19539747 | 6.61133945 | 2.62935207 | 0.00966734 | 0.03000524 | -3.4236106 |
| F5 | 0.19398626 | 6.6179363 | 3.1658935 | 0.00195737 | 0.00837583 | -1.979005 |
| GPX3 | 0.1908692 | 6.70470252 | 2.60136583 | 0.01044597 | 0.03182152 | -3.4924981 |
| QSOX1 | 0.18079488 | 6.81789015 | 2.70034042 | 0.00792185 | 0.02580485 | -3.2459384 |
| F2 | 0.17596566 | 6.65219628 | 4.52052919 | 1.45E-05 | 1.54E-04 | 2.61671545 |
| IGFALS | 0.17500901 | 6.60116616 | 2.16470398 | 0.03237785 | 0.07949487 | -4.4806172 |
| SERPIND1 | 0.16714979 | 6.62650559 | 3.89565103 | 1.61E-04 | 0.00106047 | 0.34013475 |
| TTR | 0.16564803 | 6.65269695 | 2.29910183 | 0.02321757 | 0.0596764 | -4.1940826 |
| ITIH2 | 0.15256388 | 6.7029171 | 3.16542096 | 0.0019603 | 0.00837583 | -1.9803782 |
| ASGR2 | 0.15065045 | 6.54087934 | 2.05103961 | 0.04242639 | 0.09626403 | -4.7104809 |
| AFM | 0.14757092 | 6.62372208 | 2.61591981 | 0.01003427 | 0.03085303 | -3.4567558 |
| CPN2 | 0.14528786 | 6.69211222 | 2.40421509 | 0.01772692 | 0.04741591 | -3.9589949 |
| SERPINF1 | 0.13670213 | 6.61844429 | 2.84409008 | 0.00523247 | 0.01851058 | -2.8733954 |
| AMBP | 0.12373451 | 6.63466255 | 4.21143026 | 4.91E-05 | 3.59E-04 | 1.45929911 |
| C3 | 0.11551621 | 6.62080047 | 2.82575635 | 0.0055214 | 0.0193249 | -2.9218513 |
| GNPTG | 0.11314935 | 6.72154558 | 2.11287933 | 0.03667022 | 0.08679497 | -4.5868488 |
| ITIH1 | 0.11062795 | 6.63948467 | 2.41607336 | 0.01718628 | 0.04634662 | -3.9318742 |
| PROS1 | 0.10814147 | 6.60773943 | 2.99151544 | 0.003366 | 0.01230461 | -2.4738744 |
| CLU | 0.10699714 | 6.54437279 | 2.42034424 | 0.01699519 | 0.04621005 | -3.9220767 |
| F11 | 0.10500425 | 6.60887061 | 2.12316234 | 0.03578147 | 0.08556992 | -4.5659601 |
| C4BPB | 0.0899855 | 6.65592284 | 2.05987822 | 0.041559 | 0.09561476 | -4.6930197 |
| SERPINF2 | 0.08111389 | 6.65134726 | 2.27724721 | 0.02453107 | 0.06256374 | -4.241755 |
| HABP2 | 0.0795142 | 6.63632475 | 2.03037956 | 0.04451458 | 0.09962786 | -4.7510229 |
| SERPINA6 | -0.0992271 | 6.62118159 | -2.4431652 | 0.01600572 | 0.04500753 | -3.8694595 |
| COLEC10 | -0.1065294 | 6.66609467 | -1.9938562 | 0.04842111 | 0.10646309 | -4.8217563 |
| C8A | -0.1310693 | 6.65440496 | -2.0976758 | 0.0380191 | 0.08871124 | -4.6175607 |
| OLFM1 | -0.1415015 | 6.72965047 | -2.0337759 | 0.04416538 | 0.09952336 | -4.7443844 |
| F13B | -0.1557939 | 6.75107091 | -2.7757257 | 0.00638556 | 0.02188384 | -3.0526855 |
| PON1 | -0.1568683 | 6.59906754 | -2.2685526 | 0.0250715 | 0.06296582 | -4.260605 |
| CHL1 | -0.1590723 | 6.67431086 | -2.5175814 | 0.01312586 | 0.03881176 | -3.6947872 |
| HSPA5 | -0.1603846 | 6.61666521 | -2.9041319 | 0.00438037 | 0.01566457 | -2.7127955 |
| MASP1-iso2 | -0.1759798 | 6.58932379 | -3.1189511 | 0.00226978 | 0.00923602 | -2.1145688 |
| CD14 | -0.1801228 | 6.64724097 | -3.2037443 | 0.00173509 | 0.00815492 | -1.8684518 |
| AOC3 | -0.1981996 | 6.64682556 | -2.4781921 | 0.01458662 | 0.04209649 | -3.7878318 |
| CRISP3 | -0.2043858 | 6.58608 | -2.2739546 | 0.02473452 | 0.06259735 | -4.2489012 |
| IGKV3-20 | -0.204966 | 6.61516208 | -2.0525181 | 0.04228022 | 0.09626403 | -4.7075648 |
| ALB | -0.2100701 | 6.63060051 | -5.3926181 | 3.50E-07 | 1.15E-05 | 6.17825631 |
| PLTP | -0.2188407 | 6.40008748 | -2.5821876 | 0.0110117 | 0.03323715 | -3.5393249 |
| TNXB | -0.228688 | 6.58534931 | -2.8139894 | 0.00571445 | 0.01979003 | -2.952807 |
| APOA2 | -0.2291966 | 6.60087016 | -3.7394288 | 2.83E-04 | 0.00175917 | -0.1886264 |
| GSN | -0.2374417 | 6.68063542 | -4.1355453 | 6.57E-05 | 4.70E-04 | 1.18436072 |
| IGHV3-49 | -0.2408832 | 6.578791 | -2.1350725 | 0.03477539 | 0.08412575 | -4.5416485 |
| IGKV3D-20 | -0.2431479 | 6.60414423 | -2.0989038 | 0.03790859 | 0.08871124 | -4.6150877 |
| PNP | -0.2466268 | 6.59492387 | -2.461799 | 0.01523618 | 0.04321295 | -3.8261655 |
| IGKV3-11 | -0.254279 | 6.60431808 | -2.3507392 | 0.02035688 | 0.05273553 | -4.0797912 |
| IGHV3-7 | -0.2573273 | 6.58422658 | -2.4712482 | 0.01485869 | 0.04250877 | -3.8040975 |
| IGHV3-30 | -0.2688096 | 6.55345126 | -2.632725 | 0.00957713 | 0.03000524 | -3.415264 |
| CLEC3B | -0.2693325 | 6.14626012 | -2.4323469 | 0.0164682 | 0.04591558 | -3.8944586 |
| PCOLCE | -0.2732663 | 6.55082518 | -2.4209756 | 0.0169671 | 0.04621005 | -3.9206271 |
| PON3 | -0.2733847 | 6.50151575 | -1.99279 | 0.0485394 | 0.10646309 | -4.8238032 |
| IGKV4-1 | -0.2761386 | 6.41328804 | -2.3878451 | 0.01849803 | 0.04907946 | -3.9962352 |
| CETP | -0.2788394 | 6.64879413 | -3.1907071 | 0.00180885 | 0.00826544 | -1.9066555 |
| EFEMP1 | -0.2842477 | 6.57939313 | -3.8097136 | 2.20E-04 | 0.00142095 | 0.04717103 |
| PRDX1 | -0.2852195 | 6.60652956 | -2.7377609 | 0.00712165 | 0.02415487 | -3.1505958 |
| ECM1 | -0.2868142 | 6.65571729 | -5.3242572 | 4.76E-07 | 1.30E-05 | 5.88464082 |
| F13A1 | -0.2911951 | 6.62970182 | -4.5693861 | 1.19E-05 | 1.30E-04 | 2.80500527 |
| DPP4 | -0.2991652 | 6.55732685 | -3.9185214 | 1.48E-04 | 9.95E-04 | 0.41895362 |
| IGHG3 | -0.2995957 | 6.58336699 | -3.0737252 | 0.00261395 | 0.00999987 | -2.2435483 |
| LDHA | -0.3042349 | 6.58091833 | -3.5577322 | 5.36E-04 | 0.00297174 | -0.7819931 |
| CAP1 | -0.3070881 | 6.55375053 | -2.0044963 | 0.04725402 | 0.10504442 | -4.8012738 |
| IGHV3-35 | -0.3096866 | 6.52842656 | -2.7321283 | 0.0072372 | 0.02429632 | -3.1650211 |
| IGHG2 | -0.3096936 | 6.59905754 | -3.0301076 | 0.00299107 | 0.0113041 | -2.3664171 |
| IGK-light | -0.3133073 | 6.70623217 | -4.3940578 | 2.41E-05 | 2.26E-04 | 2.13599496 |
| GAPDH | -0.3166657 | 6.58927449 | -2.5506296 | 0.01200281 | 0.03589932 | -3.6157041 |
| IGKV3D-15 | -0.3276384 | 6.67977626 | -2.6408918 | 0.00936183 | 0.0296158 | -3.3950148 |
| IGLV1-51 | -0.3318651 | 6.51662762 | -2.3639596 | 0.01967648 | 0.0517885 | -4.0501579 |
| IGLC3 | -0.3330865 | 6.59242051 | -3.6120331 | 4.44E-04 | 0.00256144 | -0.6071403 |
| ADAMTSL4 | -0.3435603 | 6.69131516 | -4.4348431 | 2.04E-05 | 1.98E-04 | 2.28995624 |
| GGH | -0.3448387 | 6.52479681 | -3.6341859 | 4.11E-04 | 0.00241344 | -0.5351965 |
| SNCB | -0.3450905 | 6.57741113 | -3.1686429 | 0.00194037 | 0.00837583 | -1.971012 |
| CFL1 | -0.3464989 | 6.52668538 | -2.6412738 | 0.00935187 | 0.0296158 | -3.3940663 |
| IGKV3-15 | -0.3519578 | 6.65888892 | -3.6551556 | 3.82E-04 | 0.00228343 | -0.4667704 |
| IGLV3-27 | -0.3605986 | 6.48731666 | -2.4954555 | 0.01392942 | 0.04055557 | -3.7472152 |
| PFN1 | -0.3737577 | 6.4906811 | -2.2202936 | 0.0282648 | 0.07044788 | -4.364026 |
| ITGB1 | -0.3737607 | 6.66077443 | -4.9976417 | 1.98E-06 | 3.11E-05 | 4.51433879 |
| IGLL5 | -0.3811455 | 6.62150307 | -4.712816 | 6.60E-06 | 8.04E-05 | 3.36589177 |
| IGG1-heavy | -0.3855035 | 6.61335088 | -5.2630332 | 6.24E-07 | 1.41E-05 | 5.62363381 |
| IGLV4-69 | -0.3916951 | 6.61894395 | -3.2567101 | 0.00146328 | 0.00718538 | -1.7118993 |
| VCL | -0.3955577 | 6.55408746 | -2.99826 | 0.0032975 | 0.01218963 | -2.4551799 |
| CLIC1 | -0.3967447 | 6.57033099 | -2.4216248 | 0.01693825 | 0.04621005 | -3.919136 |
| CDH5 | -0.3977723 | 6.64567769 | -5.2562573 | 6.43E-07 | 1.41E-05 | 5.59486235 |
| CRTAC1 | -0.398764 | 6.58441955 | -4.4499251 | 1.93E-05 | 1.92E-04 | 2.34714773 |
| HRG | -0.3997916 | 6.56605988 | -5.1567278 | 9.96E-07 | 1.93E-05 | 5.17491833 |
| AHSG | -0.4400166 | 6.57732633 | -5.3700832 | 3.88E-07 | 1.16E-05 | 6.08121509 |
| MMRN1 | -0.4502857 | 6.52138812 | -3.2831638 | 0.00134293 | 0.00678299 | -1.6329057 |
| ACTB | -0.4634068 | 6.522756 | -2.7122048 | 0.00765973 | 0.02545506 | -3.215835 |
| WDR1 | -0.4735804 | 6.52164704 | -3.11747 | 0.00228035 | 0.00923602 | -2.1188182 |
| ACTN1 | -0.4759955 | 6.55838328 | -2.7022698 | 0.00787868 | 0.02580485 | -3.2410508 |
| ACTA1 | -0.4874496 | 6.51674033 | -3.0265881 | 0.00302359 | 0.0113041 | -2.3762661 |
| PKM-iso2 | -0.4983575 | 6.55163871 | -4.2398689 | 4.40E-05 | 3.37E-04 | 1.5632888 |
| FLNA | -0.5024285 | 6.45424243 | -2.9421151 | 0.00390915 | 0.01413309 | -2.609695 |
| ITGA2B | -0.5044682 | 6.60971816 | -3.2820056 | 0.001348 | 0.00678299 | -1.6363754 |
| FERMT3 | -0.5224264 | 6.52525926 | -3.1710094 | 0.00192585 | 0.00837583 | -1.9641275 |
| IGD-heavy | -0.5233118 | 6.47069054 | -2.6908648 | 0.00813702 | 0.02624587 | -3.2698969 |
| LUM | -0.529905 | 6.59831744 | -6.4281607 | 2.69E-09 | 1.77E-07 | 10.879273 |
| ITGB3 | -0.5301196 | 6.53069379 | -3.3232865 | 0.00117791 | 0.0061513 | -1.5120791 |
| RAP1B | -0.5348186 | 6.54762472 | -3.2093504 | 0.00170424 | 0.00812601 | -1.8519837 |
| YWHAZ | -0.536796 | 6.46304584 | -3.1144581 | 0.00230199 | 0.00923602 | -2.1274539 |
| TLN1 | -0.5390143 | 6.44934439 | -3.1045122 | 0.0023748 | 0.00941337 | -2.155921 |
| TPM4 | -0.5474591 | 6.46570815 | -3.1441678 | 0.00209663 | 0.00873152 | -2.0419594 |
| TAGLN2 | -0.551088 | 6.4367834 | -3.2240958 | 0.00162552 | 0.00786465 | -1.8085533 |
| MYH9 | -0.5571363 | 6.46031459 | -3.1921899 | 0.00180032 | 0.00826544 | -1.902317 |
| TUBA1B | -0.5589847 | 6.49044312 | -3.1006479 | 0.00240366 | 0.00941435 | -2.1669605 |
| PDLIM1 | -0.563169 | 6.38142952 | -3.0774556 | 0.00258383 | 0.00999987 | -2.2329701 |
| MYL6 | -0.5699728 | 6.47389833 | -3.1763213 | 0.00189362 | 0.00837583 | -1.9486586 |
| TUBA4A | -0.585857 | 6.46811618 | -3.2791172 | 0.00136072 | 0.00678299 | -1.6450238 |
| CAVIN2 | -0.6225417 | 6.56298517 | -3.554251 | 5.42E-04 | 0.00297174 | -0.7931299 |
| DCD | -0.6746753 | 6.29364025 | -3.9552553 | 1.29E-04 | 9.06E-04 | 0.54629391 |
| SHBG | -0.6750802 | 6.48352799 | -4.8322939 | 4.01E-06 | 5.07E-05 | 3.84214618 |
| DBH | -0.684403 | 6.47189457 | -3.5821768 | 4.92E-04 | 0.00279192 | -0.7035435 |
| COMP | -0.7117384 | 6.57940066 | -7.9588827 | 1.06E-12 | 1.75E-10 | 18.4923181 |
| PI16 | -0.7200206 | 6.50512095 | -5.569082 | 1.58E-07 | 5.76E-06 | 6.94654185 |

logFC: log2 fold change; AveExpr: Average expression; t: moderated t-statistic;P.Value p value; adj.P.Val: adjusted p value (Benjamini-Hochberg); B: Bayes factor

**Supplementary Table 5B. Differential abundant plasma proteins in ALL_GBM (adjusted p value < 0.05 & absolute FC < 0.5)**

| Protein ID | logFC | AveExpr | t | P.Value | adj.P.Val | B |
| --- | --- | --- | --- | --- | --- | --- |
| SAA1 | 1.16782494 | 5.95195677 | 5.08856483 | 1.3404E-06 | 2.2928E-05 | 4.89025537 |
| HP | 0.66201607 | 6.48131445 | 6.79428671 | 4.38E-10 | 4.8034E-08 | 12.6395187 |
| FLNA | -0.5024285 | 6.45424243 | -2.9421151 | 0.00390915 | 0.01413309 | -2.609695 |
| ITGA2B | -0.5044682 | 6.60971816 | -3.2820056 | 0.001348 | 0.00678299 | -1.6363754 |
| FERMT3 | -0.5224264 | 6.52525926 | -3.1710094 | 0.00192585 | 0.00837583 | -1.9641275 |
| IGD-heavy | -0.5233118 | 6.47069054 | -2.6908648 | 0.00813702 | 0.02624587 | -3.2698969 |
| LUM | -0.529905 | 6.59831744 | -6.4281607 | 2.691E-09 | 1.7707E-07 | 10.879273 |
| ITGB3 | -0.5301196 | 6.53069379 | -3.3232865 | 0.00117791 | 0.0061513 | -1.5120791 |
| RAP1B | -0.5348186 | 6.54762472 | -3.2093504 | 0.00170424 | 0.00812601 | -1.8519837 |
| YWHAZ | -0.536796 | 6.46304584 | -3.1144581 | 0.00230199 | 0.00923602 | -2.1274539 |
| TLN1 | -0.5390143 | 6.44934439 | -3.1045122 | 0.0023748 | 0.00941337 | -2.155921 |
| TPM4 | -0.5474591 | 6.46570815 | -3.1441678 | 0.00209663 | 0.00873152 | -2.0419594 |
| TAGLN2 | -0.551088 | 6.4367834 | -3.2240958 | 0.00162552 | 0.00786465 | -1.8085533 |
| MYH9 | -0.5571363 | 6.46031459 | -3.1921899 | 0.00180032 | 0.00826544 | -1.902317 |
| TUBA1B | -0.5589847 | 6.49044312 | -3.1006479 | 0.00240366 | 0.00941435 | -2.1669605 |
| PDLIM1 | -0.563169 | 6.38142952 | -3.0774556 | 0.00258383 | 0.00999987 | -2.2329701 |
| MYL6 | -0.5699728 | 6.47389833 | -3.1763213 | 0.00189362 | 0.00837583 | -1.9486586 |
| TUBA4A | -0.585857 | 6.46811618 | -3.2791172 | 0.00136072 | 0.00678299 | -1.6450238 |
| CAVIN2 | -0.6225417 | 6.56298517 | -3.554251 | 0.00054196 | 0.00297174 | -0.7931299 |
| DCD | -0.6746753 | 6.29364025 | -3.9552553 | 0.00012941 | 0.00090586 | 0.54629391 |
| SHBG | -0.6750802 | 6.48352799 | -4.8322939 | 4.0081E-06 | 5.0718E-05 | 3.84214618 |
| DBH | -0.684403 | 6.47189457 | -3.5821768 | 0.00049219 | 0.00279192 | -0.7035435 |
| COMP | -0.7117384 | 6.57940066 | -7.9588827 | 1.0648E-12 | 1.7516E-10 | 18.4923181 |
| PI16 | -0.7200206 | 6.50512095 | -5.569082 | 1.5767E-07 | 5.7639E-06 | 6.94654185 |

logFC: log2 fold change; AveExpr: Average expression; t: moderated t-statistic;P.Value p value; adj.P.Val: adjusted p value (Benjamini-Hochberg); B: Bayes factor

**Supplementary Table 6A. Patients characteristics in the validation cohort**

| Sample ID | Date diagnosis | Age | Sex | Resection | Date Sample | Event |
| --- | --- | --- | --- | --- | --- | --- |
| GBM3 | 01/12/2023 | 63 | male | complete | 01/12/2023 | pre surgery |
| GBM3 | 01/12/2023 | 63 | male | complete | 13/12/2023 | before oncological tx |
| GBM3 | 01/12/2023 | 63 | male | complete | 03/01/2024 | RT/TMZ day 15 |
| GBM3 | 01/12/2023 | 63 | male | complete | 29/02/2024 | adj TMZ course 1 |
| GBM4 | 19/01/2024 | 58 | female | complete | 19/01/2024 | pre surgery |
| GBM4 | 19/01/2024 | 58 | female | complete | 02/02/2024 | before oncological tx |
| GBM4 | 19/01/2024 | 58 | female | complete | 27/02/2024 | RT/TMZ day 15 |
| GBM4 | 19/01/2024 | 58 | female | complete | 26/04/2024 | adj TMZ course 1 |
| GBM5 | 22/01/2024 | 62 | male | complete | 22/01/2024 | pre surgery |
| GBM5 | 22/01/2024 | 62 | male | complete | 22/05/2024 | adj TMZ course 2 |
| GBM6 | 26/01/2024 | 61 | male | complete | 26/01/2024 | pre surgery |
| GBM6 | 26/01/2024 | 61 | male | complete | 09/02/2024 | before oncological tx |
| GBM6 | 26/01/2024 | 61 | male | complete | 04/03/2024 | RT/TMZ day 15 |
| GBM6 | 26/01/2024 | 61 | male | complete | 30/04/2024 | adj TMZ course 1 |
| GBM6 | 26/01/2024 | 61 | male | complete | 08/10/2024 | adj TMZ course 6 |
| GBM7 | 29/01/2024 | 65 | male | partial | 29/01/2024 | pre surgery |
| GBM7 | 29/01/2024 | 65 | male | partial | 12/02/2024 | before oncological tx |
| GBM7 | 29/01/2024 | 65 | male | partial | 05/03/2024 | RT/TMZ day 15 |
| GBM7 | 29/01/2024 | 65 | male | partial | 30/04/2024 | adj TMZ course 1 |
| GBM7 | 29/01/2024 | 65 | male | partial | 24/09/2024 | adj TMZ course 6 |
| GBM8 | 02/02/2024 | 51 | male | complete | 02/02/2024 | pre surgery |
| GBM8 | 02/02/2024 | 51 | male | complete | 16/02/2024 | before oncological tx |
| GBM8 | 02/02/2024 | 51 | male | complete | 12/03/2024 | RT/TMZ day 15 |
| GBM8 | 02/02/2024 | 51 | male | complete | 08/05/2024 | adj TMZ course 1 |
| GBM8 | 02/02/2024 | 51 | male | complete | 07/10/2024 | adj TMZ course 6 |
| GBM9 | 14/02/2024 | 54 | male | complete | 14/02/2024 | pre surgery |
| GBM9 | 14/02/2024 | 54 | male | complete | 27/02/2024 | before oncological tx |
| GBM9 | 14/02/2024 | 54 | male | complete | 19/03/2024 | RT/TMZ day 15 |
| GBM9 | 14/02/2024 | 54 | male | complete | 14/05/2024 | adj TMZ course 1 |
| GBM9 | 14/02/2024 | 54 | male | complete | 30/09/2024 | adj TMZ course 6 |
| GBM10 | 28/02/2024 | 51 | male | complete | 28/02/2024 | pre surgery |
| GBM10 | 28/02/2024 | 51 | male | complete | 12/03/2024 | before oncological tx |
| GBM10 | 28/02/2024 | 51 | male | complete | 03/04/2024 | RT/TMZ day 15 |
| GBM10 | 28/02/2024 | 51 | male | complete | 31/05/2024 | adj TMZ course 1 |
| GBM10 | 28/02/2024 | 51 | male | complete | 29/10/2024 | adj TMZ course 6 |
| GBM11 | 28/02/2024 | 64 | male | complete | 28/02/2024 | pre surgery |
| GBM11 | 28/02/2024 | 64 | male | complete | 11/03/2024 | before oncological tx |
| GBM11 | 28/02/2024 | 64 | male | complete | 02/04/2024 | RT/TMZ day 15 |
| GBM11 | 28/02/2024 | 64 | male | complete | 29/05/2024 | adj TMZ course 1 |
| GBM12 | 13/03/2024 | 62 | male | complete | 13/03/2024 | pre surgery |
| GBM12 | 13/03/2024 | 62 | male | complete | 22/04/2024 | before oncological tx |
| GBM12 | 13/03/2024 | 62 | male | complete | 06/05/2024 | RT/TMZ day 15 |
| GBM12 | 13/03/2024 | 62 | male | complete | 15/07/2024 | adj TMZ course 1 |
| GBM12 | 13/03/2024 | 62 | male | complete | 29/11/2024 | adj TMZ course 6 |
| GBM13 | 10/04/2024 | 65 | female | complete | 10/04/2024 | pre surgery |
| GBM13 | 10/04/2024 | 65 | female | complete | 19/04/2024 | before oncological tx |
| GBM13 | 10/04/2024 | 65 | female | complete | 13/05/2024 | RT/TMZ day 15 |
| GBM13 | 10/04/2024 | 65 | female | complete | 09/07/2024 | adj TMZ course 1 |
| GBM13 | 10/04/2024 | 66 | female | complete | 02/10/2024 | adj TMZ course 4 |
| GBM14 | 13/08/2024 | 61 | male | complete | 13/08/2024 | pre surgery |
| GBM14 | 13/08/2024 | 61 | male | complete | 27/08/2024 | before oncological tx |
| GBM14 | 13/08/2024 | 61 | male | complete | 12/09/2024 | RT/TMZ day 15 |
| GBM14 | 13/08/2024 | 61 | male | complete | 13/11/2024 | adj TMZ course 1 |
| GBM14 | 13/08/2024 | 61 | male | complete | 07/02/2025 | adj TMZ course 4 |
| GBM15 | 24/09/2024 | 58 | female | complete | 24/09/2024 | pre surgery |
| GBM15 | 24/09/2024 | 58 | female | complete | 02/10/2024 | before oncological tx |
| GBM15 | 24/09/2024 | 58 | female | complete | 23/10/2024 | RT/TMZ day 15 |
| GBM15 | 24/09/2024 | 58 | female | complete | 13/01/2025 | adj TMZ course 2 (not given) |

**Supplementary Table 6B. Relative Flourescence Unit (RFU) Data**

| SubjectID | TP | Aptamer SeqId | RFU | RFU_log2 | Protein | event |
| --- | --- | --- | --- | --- | --- | --- |
| GBM10 | TP1 | seq.4876.32 | 33563.6 | 15.0346528 | F9a | pre surgery |
| GBM10 | TP1 | seq.5307.12 | 17981.2 | 14.1342819 | F9b | pre surgery |
| GBM10 | TP1 | seq.8043.153 | 717.7 | 9.48924588 | COMP | pre surgery |
| GBM11 | TP1 | seq.4876.32 | 30980.3 | 14.9191101 | F9a | pre surgery |
| GBM11 | TP1 | seq.5307.12 | 16974.8 | 14.0511919 | F9b | pre surgery |
| GBM11 | TP1 | seq.8043.153 | 1041.5 | 10.0258317 | COMP | pre surgery |
| GBM12 | TP1 | seq.4876.32 | 33371.3 | 15.0263635 | F9a | pre surgery |
| GBM12 | TP1 | seq.5307.12 | 19033.5 | 14.2163291 | F9b | pre surgery |
| GBM12 | TP1 | seq.8043.153 | 770.8 | 9.59208323 | COMP | pre surgery |
| GBM13 | TP1 | seq.4876.32 | 41277.8 | 15.3331134 | F9a | pre surgery |
| GBM13 | TP1 | seq.5307.12 | 22332.8 | 14.4469411 | F9b | pre surgery |
| GBM13 | TP1 | seq.8043.153 | 674.2 | 9.39917109 | COMP | pre surgery |
| GBM14 | TP1 | seq.4876.32 | 31081.7 | 14.9238242 | F9a | pre surgery |
| GBM14 | TP1 | seq.5307.12 | 16709.3 | 14.02845 | F9b | pre surgery |
| GBM14 | TP1 | seq.8043.153 | 727.5 | 9.50878516 | COMP | pre surgery |
| GBM15 | TP1 | seq.4876.32 | 35276.1 | 15.1064444 | F9a | pre surgery |
| GBM15 | TP1 | seq.5307.12 | 18149.1 | 14.1476899 | F9b | pre surgery |
| GBM15 | TP1 | seq.8043.153 | 993 | 9.95710204 | COMP | pre surgery |
| GBM3 | TP1 | seq.4876.32 | 25227.8 | 14.622784 | F9a | pre surgery |
| GBM3 | TP1 | seq.5307.12 | 13831.9 | 13.755816 | F9b | pre surgery |
| GBM3 | TP1 | seq.8043.153 | 989.8 | 9.95245006 | COMP | pre surgery |
| GBM4 | TP1 | seq.4876.32 | 30503.4 | 14.8967297 | F9a | pre surgery |
| GBM4 | TP1 | seq.5307.12 | 16613.5 | 14.0201553 | F9b | pre surgery |
| GBM4 | TP1 | seq.8043.153 | 860.4 | 9.75053951 | COMP | pre surgery |
| GBM5 | TP1 | seq.4876.32 | 35930.5 | 15.1329615 | F9a | pre surgery |
| GBM5 | TP1 | seq.5307.12 | 20024.3 | 14.2895362 | F9b | pre surgery |
| GBM5 | TP1 | seq.8043.153 | 1069 | 10.0633951 | COMP | pre surgery |
| GBM6 | TP1 | seq.4876.32 | 29313.6 | 14.8393318 | F9a | pre surgery |
| GBM6 | TP1 | seq.5307.12 | 16034.1 | 13.9689457 | F9b | pre surgery |
| GBM6 | TP1 | seq.8043.153 | 1039.4 | 10.0229226 | COMP | pre surgery |
| GBM7 | TP1 | seq.4876.32 | 32388 | 14.9832163 | F9a | pre surgery |
| GBM7 | TP1 | seq.5307.12 | 18339.7 | 14.1627611 | F9b | pre surgery |
| GBM7 | TP1 | seq.8043.153 | 654 | 9.3553511 | COMP | pre surgery |
| GBM8 | TP1 | seq.4876.32 | 35392.9 | 15.1112131 | F9a | pre surgery |
| GBM8 | TP1 | seq.5307.12 | 19813.9 | 14.2742981 | F9b | pre surgery |
| GBM8 | TP1 | seq.8043.153 | 610 | 9.25502857 | COMP | pre surgery |
| GBM9 | TP1 | seq.4876.32 | 34600.7 | 15.0785553 | F9a | pre surgery |
| GBM9 | TP1 | seq.5307.12 | 19580.5 | 14.2572037 | F9b | pre surgery |
| GBM9 | TP1 | seq.8043.153 | 662.7 | 9.37438746 | COMP | pre surgery |
| GBM10 | TP2 | seq.4876.32 | 27141 | 14.7282394 | F9a | before oncological tx |
| GBM10 | TP2 | seq.5307.12 | 15052.8 | 13.8778401 | F9b | before oncological tx |
| GBM10 | TP2 | seq.8043.153 | 967 | 9.91886324 | COMP | before oncological tx |
| GBM11 | TP2 | seq.4876.32 | 31377.4 | 14.9374842 | F9a | before oncological tx |
| GBM11 | TP2 | seq.5307.12 | 16321 | 13.9945302 | F9b | before oncological tx |
| GBM11 | TP2 | seq.8043.153 | 1058.9 | 10.0497124 | COMP | before oncological tx |
| GBM12 | TP2 | seq.4876.32 | 28395.3 | 14.7934153 | F9a | before oncological tx |
| GBM12 | TP2 | seq.5307.12 | 15119 | 13.8841705 | F9b | before oncological tx |
| GBM12 | TP2 | seq.8043.153 | 921 | 9.84862294 | COMP | before oncological tx |
| GBM13 | TP2 | seq.4876.32 | 33844.8 | 15.0466892 | F9a | before oncological tx |
| GBM13 | TP2 | seq.5307.12 | 18588.5 | 14.1822004 | F9b | before oncological tx |
| GBM13 | TP2 | seq.8043.153 | 554.3 | 9.11712359 | COMP | before oncological tx |
| GBM14 | TP2 | seq.4876.32 | 27561.8 | 14.7504348 | F9a | before oncological tx |
| GBM14 | TP2 | seq.5307.12 | 15328.1 | 13.9039854 | F9b | before oncological tx |
| GBM14 | TP2 | seq.8043.153 | 749.2 | 9.55113145 | COMP | before oncological tx |
| GBM15 | TP2 | seq.4876.32 | 32926.5 | 15.0070054 | F9a | before oncological tx |
| GBM15 | TP2 | seq.5307.12 | 17969.5 | 14.1333429 | F9b | before oncological tx |
| GBM15 | TP2 | seq.8043.153 | 758.1 | 9.56814614 | COMP | before oncological tx |
| GBM3 | TP2 | seq.4876.32 | 25423.6 | 14.6339375 | F9a | before oncological tx |
| GBM3 | TP2 | seq.5307.12 | 13990.5 | 13.772263 | F9b | before oncological tx |
| GBM3 | TP2 | seq.8043.153 | 1222 | 10.2562087 | COMP | before oncological tx |
| GBM4 | TP2 | seq.4876.32 | 30453.8 | 14.894382 | F9a | before oncological tx |
| GBM4 | TP2 | seq.5307.12 | 16339.6 | 13.9961733 | F9b | before oncological tx |
| GBM4 | TP2 | seq.8043.153 | 672.4 | 9.39531991 | COMP | before oncological tx |
| GBM6 | TP2 | seq.4876.32 | 26774.4 | 14.7086205 | F9a | before oncological tx |
| GBM6 | TP2 | seq.5307.12 | 15143.4 | 13.8864968 | F9b | before oncological tx |
| GBM6 | TP2 | seq.8043.153 | 930.8 | 9.86387652 | COMP | before oncological tx |
| GBM7 | TP2 | seq.4876.32 | 35434.7 | 15.1129159 | F9a | before oncological tx |
| GBM7 | TP2 | seq.5307.12 | 19832.2 | 14.2756299 | F9b | before oncological tx |
| GBM7 | TP2 | seq.8043.153 | 642.7 | 9.33024466 | COMP | before oncological tx |
| GBM8 | TP2 | seq.4876.32 | 29728.6 | 14.8596124 | F9a | before oncological tx |
| GBM8 | TP2 | seq.5307.12 | 16566.2 | 14.0160422 | F9b | before oncological tx |
| GBM8 | TP2 | seq.8043.153 | 801.9 | 9.6490765 | COMP | before oncological tx |
| GBM9 | TP2 | seq.4876.32 | 32278.7 | 14.9783396 | F9a | before oncological tx |
| GBM9 | TP2 | seq.5307.12 | 18245.1 | 14.1553005 | F9b | before oncological tx |
| GBM9 | TP2 | seq.8043.153 | 755.5 | 9.56319627 | COMP | before oncological tx |
| GBM10 | TP3 | seq.4876.32 | 29529.3 | 14.8499084 | F9a | RT/TMZ day 15 |
| GBM10 | TP3 | seq.5307.12 | 13959.4 | 13.7690527 | F9b | RT/TMZ day 15 |
| GBM10 | TP3 | seq.8043.153 | 1287.3 | 10.3312529 | COMP | RT/TMZ day 15 |
| GBM11 | TP3 | seq.4876.32 | 30899.4 | 14.9153379 | F9a | RT/TMZ day 15 |
| GBM11 | TP3 | seq.5307.12 | 15974.7 | 13.9635915 | F9b | RT/TMZ day 15 |
| GBM11 | TP3 | seq.8043.153 | 1117 | 10.1267045 | COMP | RT/TMZ day 15 |
| GBM12 | TP3 | seq.4876.32 | 31793.9 | 14.9565078 | F9a | RT/TMZ day 15 |
| GBM12 | TP3 | seq.5307.12 | 16655.9 | 14.0238323 | F9b | RT/TMZ day 15 |
| GBM12 | TP3 | seq.8043.153 | 918.2 | 9.84423499 | COMP | RT/TMZ day 15 |
| GBM13 | TP3 | seq.4876.32 | 28096.3 | 14.7781439 | F9a | RT/TMZ day 15 |
| GBM13 | TP3 | seq.5307.12 | 15684.4 | 13.9371347 | F9b | RT/TMZ day 15 |
| GBM13 | TP3 | seq.8043.153 | 777.7 | 9.60492382 | COMP | RT/TMZ day 15 |
| GBM14 | TP3 | seq.4876.32 | 24134.7 | 14.5588811 | F9a | RT/TMZ day 15 |
| GBM14 | TP3 | seq.5307.12 | 13405.4 | 13.7106343 | F9b | RT/TMZ day 15 |
| GBM14 | TP3 | seq.8043.153 | 792.7 | 9.63245 | COMP | RT/TMZ day 15 |
| GBM15 | TP3 | seq.4876.32 | 28886.4 | 14.8181527 | F9a | RT/TMZ day 15 |
| GBM15 | TP3 | seq.5307.12 | 15807.2 | 13.9483855 | F9b | RT/TMZ day 15 |
| GBM15 | TP3 | seq.8043.153 | 967.6 | 9.91975719 | COMP | RT/TMZ day 15 |
| GBM3 | TP3 | seq.4876.32 | 28172.5 | 14.7820512 | F9a | RT/TMZ day 15 |
| GBM3 | TP3 | seq.5307.12 | 14567 | 13.8305152 | F9b | RT/TMZ day 15 |
| GBM3 | TP3 | seq.8043.153 | 1202.7 | 10.2332602 | COMP | RT/TMZ day 15 |
| GBM4 | TP3 | seq.4876.32 | 22948 | 14.4861437 | F9a | RT/TMZ day 15 |
| GBM4 | TP3 | seq.5307.12 | 11468.7 | 13.48554 | F9b | RT/TMZ day 15 |
| GBM4 | TP3 | seq.8043.153 | 1054.3 | 10.0434375 | COMP | RT/TMZ day 15 |
| GBM6 | TP3 | seq.4876.32 | 27280.2 | 14.7356195 | F9a | RT/TMZ day 15 |
| GBM6 | TP3 | seq.5307.12 | 14304 | 13.8042319 | F9b | RT/TMZ day 15 |
| GBM6 | TP3 | seq.8043.153 | 1149.7 | 10.168296 | COMP | RT/TMZ day 15 |
| GBM7 | TP3 | seq.4876.32 | 30905.5 | 14.9156227 | F9a | RT/TMZ day 15 |
| GBM7 | TP3 | seq.5307.12 | 17162.5 | 14.0670562 | F9b | RT/TMZ day 15 |
| GBM7 | TP3 | seq.8043.153 | 776.9 | 9.6034409 | COMP | RT/TMZ day 15 |
| GBM8 | TP3 | seq.4876.32 | 27490 | 14.7466718 | F9a | RT/TMZ day 15 |
| GBM8 | TP3 | seq.5307.12 | 14441.7 | 13.8180529 | F9b | RT/TMZ day 15 |
| GBM8 | TP3 | seq.8043.153 | 845 | 9.72451385 | COMP | RT/TMZ day 15 |
| GBM9 | TP3 | seq.4876.32 | 30741.4 | 14.9079422 | F9a | RT/TMZ day 15 |
| GBM9 | TP3 | seq.5307.12 | 16273.3 | 13.9903079 | F9b | RT/TMZ day 15 |
| GBM9 | TP3 | seq.8043.153 | 748.7 | 9.55016959 | COMP | RT/TMZ day 15 |
| GBM10 | TP4 | seq.4876.32 | 25010.3 | 14.6102924 | F9a | adj TMZ course 1 |
| GBM10 | TP4 | seq.5307.12 | 13155.3 | 13.6834662 | F9b | adj TMZ course 1 |
| GBM10 | TP4 | seq.8043.153 | 1563.1 | 10.611117 | COMP | adj TMZ course 1 |
| GBM11 | TP4 | seq.4876.32 | 29432.3 | 14.8451617 | F9a | adj TMZ course 1 |
| GBM11 | TP4 | seq.5307.12 | 16557.2 | 14.0152582 | F9b | adj TMZ course 1 |
| GBM11 | TP4 | seq.8043.153 | 988.5 | 9.9505559 | COMP | adj TMZ course 1 |
| GBM12 | TP4 | seq.4876.32 | 27056.1 | 14.7237196 | F9a | adj TMZ course 1 |
| GBM12 | TP4 | seq.5307.12 | 14885.1 | 13.8616782 | F9b | adj TMZ course 1 |
| GBM12 | TP4 | seq.8043.153 | 1351.9 | 10.4018395 | COMP | adj TMZ course 1 |
| GBM13 | TP4 | seq.4876.32 | 31780.6 | 14.9559041 | F9a | adj TMZ course 1 |
| GBM13 | TP4 | seq.5307.12 | 17339.9 | 14.0818912 | F9b | adj TMZ course 1 |
| GBM13 | TP4 | seq.8043.153 | 548.3 | 9.10145048 | COMP | adj TMZ course 1 |
| GBM14 | TP4 | seq.4876.32 | 28855.1 | 14.8165887 | F9a | adj TMZ course 1 |
| GBM14 | TP4 | seq.5307.12 | 16144 | 13.9787998 | F9b | adj TMZ course 1 |
| GBM14 | TP4 | seq.8043.153 | 804.3 | 9.65338252 | COMP | adj TMZ course 1 |
| GBM3 | TP4 | seq.4876.32 | 26731.8 | 14.7063233 | F9a | adj TMZ course 1 |
| GBM3 | TP4 | seq.5307.12 | 13954.1 | 13.7685048 | F9b | adj TMZ course 1 |
| GBM3 | TP4 | seq.8043.153 | 1490.1 | 10.5421613 | COMP | adj TMZ course 1 |
| GBM4 | TP4 | seq.4876.32 | 27468.8 | 14.7455588 | F9a | adj TMZ course 1 |
| GBM4 | TP4 | seq.5307.12 | 13981.3 | 13.7713141 | F9b | adj TMZ course 1 |
| GBM4 | TP4 | seq.8043.153 | 969 | 9.92184094 | COMP | adj TMZ course 1 |
| GBM6 | TP4 | seq.4876.32 | 26370.1 | 14.6866701 | F9a | adj TMZ course 1 |
| GBM6 | TP4 | seq.5307.12 | 14067.6 | 13.7801912 | F9b | adj TMZ course 1 |
| GBM6 | TP4 | seq.8043.153 | 1193.5 | 10.2221911 | COMP | adj TMZ course 1 |
| GBM7 | TP4 | seq.4876.32 | 29400.7 | 14.843612 | F9a | adj TMZ course 1 |
| GBM7 | TP4 | seq.5307.12 | 15836.8 | 13.9510843 | F9b | adj TMZ course 1 |
| GBM7 | TP4 | seq.8043.153 | 763.4 | 9.57818397 | COMP | adj TMZ course 1 |
| GBM8 | TP4 | seq.4876.32 | 25035.5 | 14.6117453 | F9a | adj TMZ course 1 |
| GBM8 | TP4 | seq.5307.12 | 13654.4 | 13.737184 | F9b | adj TMZ course 1 |
| GBM8 | TP4 | seq.8043.153 | 1158.2 | 10.1789138 | COMP | adj TMZ course 1 |
| GBM9 | TP4 | seq.4876.32 | 32282.5 | 14.9785094 | F9a | adj TMZ course 1 |
| GBM9 | TP4 | seq.5307.12 | 17686 | 14.1104017 | F9b | adj TMZ course 1 |
| GBM9 | TP4 | seq.8043.153 | 759.3 | 9.57042498 | COMP | adj TMZ course 1 |
| GBM5 | TP5 | seq.4876.32 | 32406.4 | 14.9840357 | F9a | adj TMZ course 2 |
| GBM5 | TP5 | seq.5307.12 | 16438.6 | 14.0048876 | F9b | adj TMZ course 2 |
| GBM5 | TP5 | seq.8043.153 | 1191.2 | 10.2194106 | COMP | adj TMZ course 2 |
| GBM15 | TP5 | seq.4876.32 | 30193.9 | 14.8820173 | F9a | adj TMZ course 2 (not given) |
| GBM15 | TP5 | seq.5307.12 | 17118 | 14.0633108 | F9b | adj TMZ course 2 (not given) |
| GBM15 | TP5 | seq.8043.153 | 956.2 | 9.90267659 | COMP | adj TMZ course 2 (not given) |
| GBM13 | TP6 | seq.4876.32 | 37012.5 | 15.1757639 | F9a | adj TMZ course 4 |
| GBM13 | TP6 | seq.5307.12 | 19349.2 | 14.2400609 | F9b | adj TMZ course 4 |
| GBM13 | TP6 | seq.8043.153 | 423.3 | 8.72894087 | COMP | adj TMZ course 4 |
| GBM14 | TP6 | seq.4876.32 | 29616.2 | 14.8541476 | F9a | adj TMZ course 4 |
| GBM14 | TP6 | seq.5307.12 | 16506.6 | 14.0108428 | F9b | adj TMZ course 4 |
| GBM14 | TP6 | seq.8043.153 | 714.8 | 9.48341273 | COMP | adj TMZ course 4 |
| GBM10 | TP7 | seq.4876.32 | 25091.4 | 14.6149629 | F9a | adj TMZ course 6 |
| GBM10 | TP7 | seq.5307.12 | 13724.9 | 13.7446131 | F9b | adj TMZ course 6 |
| GBM10 | TP7 | seq.8043.153 | 1271.2 | 10.3131098 | COMP | adj TMZ course 6 |
| GBM12 | TP7 | seq.4876.32 | 28309.7 | 14.7890598 | F9a | adj TMZ course 6 |
| GBM12 | TP7 | seq.5307.12 | 15455.5 | 13.9159261 | F9b | adj TMZ course 6 |
| GBM12 | TP7 | seq.8043.153 | 1149.7 | 10.168296 | COMP | adj TMZ course 6 |
| GBM6 | TP7 | seq.4876.32 | 24771.1 | 14.5964286 | F9a | adj TMZ course 6 |
| GBM6 | TP7 | seq.5307.12 | 13232.8 | 13.6919398 | F9b | adj TMZ course 6 |
| GBM6 | TP7 | seq.8043.153 | 1235.8 | 10.2723965 | COMP | adj TMZ course 6 |
| GBM7 | TP7 | seq.4876.32 | 30909.3 | 14.9158 | F9a | adj TMZ course 6 |
| GBM7 | TP7 | seq.5307.12 | 17122.5 | 14.06369 | F9b | adj TMZ course 6 |
| GBM7 | TP7 | seq.8043.153 | 617.3 | 9.2721632 | COMP | adj TMZ course 6 |
| GBM8 | TP7 | seq.4876.32 | 28139.1 | 14.7803398 | F9a | adj TMZ course 6 |
| GBM8 | TP7 | seq.5307.12 | 14424.2 | 13.8163037 | F9b | adj TMZ course 6 |
| GBM8 | TP7 | seq.8043.153 | 975.4 | 9.93132849 | COMP | adj TMZ course 6 |
| GBM9 | TP7 | seq.4876.32 | 32535.3 | 14.9897626 | F9a | adj TMZ course 6 |
| GBM9 | TP7 | seq.5307.12 | 17646.8 | 14.1072007 | F9b | adj TMZ course 6 |
| GBM9 | TP7 | seq.8043.153 | 860.3 | 9.75037202 | COMP | adj TMZ course 6 |

TP: time point; tx: treatment ; RT/TMZ: mid-radio(RT)-chemotherapy with Temozolomide; adj TMZ: adjuvant temozolomide

**Supplementary Table 6C. Mixed-effects linear model results for F9a**

| Effect | Term | Estimate | Std.error | Statistic | DF | p value | Significance |
| --- | --- | --- | --- | --- | --- | --- | --- |
| fixed | (Intercept) | 15.0006385 | 0.04181973 | 358.697605 | 28.6194407 | 7.91E-54 |  |
| fixed | TP2 | -0.1229449 | 0.04392575 | -2.798925 | 39.6661883 | 0.00787795 | ** |
| fixed | TP3 | -0.206286 | 0.04392575 | -4.6962434 | 39.6661883 | 3.15E-05 | *** |
| fixed | TP4 | -0.2134128 | 0.04523804 | -4.7175521 | 40.1032773 | 2.89E-05 | *** |
| fixed | TP5 | -0.1353104 | 0.09249141 | -1.4629506 | 46.3738637 | 0.15022599 |  |
| fixed | TP6 | -0.0096289 | 0.08859757 | -0.1086811 | 41.8534075 | 0.9139748 |  |
| fixed | TP7 | -0.2216044 | 0.05586698 | -3.9666435 | 40.9234256 | 2.87E-04 | *** |

**Supplementary Table 6D. Mixed-effects linear model results for F9b**

| Effect | Term | Estimate | Std.error | Statistic | DF | p value | Significance |
| --- | --- | --- | --- | --- | --- | --- | --- |
| fixed | (Intercept) | 14.1348923 | 0.04396589 | 321.496762 | 24.4819655 | 6.64E-46 |  |
| fixed | TP2 | -0.1225337 | 0.04190162 | -2.9243183 | 39.7390097 | 0.00567703 | ** |
| fixed | TP3 | -0.2666704 | 0.04190162 | -6.3642017 | 39.7390097 | 1.49E-07 | *** |
| fixed | TP4 | -0.2371103 | 0.0431872 | -5.490292 | 40.0962855 | 2.44E-06 | *** |
| fixed | TP5 | -0.1687072 | 0.08937 | -1.8877385 | 45.303136 | 0.06547637 | . |
| fixed | TP6 | -0.0561355 | 0.08482495 | -0.6617802 | 41.2770542 | 0.51178985 |  |
| fixed | TP7 | -0.2528888 | 0.05340596 | -4.7352164 | 40.6499127 | 2.66E-05 | *** |

**Supplementary Table 6E. Mixed-effects linear model results for COMP**

| Effect | Term | Estimate | Std.error | Statistic | DF | p value | Significance |
| --- | --- | --- | --- | --- | --- | --- | --- |
| fixed | (Intercept) | 9.6727918 | 0.09884654 | 97.8566572 | 20.1958305 | 1.64E-28 |  |
| fixed | TP2 | 0.02687152 | 0.08259604 | 0.32533671 | 39.3478055 | 0.74664995 |  |
| fixed | TP3 | 0.24944811 | 0.08259604 | 3.02009776 | 39.3478055 | 0.00442294 | ** |
| fixed | TP4 | 0.33176556 | 0.08519452 | 3.8942123 | 39.6276858 | 3.69E-04 | *** |
| fixed | TP5 | 0.22921707 | 0.17835133 | 1.28519961 | 43.7704918 | 0.20548162 |  |
| fixed | TP6 | -0.2291242 | 0.16771473 | -1.3661546 | 40.3864176 | 0.17944976 |  |
| fixed | TP7 | 0.30625796 | 0.10546537 | 2.90387222 | 39.9835743 | 0.00597506 | ** |

Signif. codes: 0 ‘***’ 0.001 ‘**’ 0.01 ‘*’ 0.05 ‘.’ 0.1 ‘ ’ 1
